# Reassessing the Possibility of π–σ–π Full Electron Delocalization Through 3D Aromatic Carboranes

**DOI:** 10.1002/chem.202501806

**Published:** 2025-06-27

**Authors:** Yanhong Gao, Balázs Szathmári, Dániel Buzsáki, Zsolt Kelemen

**Affiliations:** ^1^ Department of Inorganic and Analytical Chemistry Budapest University of Technology and Economics Műegyetem rkp. 3 Budapest H‐1111 Hungary; ^2^ HUN‐REN Wigner Research Centre for Physics P.O. Box 49 Budapest H‐1525 Hungary; ^3^ HUN‐REN Computation Driven Chemistry Research Group Budapest University of Technology and Economics Műegyetem rkp. 3 Budapest H‐1111 Hungary

**Keywords:** aromaticity, borane clusters, DFT calculations, hyperconjugation

## Abstract

Over the past few decades, considerable attention has been devoted to explore the electronic interactions of various substituents with 3D aromatic carboranes. In the case of substituents possessing available p‐orbitals, conjugation between the π‐system and the σ‐framework of the carborane is possible via negative hyperconjugation, where electron density from a filled π‐orbital overlaps with an adjacent σ* orbital. However, this is inherently a local effect, which was further bolstered by the fact that aromatic conjugation between planar (2D) π‐systems and the 3D aromatic carborane cage does not exist. Nevertheless, recent studies have raised the intriguing possibility that 3D aromatic carboranes may function as σ‐conjugated bridges, enabling electronic communication between spatially separated π‐systems. Herein, we investigated the potential conjugation of various substituents with or through carborane systems to better understand the nature of the possible interaction. We demonstrated that significant delocalization through the carborane cluster can be achieved only in the case of radical systems; however, this type of interaction is not a unique feature of carboranes, as similar behavior can also be observed in saturated hydrocarbons.

## Graphical abstract

Aromatic carborane clusters interact with π‐systems primarily through local negative hyperconjugation. This interaction is highly dependent on the cluster's size and symmetry. Significant through‐cage delocalization occurs mainly in radical species, though similar behavior can also be observed in simple hydrocarbons.

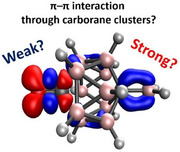

## 1 Introduction

**Figure 1:**
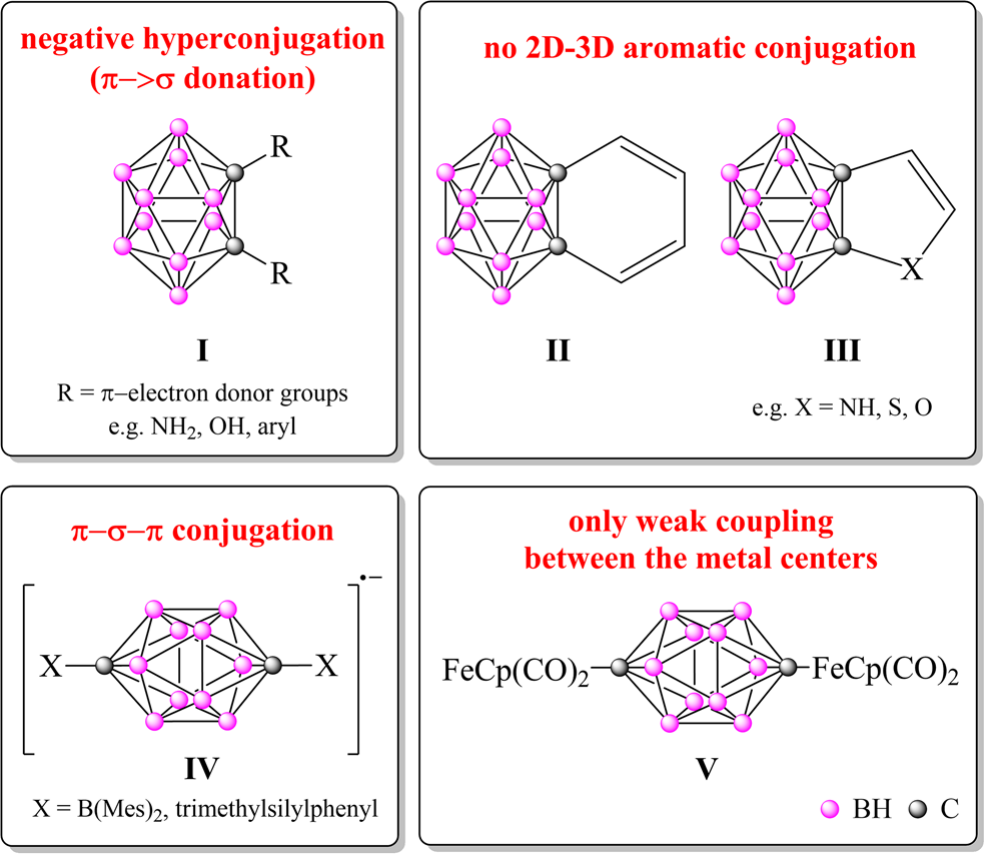
Conjugative interaction in the case of carborane‐based systems.

Conjugative interactions are crucial for understanding the stability, reactivity, and geometries of molecules. The conjugation of π‐electrons and π‐aromaticity are well‐established textbook concepts. However, conjugation is not exclusive to π‐systems; conjugation of σ‐bonds is also a recognized phenomenon, and the concept of σ‐aromaticity is well‐founded.^[^ ^1^ ^]^ In addition to these, hyperconjugation—an interaction between σ‐ and π‐systems—also plays a significant role in the stabilization of various systems.^[^ ^2^ ^]^ The significance of these interactions is undeniable, as they act as a bridge between π and σ systems. While the interaction between π‐orbitals leads to long‐range orbital communication through π‐chains, hyperconjugation can be considered rather as a local effect, even if it can be extended to a distance of a few σ‐bonds (e.g., double hyperconjugation).^[^ ^3^, ^4^ ^]^ The concept of conjugation between 2D and 3D aromatic systems, which shows certain similarity to the concept of hyperconjugation (**I** Figure 1), was proposed during the last decade in the case of carborane‐fused 2D ring systems (**II**‐**III** Figure 1).^[^ ^5^, ^6^, ^7^, ^8^, ^9^, ^10^ ^]^ Despite the promising results, Poater, Teixidor, and Solà demonstrated that such an interaction is hindered by the poor overlap between the orbitals of the 2D and 3D units.^[^ ^11^ ^]^ We have clearly shown that the proposed aromatic character arises from a misleading interpretation of nucleus‐independent chemical shift (NICS) indices^[^ ^12^ ^]^; therefore, such aromatic conjugation does not exist between the two different types of aromatic systems.^[^ ^12^, ^13^, ^14^, ^15^, ^16^ ^]^ On the other hand, the possibility that 3D aromatic cages may serve as a σ‐conjugated bridge between π‐substituents cannot be excluded.

For example, optically induced charge transfer was described in donor‐acceptor‐substituted carborane derivatives,^[^ ^17^ ^]^ moreover, several studies have described the delocalization of spin density in carborane‐based radical systems.^[^ ^18^, ^19^, ^20^, ^21^, ^22^ ^]^ On top of that, full electron delocalization across the carborane cages was reported (**IV**, Figure 1).^[^ ^21^, ^22^ ^]^ These observations were in contrast with previous studies where the local nature of the conjugation with the carborane cluster was highlighted. For example, there is only weak electronic communication between metal centers separated by *p*‐carboranes (**V**, Figure 1).^[^ ^23^, ^24^, ^25^, ^26^ ^]^ Similar phenomena were observed in the case of aryl‐substituted *meta‐* and *para*‐carboranes.^[^ ^27^, ^28^, ^29^, ^30^ ^]^ Very recently, the local nature of the interaction between the substituents and carborane cluster was further bolstered by boron‐substituted *ortho*‐carborane derivatives. In case of anionic π‐electron donor substituents, the local B─B bonds elongate and not the antipodal C─C bond, which means the ϭ* orbital of the B─B bonds populated and not the ϭ* C─C bond,^[^ ^31^ ^]^ which is usually more plastic, as it was demonstrated by Schleyer and Teixidor.^[^ ^32^ ^]^ Even though numerous studies deal with the effect of conjugation in the case of carborane‐based systems, there are no attempts to determine its overall strength in these systems and compare it with reference systems. In this study, our aim is to provide a comprehensive study and find out whether full electron delocalization can be achieved or the obtained results can be attributed to the effect of the well‐known hyperconjugation.

## 2 Results and Discussion

**Figure 2:**
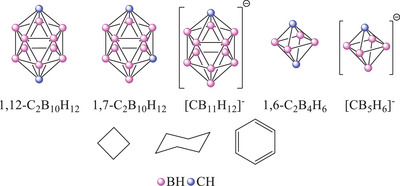
Investigated ϭ‐ and π‐systems.

In order to better understand the interaction of the substituents with and through the 3D unit, we have selected 3D aromatic carboranes, where the carbon atoms were separated by at least one BH vertex (Figure 2). Therefore, *meta*‐carborane (1,7‐dicarba‐*closo*‐dodecaborane, 1,7‐ C_2_B_10_H_12_), *para*‐carborane (1,12‐dicarba‐*closo*‐dodecaborane, 1,12‐C_2_B_10_H_12_) 1,6‐dicarba‐*closo*‐hexaborane(6) (1,6‐ C_2_B_4_H_6_), monocarba‐*closo*‐dodecaborate anion ([B_11_CH_11_]^−^) and monocarba‐*closo*‐hexaborate anion ([B_5_CH_6_]^−^), and as a reference, we have used the purely ϭ‐bonded cyclohexane, cyclobutane, π‐conjugated systems such as benzene, ethene, and acetylene. Various π‐donor substituents such as ─NH_2_, ─OH, ─SH, ─O^−^, ─S^−^, ─Ph, ─CH═CH_2_ (labeled by X) were considered to increase the possibility of conjugation through the carborane moiety. Since full electron delocalization was reported in the case of boranyl and phenyl substituted *para*‐carborane derivatives after reduction, we have included the corresponding ─BH_2_ and ─Ph substituted radical derivatives.

**Figure 3:**
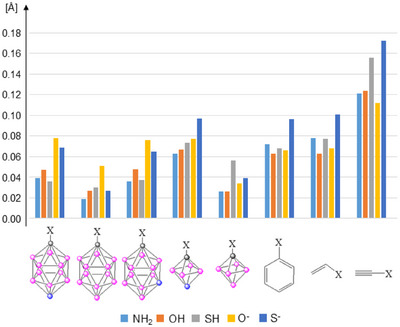
Bond shortening [Å], relative to the corresponding cyclohexane derivatives.

As a first step, the obtained optimized geometries of the mono carbon substituted derivatives (at B3LYP/6–311+G**) were investigated, with a special emphasis on the C─X bond length, which can be a sign of the conjugation. As a reference, the C─X bonds of the corresponding cyclohexane derivatives were used (Figure 3). As expected, the shortest C─X bond lengths were observed for phenyl, vinyl, and ethynyl substituents, where strong π–π conjugation is present. In contrast*, p*‐carborane‐based systems exhibited slightly longer bond lengths than the π‐conjugated counterparts, yet shorter than those of the cyclohexane derivatives. It indicates a certain level of conjugation, aligning with previous insights into the notable hyperconjugation between π‐systems and the 3D cluster framework. In anionic clusters, however, this interaction tends to be less pronounced, likely because electron donation is directed toward an already negatively charged unit. Interestingly, the bond lengths in the case of 1,6‐C_2_B_4_H_6_ are very similar to the benzene derivatives, which indicates a stronger interaction in this case compared to the *p*‐carborane system.

As a next step, the torsion of the carborane cluster is worth considering. Investigating the change of different bonds of the cluster framework (Table S1–S8 in the Supporting Information), it can be established that C─B bonds, in which the X substituents connected to the carbon atom elongate significantly in the case of carborane‐based systems, while other bonds do not change notably, indicating again the local nature of the interaction.

In order to quantify the stabilization resulting in the interaction between the substituents and the carborane framework, second‐order perturbation theory analysis on the NBO basis was performed. The obtained π–σ* stabilization interactions span a wide range (Table S10 in the Supporting Information) in the case of the carborane‐based system, strongly depending on the π‐donor ability of the substituents. The interaction is the strongest in the case of anionic ─O^−^ substituents, and it is in full agreement with the previous observations.^[^ ^32^, ^33^ ^]^ Another remarkable observation is that the donation usually occurs into the antibonding orbitals of the neighboring C─B cluster bonds, and we did not observe contributions from bonds distant from the substituted carbon atom (in contrast to benzene derivatives), which is in full agreement with the local nature of the geometric distortion as well. Atomic charge values computed based on Atoms in Molecules (AIM) theory further corroborate this statement, the computed charges of the vertices do not change far from the connected substituents as it was shown in Table S11–S23 in the Supporting Information.

**Figure 4:**
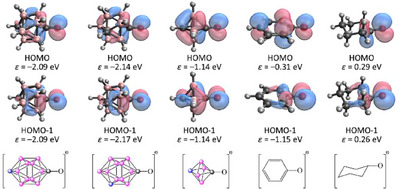
Kohn‐Sham HOMO and HOMO‐1 orbitals and their energies in eV at B3LYP/6–311 + G** level of theory (isovalue: 0.100 Å^−3^).

These results clearly demonstrated that the effect of electronic excess attached as a substituent to the carbon atom is almost perfectly buffered by the adjacent B─C bonds in the carborane clusters. This statement is further supported by an analysis of the molecular orbitals. Since the effect is most significant with the highest donating ability ─O^−^ substituents, we are focusing on this specific case (Figure 4). For the phenolate anion, the HOMO is delocalized across the aromatic ring, with the carbon atom at the *para* position making a notable contribution, despite being the furthest ring atom from the bonded oxygen. The HOMO–1 is primarily localized on the oxygen atom, only shows a slight interaction with the σ‐system of the ring instead of the π‐system due to symmetry. Notably, the electron density of the orbital diminishes with increasing distance from the oxygen atom. In contrast, for the cyclohexanolate anion, the HOMO is mainly localized on the oxygen atom. Interestingly, the HOMO–1 orbital closely resembles that of the phenolate anion. In *para*‐carborane‐based systems, the HOMO and HOMO–1 can be considered nearly degenerate due to the higher *C*_5*v*_ symmetry compared to the *C*_2*v*_ phenolate anion. However, the resemblance of these orbitals to the HOMO–1 of phenolate and cyclohexanolate anion is striking and cannot be overlooked. Similarly, the lone pairs on the oxygen atoms contribute significantly, along with the σ‐framework of the carborane cluster. Without a doubt, the delocalization of these orbitals is more pronounced than in the case of cyclohexanolate, although the overall character of the orbitals does not differ drastically. In case of 1,6‐C_2_B_4_H_6_, the carbon atom in position 6 has a significant contribution to the HOMO, meanwhile in case of *para*‐carborane the carbon atom in position 12 has only a very tiny contribution to these orbitals. This difference provided additional support for the local nature of the delocalization in the icosahedral clusters.

**Figure 5:**
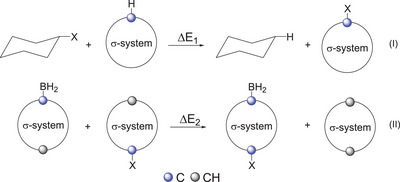
Isodesmic reactions to evaluate the conjugation ability of the investigated systems.

To quantify the interaction, isodesmic reactions were examined (Figure 5). We used the chair conformer of the cyclohexane (substituents are in axial positions) as a reference, since it has a well‐defined purely σ‐bonded structure. For neutral, strong π‐donor substituents like ─NH_2_, ─OH, ─SH, the isodesmic reaction I (see Figure 5) is endothermic for carborane‐based systems, suggesting a lack of stabilization compared to the corresponding cyclohexane derivatives (Table 1). In the case of π‐electron acceptor ─BH_2_ substituent, strong interaction was proposed in case of *para*‐carborane based on the in‐phase interaction of the empty acceptor orbital of the BH_2_ unit and the carbon atom 2p_z_ orbital of the cage.^[^ ^23^ ^]^ The isodesmic reaction energy of −2.5 kcal/mol verified the existence of the interaction, however it is relatively weak.

**Table 1:** Δ*E*_1_ Isodesmic reaction energies in kcal/mol.

X	1,12‐C_2_B_10_H_12_	[CB_11_H_11_]^−^	1,7‐C_2_B_10_H_12_	1,6‐C_2_B_4_H_6_	[CB_5_H_6_]^−^	benzene	cyclo‐C_4_H_8_
─BH_2_	−2.5	−4.8	−3.1	−6.1	−12.5	−8.7	−1.1
─CH═CH_2_	2.8	2.1	2.9	−1.2	−4.0	−3.3	−1.8
─Ph	3.6	2.1	3.4	−2.8	−7.1	−3.4	−1.6
─NH_2_	3.8	4.1	3.7	0.2	2.0	−4.9	−0.1
─OH	7.6	5.8	7.5	4.8	3.5	−2.2	0.7
─SH	7.5	3.9	7.7	3.1	−3.4	0.8	0.2
─O^−^	−36.1	−^[a]^	−37.8	−30.7	−^[a]^	−30.1	−4.8
─S^−^	−16.5	−^[a]^	−18.8	−15.8	–^[a]^	−13.9	−0.3
─O^−^[NMe_4_]^+^	−15.9	9.1	−16.4	−15.6	15.9	−16.4	−3.3
─S^−^[NMe_4_]^+^	−6.5	10.4	−8.5	−9.8	7.8	−9.5	−2.1
─BH_2_^−^	−23.7	–^[a]^	−26.7	−20.8	–^[a]^	−23.9	−0.9
─Ph^−^	−14.0	−^[a]^	−19.8	−14.1	–^[a]^	−21.3	−1.6

^[a]^ Due to the charge separations between the negatively charged cluster and the substituent, these reactions do not deliver proper results.

On the other hand, the interaction becomes significantly stronger in anionic clusters, especially in the case of X═O^−^ substituents (we have computed with noncoordinating [NMe_4_]^+^ cation as well to handle the problem of charge separation in the isodesmic reaction). The donation toward the cluster is much stronger in case of neutral clusters; the stabilization is comparable (−16.4) – (−15.6) kcal/mol for ─O[NMe_4_]^+^ to the corresponding benzene derivatives (−16.4 kcal/mol ─O[NMe_4_]^+^) which is in full agreement with the fact that the negative charge can be stabilized through negative hyperconjugation. For comparison, we have investigated the corresponding *ortho*‐carborane derivatives (Table S24 in the Supporting Information) as well, where the effect of the population of the antibonding orbitals results in more significant bond‐elongations,^[^ ^31^, ^32^ ^]^ indeed the interaction is stronger. In the case of anionic clusters, the less exothermic reactions verified our hypothesis that negative hyperconjugation is less effective because donating electron density to an already negatively charged cluster is inherently less favorable; accordingly, the isodesmic reaction energy values vary between −12.5 and 15.9 kcal/mol. These results demonstrate that strong interaction can be only expected in the case of anionic substituents X═ ─O^−^, ─S^−^, ─BH_2_^−^, and ─Ph^−^ in agreement with the bond elongations observed previously. On the other hand, this reaction does not deliver any information about the conjugation across the carborane clusters. Therefore, as a next step, donor–acceptor systems were investigated (Figure 5, isodesmic reaction II).

This reaction is suitable for judging the communication between the two substituents connected to the cluster. The calculated reaction energies (Table 2) indicate that in the case of neutral π‐donor substituents only weak interaction can be expected, even in the case of benzene derivatives. For anionic ─O^−^ and ─S^−^ substituents, the reaction energies become more exothermic; however, they remain below 5 kcal/mol in the case of icosahedral carboranes. In contrast, the isodesmic reaction becomes more exothermic for the 1,6‐B_4_C_2_H_6_ system, in full agreement with the higher conjugation ability proposed by the analysis of the orbitals, but still remains notably lower than those of the corresponding benzene derivatives. Interestingly, in the case of cyclobutane derivatives, −6.9 and −7.6 kcal/mol were computed, indicating a much stronger interaction through the two C─C σ‐bonds in the case of cyclohexane derivatives, which aligns well with the general understanding that conjugation through the σ‐system strongly depends on the distance. The difference between the systems based on *para*‐carborane and 1,6‐B_4_C_2_H_6_ can be explained by the relative distance of the donor and the acceptor units and the fact that the electronic effect of the substituents cannot be buffered in case of the smaller carborane cluster, as only one “boron layer” separates the two parts of the system. In general, *para*‐carborane systems exhibit more exothermic reaction by a few kcal/mol than the cyclohexane derivatives, similar differences can be seen (0.0–3.3 kcal/mol) between the systems based on 1,6‐B_4_C_2_H_6_ and cyclobutane. In view of these data, it can be established that the carborane systems provide certain extra conjugation, but it results only in a few kcal/mol of stabilization. At first glance, it appears that in carborane‐based systems, the extent of possible conjugation strongly depends on the spatial distance. However, data obtained for the *meta*‐carborane derivatives, where only two σ‐bonds separate the donor and acceptor units, suggest that interaction strength cannot be attributed solely to distance. In this case, lower values were obtained than the corresponding 1,6‐B_4_C_2_H_6_ and even lower than for cyclobutane derivatives. The possible explanation of this contradiction can be attributed to the symmetry of the orbitals and the orientation of the substituents (Figure 4). In the case of *p*‐carborane, the higher symmetry (*C*_5v_ compared to *C*_s_ in *m*‐carborane) of the system allows stronger conjugation ability.

**Table 2:** Δ*E*_2_ Isodesmic reaction energies in kcal/mol.

X	*p*‐B_10_C_2_H_12_	*m*‐B_10_C_2_H_12_	1,6‐B_4_C_2_H_6_	benzene	cyclobutane	cyclohexane
─CH═CH_2_	−0.2	0.0	−0.7	−0.6	0.2	0.1
─NH_2_	−0.7	0.0	−3.2	−3.6	−2.4	0.2
─SH	−0.5	−0.2	−1.9	−1.4	−0.7	0.1
─OH	−0.5	0.0	−2.3	−2.2	−0.6	0.1
─O^−^	−3.5	−2.3	−10.2	−18.4	−6.9	−1.5
─S^−^	−3.3	−1.6	−7.6	−13.5	−7.6	−1.3
─BH_2_^−^	−13.8	−9.0	−18.1	−21.3	−15.8	−11.2

In order to further support our statements obtained from the isodesmic reaction energies, combined ETS‐NOCV method was applied to estimate the interaction energy between the substituents and the σ‐system. Obviously, the main contribution in all cases is the σ‐bond between the side and the center parts, but third Natural Orbitals for Chemical Valence (NOCV) pairs can be assigned to the π‐π interaction through the σ‐system (see Figure S1 in the Supporting Information). It can be seen that the strength of this energy follows the same trend, which could be observed in the case of the isodesmic reactions (aromatic ring > 1,6‐B_4_C_2_H_6_ > *para*‐carborane ≈ *meta*‐carborane > cyclohexane). However, this method suggests a similar conjugation ability of *para*‐ and *meta*‐carborane isomers.

**Figure 6:**
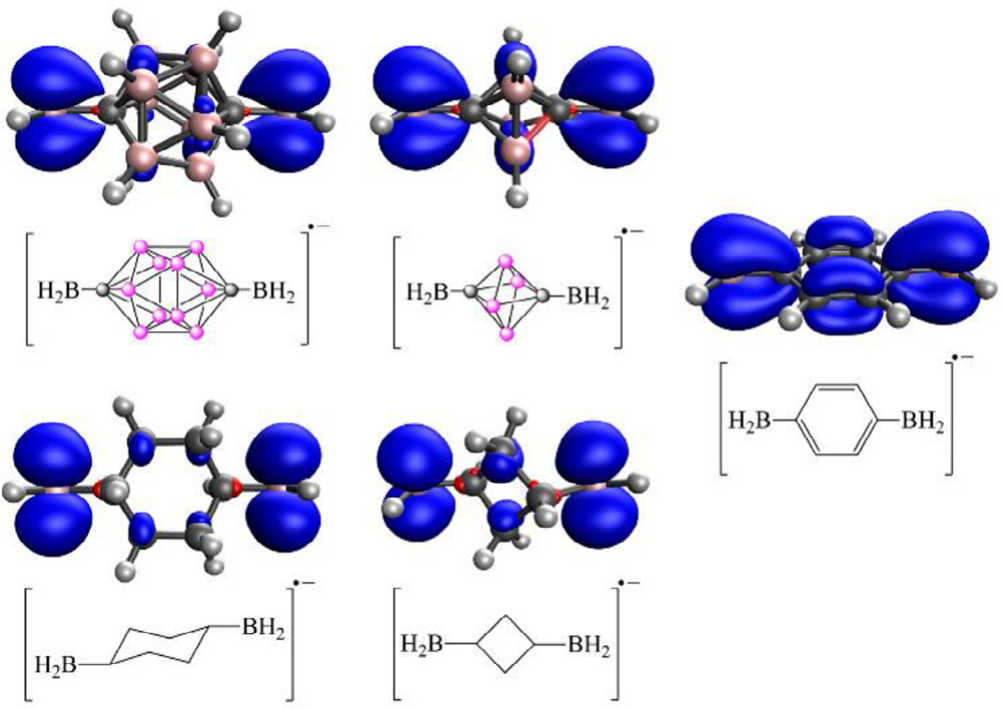
Computed spin densities of boranyl substituted radical anions (at B3LYP/6–311+G** and with the isovalue of 0.002).

Finally, systems containing an unpaired electron were examined. The donation ability of the ─BH_2_^−^ group was investigated as full‐electron delocalization was reported in this case.^[^ ^22^ ^]^ Indeed, the isodesmic reaction is the most exothermic (−13.8 kcal/mol) in this case, indicating significant delocalization of the radical center, in full agreement with the reported data. This statement can be extended to phenyl and amino‐substituted derivatives as well (Table S25, 26 in the Supporting Information). On the other hand, very similar isodesmic reaction energies were obtained for cyclobutane (−15.8 kcal/mol) and cyclohexane derivatives (−11.2 kcal/mol). It is important to note that the delocalization of the spin density through saturated hydrocarbons is a well‐established and known phenomenon.^[^ ^33^, ^34^, ^35^ ^]^ These results were further bolstered by the computed spin densities (Figure 6). In both *para*‐carborane and cyclohexane derivatives, the two ─BH_2_ units contribute predominantly to the spin density, while the carborane cluster and cyclohexane units also show minor, but still observable contributions. A notable difference between carborane‐ and cyclohexane‐based systems is that the spin density localized mainly on the p_z_ orbital of the boranyl boron atom is slightly polarized toward the carbon atom of the cluster in the case of carborane‐based systems. In view of these data, the delocalization of the spin density is not a unique property of the carborane cluster; simple σ‐conjugated systems exhibit very similar delocalization properties.

## 3 Conclusion

In our study, the conjugation ability of the substituents with and through the carborane clusters was deciphered. The interaction between the substituents and the carborane cage is primarily mediated through negative hyperconjugation. Owing to the inherently localized nature of hyperconjugation, electronic communication through the carborane framework remains limited, and in general, this effect is only slightly stronger than that of the corresponding cycloalkanes. In the presence of anionic substituents, enhanced negative hyperconjugation increases the efficiency of electronic interaction through the cluster. Significant delocalization can be observed in radical‐based systems in agreement with previous findings, but this phenomenon is also evident in simple alkyl chains, indicating that such behavior is not unique to carborane clusters. Importantly, the extent of conjugation is strongly influenced by the size and symmetry of the cluster. Notably, the octahedral 1,6‐dicarba‐*closo*‐hexaborane(6) exhibits a conjugation capability comparable to that of π‐aromatic benzene.

### 3.1 Computational Details

All calculations were carried out with Gaussian16 quantum chemistry package.^[^ ^36^ ^]^ Geometry optimizations in succession with frequency calculations were performed at the B3LYP/6–311+G** level of theory (for open‐shell systems, the corresponding unrestricted formalism was employed), which was widely used for similar systems.^[^ ^12^, ^37^, ^38^ ^]^ This method was validated for related systems by localized coupled‐cluster (CC) method at the LNO‐CCSD(T)/6–311 + G** level of theory.^[^ ^12^, ^39^ ^]^ Extended Transition State (ETS) scheme^[^ ^40^ ^]^ was applied to decompose the interaction energy between the substituents and the σ‐system. In the investigated double‐substituted systems, substituents were taken as doublet fractions, and the cluster was chosen as triplet to estimate covalent bond interaction energies, therefore three fragments (two substituents and the σ‐system) were defined. This method is often combined with the NOCV^[^ ^41^ ^]^ approach to estimate the contribution of different donor‐acceptor orbital pairs between the fragments to the total orbital interaction energy. For visualization of the molecules. The interaction energy was decomposed according to the ETS scheme with the sobEDA method^[^ ^42^ ^]^ implemented into the Multiwfn package,^[^ ^43^ ^]^ which program was able to compute NOCV analysis as well. Atomic charges computed based on AIM theory and were computed by the Multiwfn program.^[^ ^43^ ^]^ For NBO calculations, NBO 7.0^[^ ^44^ ^]^ was used. Kohn‐Sham molecular orbitals and spin densities, the Multiwfn^[^ ^43^ ^]^ and IQmol^[^ ^45^ ^]^ programs were used.

## Supporting Information

Supporting Information (including XYZ coordinates and total energies of the computed systems) is available from the Wiley Online Library.

## Conflict of Interest

The authors declare no conflict of interest.

## Supporting information



Supporting Information

Supporting Information

## Data Availability

The data that support the findings of this study are available in the supplementary material of this article.
