# Supplementary material for: Reassessing the Possibility of π–σ–π Full Electron Delocalization Through 3D Aromatic Carboranes
Source: Chemistry. 2025 Jun 27;31(41):e202501806. doi: 10.1002/chem.202501806 (PMC12284621; doi:10.1002/chem.202501806)
Supplement: Supplementary file 1 — Supporting Information [file CHEM-31-e202501806-s002.pdf]

Chemistry — A European Journal Supporting Information

Reassessing the Possibility of π–σ–π Full Electron Delocalization through 3D Aromatic Carboranes

Yanhong Gao, Balázs Szathmári, Dániel Buzsáki, Zsolt Kelemen

Table of Contents

Table S1. Bond lengths of substituted 1,12-$C_2B_{10}H_{12}$ 2

Table S2. Bond length differences between substituted 1,12-$C_2B_{10}H_{12}$ and 1,12-$C_2B_{10}H_{12}$ 3

Table S3. Bond lengths of substituted 1,6-$C_2B_4H_6$ 3

Table S4. Bond length differences between substituted 1,6-$C_2B_4H_6$ and 1,6-$C_2B_4H_6$ .. 3

Table S5. Bond lengths of substituted benzene 4

Table S6. Bond length differences between substituted benzene and benzene 4

Table S7. Bond lengths of substituted cyclohexane 5

Table S8. Bond length differences between substituted cyclohexane and cyclohexane6

Table S9. Second order perturbation theory analysis on the NBO basis: Donor: Lone pairs of the substituents Acceptor: 2 and 3 center bonds of the σ-system as it was defined in NBO 7.0 program 6

Table S10. AIM Charges of each unit of substituted 1,12-$C_2B_{10}H_{12}$ 6

Table S11. AIM Charge differences of each unit of substituted 1,12-$C_2B_{10}H_{12}$ 7

Table S12. AIM Charges of each unit of substituted 1,7-$C_2B_{10}H_{12}$ 7

Table S13. AIM Charge differences of each unit of substituted 1,7-$C_2B_{10}H_{12}$ 8

Table S14. Charges of each unit of substituted $CB_{11}H_{12}^-$ 8

Table S15. Charge differences of each unit of substituted $CB_{11}H_{12}^-$ 8

Table S16. AIM charges of each unit of substituted 1,6-$C_2B_4H_6$ 9

Table S17. AIM Charge differences of each unit of substituted 1,6-$C_2B_4H_6$ 9

Table S18. AIM Charges of each unit of substituted $CB_5H_6^{-1}$ 9

Table S19. AIM Charge differences of each unit of substituted $CB_5H_6^-$ 10

Table S20. AIM Charges of each unit of substituted benzene 10

Table S21. AIM Charge differences of each unit of substituted benzene 10

Table S22. AIM Charges of each unit of substituted cyclohexane 11

Table S23. AIM Charge differences of each unit of substituted cyclohexane 11

Table S24. Isodesmic reaction to investigate systems with phenyl substituents 13

Table S25. Isodesmic reaction to investigate systems with two phenyl substituents .. 13

Table S26. Isodesmic reaction to investigate systems with two amino substituents..13

Figure S2. Spindensity of the radical cation derived from the 1,12-diamino-1,12-carborane (computed at B3LYP/6-311+G**, isovalue=0.002……………………….14

Table S1. Bond lengths of substituted 1,12-$C_2B_{10}H_{12}$

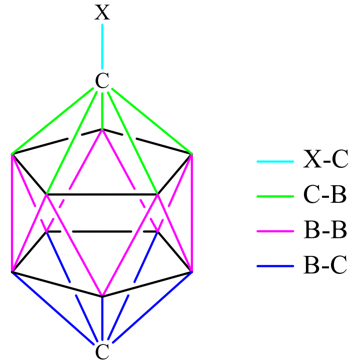

X	Bond type	Bond length [Å]					Average [Å]
O^-	O–C	1.257					1.257
	C–B	1.790	1.790	1.790	1.790	1.790	1.790
	B–B	1.764	1.764	1.764	1.764	1.764	1.764
		1.764	1.764	1.764	1.764	1.764	
	B–C	1.709	1.709	1.709	1.709	1.709	1.709
OH	HO–C	1.386					1.386
	C–B	1.716	1.715	1.728	1.707	1.727	1.719
	B–B	1.765	1.763	1.763	1.765	1.764	1.764
		1.764	1.759	1.770	1.770	1.759	
	B–C	1.704	1.708	1.704	1.709	1.708	1.707
BH_2	H_2B–C	1.565					1.565
	C–B	1.715	1.727	1.723	1.716	1.731	1.722
	B–B	1.768	1.763	1.768	1.772	1.760	1.761
		1.765	1.760	1.770	1.721	1.759	
	B–C	1.702	1.709	1.706	1.704	1.711	1.706
BH_2 radical	H_2B–C	1.580					1.580
	C–B	1.735	1.749	1.749	1.735	1.761	1.746
	B–B	1.772	1.777	1.760	1.760	1.777	1.768
		1.762	1.771	1.771	1.762	1.772	
	B–C	1.717	1.700	1.717	1.707	1.707	1.710

Table S2. Bond length differences between substituted 1,12-$C_2B_{10}H_{12}$ and non-substituted 1,12-$C_2B_{10}H_{12}$

	X=O^-	X=OH	X=BH_2	X=BH_2^-
C-B	0.083	0.012	0.015	0.039
B-B	-0.002	-0.002	-0.005	0.002
B-C	0.003	0.001	0.000	0.004

Table S3. Bond lengths of substituted 1,6-$C_2B_4H_6$

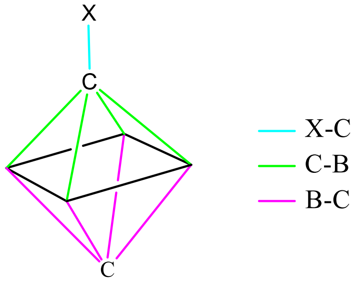

X	Bond type	Bond length [Å]				Average [Å]
O^-	O–C	1.258				1.258
	C–B	1.693	1.692	1.693	1.694	1.693
	B–C	1.621	1.621	1.621	1.622	1.621
OH	O–C	1.366				1.366
	C–B	1.649	1.609	1.649	1.614	1.630
	B–C	1.604	1.634	1.606	1.629	1.618
BH_2	H_2B–C	1.541				1.541
	C–B	1.618	1.663	1.618	1.663	1.641
	B–C	1.643	1.604	1.643	1.604	1.624
BH_2 radical	H_2B–C	1.562				1.562
	C–B	1.677	1.637	1.677	1.637	1.657
	B–C	1.612	1.648	1.612	1.648	1.630

Table S4. Bond length differences between substituted 1,6-$C_2B_4H_6$ and non-substituted 1,6-$C_2B_4H_6$

	X=O^-	X=OH	X=BH_2	X=BH_2^-
C-B	0.069	0.006	0.017	0.033
B-C	-0.002	-0.005	0.000	0.007

Table S5. Bond lengths of substituted benzene

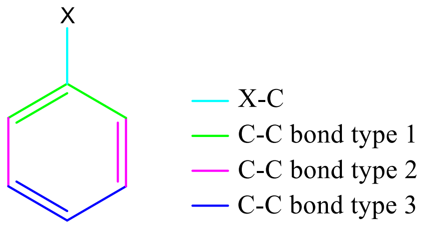

X	Bond type	Bond length [Å]		Average [Å]
O^-	O–C	1.269		1.269
	C–C Bond type 1	1.447	1.447	1.447
	C–C Bond type 2	1.387	1.388	1.388
	C–C Bond type 3	1.404	1.404	1.404
OH	HO–C	1.370		1.370
	C–C Bond type 1	1.396	1.396	1.396
	C–C Bond type 2	1.391	1.394	1.393
	C–C Bond type 3	1.393	1.396	1.395
BH_2	H_2B–C	1.536		1.536
	C–C Bond type 1	1.412	1.412	1.412
	C–C Bond type 2	1.391	1.391	1.391
	C–C Bond type 3	1.396	1.396	1.396
BH_2 radical	H_2B–C	1.519		1.519
	C–C Bond type 1	1.438	1.438	1.438
	C–C Bond type 2	1.384	1.385	1.385
	C–C Bond type 3	1.410	1.409	1.410

Table S6. Bond length differences between substituted benzene and non-substituted benzene

	X=O^-	X=OH	X=BH_2	X=BH_2^-
C-C Bond type 1	0.052	0.001	0.017	0.043

C-C Bond type 2	-0.008	-0.002	-0.004	-0.011
C-C Bond type 3	0.009	-0.001	0.001	0.015

Table S7. Bond lengths of substituted cyclohexane

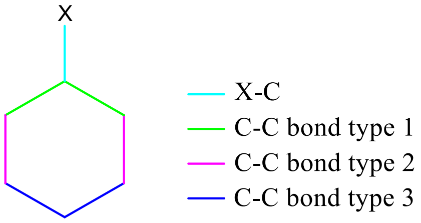

X	Bond type	Bond length [Å]		Average [Å]
O^-	O–C	1.335		1.335
	C–C Bond type 1	1.569	1.569	1.569
	C–C Bond type 2	1.538	1.538	1.538
	C–C Bond type 3	1.537	1.537	1.537
OH	HO–C	1.433		1.433
	C–C Bond type 1	1.532	1.526	1.529
	C–C Bond type 2	1.537	1.536	1.537
	C–C Bond type 3	1.535	1.536	1.536
BH_2	H_2B–C	1.554		1.554
	C–C Bond type 1	1.545	1.545	1.545
	C–C Bond type 2	1.536	1.536	1.536
	C–C Bond type 3	1.535	1.535	1.535
BH_2 radical	H_2B–C	1.612		1.612
	C–C Bond type 1	1.543	1.543	1.543
	C–C Bond type 2	1.539	1.539	1.539
	C–C Bond type 3	1.536	1.536	1.536

Table S8. Bond length differences between substituted cyclohexane and non-substituted cyclohexane

	O^-	OH	BH_2	BH_2 radical
C-C Bond type 1	0.033	-0.007	0.009	0.007
C-C Bond type 2	0.002	0.000	0.000	0.003
C-C Bond type 3	0.001	-0.001	-0.001	0.000

Table S9. Second order perturbation theory analysis on the NBO basis: Donor: Lone pairs of the substituents Acceptor: 2 and 3 center bonds of the σ-system as it was defined in NBO 7.0 program

	NH_2	SH	OH	O^-	S^-	BH_2
1,12-$C_2B_{10}H_{12}$	7.8	6.7	14.0	150.8	20.8	4.9
1,7-$C_2B_{10}H_{12}$	7.1	8.1	12.8	44.0	21.6	5.1
Benzene	30.5	22.1	37.5	40.6	50.4	7.8
1,6-$C_2B_4H_6$	13.2	10.4	19.0	73.6	27.4	6.6
Cyclohexane	9.5	7.5	15.5	59.6	18.1	6.6
$CB_5H_6^{-1}$	8.5	8.7	14.5	56.9	17.9	6.8
$CB_{11}H_{12}^{-1}$	5.5	5.7	10.5	57.1	15.2	4.4

Table S10. AIM Charges of each unit of substituted 1,12-$C_2B_{10}H_{12}$

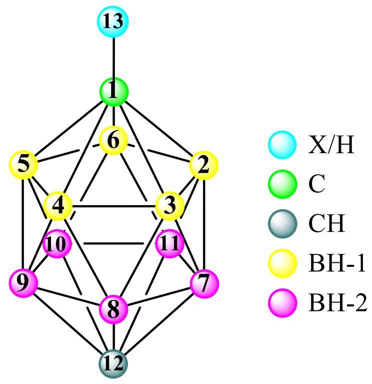

	NH_2	SH	OH	O^-	S^-	BH_2	1,12-$C_2B_{10}H_{12}$
X/H	-0.24	0.11	-0.47	-1.17	-0.45	0.68	0.09
C	-1.24	-1.68	-1.09	-0.18	-1.52	-2.18	-1.70
CH	-1.61	-1.61	-1.61	-1.60	-1.61	-1.61	-1.61
BH-1	0.31	0.30	0.31	0.17	0.28	0.30	0.32

BH-2	0.33	0.33	0.32	0.22	0.23	0.32	0.32

Table S11. AIM Charge differences of each unit of substituted 1,12-$C_2B_{10}H_{12}$

	NH_2	SH	OH	O-	S-	BH_2
CH	-0.47	-0.03	-0.62	-1.52	-0.19	0.47
C	0.01	0.01	0.01	0.15	0.03	0.02
BH-1	-0.01	-0.01	0.00	0.10	0.09	0.00
BH-2	-0.01	0.00	0.00	-0.01	-0.01	0.00

Table S12. AIM Charges of each unit of substituted 1,7-$C_2B_{10}H_{12}$

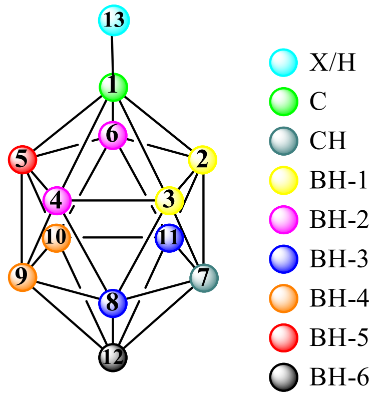

	NH_2	SH	OH	O^-	S^-	BH_2	1,7-$C_2B_{10}H_{12}$
X/H	-0.23	0.12	-0.47	-1.17	-0.45	0.68	0.15
C	-1.27	-1.70	-1.10	-0.20	-1.55	-2.20	-1.39
CH	-1.62	-1.63	-1.63	-1.65	-1.65	-1.62	-1.65
BH-1	0.68	0.68	0.71	1.63	0.61	0.67	0.60
BH-2	0.26	0.30	0.30	0.22	0.24	0.30	0.33
BH-3	0.30	0.27	0.26	0.15	0.25	0.27	0.24
BH-4	0.07	0.07	0.08	-0.02	-0.01	0.07	0.08
BH-5	0.22	0.28	0.27	0.19	0.21	0.28	0.31
BH-6	0.28	0.26	0.24	0.15	0.24	0.24	0.20

Table S13. AIM Charge differences of each unit of substituted 1,7-$C_2B_{10}H_{12}$

	NH_2	SH	OH	O^-	S^-	BH_2
C	-0.13	0.30	-0.30	-1.20	0.15	0.80
CH	-0.03	-0.03	-0.02	0.00	-0.01	-0.03
BH-1	-0.09	-0.09	-0.11	0.13	-0.01	-0.07
BH-2	0.07	0.03	0.02	0.10	0.09	0.03
BH-3	-0.06	-0.03	-0.02	0.09	-0.01	-0.03
BH-4	0.01	0.01	0.00	0.10	0.09	0.01
BH-5	0.09	0.02	0.04	0.11	0.09	0.02
BH-6	-0.08	-0.06	-0.04	0.05	-0.04	-0.04

Table S14. Charges of each unit of substituted $CB_{11}H_{12}^-$

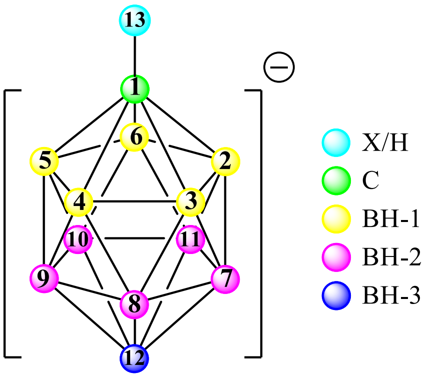

	NH_2	SH	OH	O^-	S^-	BH_2	$CB_{11}H_{12}^-$
X/H	-0.29	0.00	-0.51	-1.22	-0.63	0.63	0.04
C	-1.27	-1.64	-1.12	-0.33	-1.54	-2.17	-1.67
BH-1	0.13	0.18	0.16	0.07	0.18	0.17	0.19
BH-2	-0.04	-0.04	-0.04	-0.13	-0.12	-0.05	-0.05
BH-3	-0.07	-0.06	-0.07	-0.15	-0.13	-0.06	-0.07

Table S15. Charge differences of each unit of substituted $CB_{11}H_{12}^-$

	NH_2	SH	OH	O^-	S^-	BH_2
C	-0.40	-0.02	-0.54	-1.34	-0.13	0.51
BH-1	0.05	0.01	0.02	0.12	0.01	0.02
BH-2	-0.01	-0.01	-0.01	0.08	0.07	0.00

BH-3	0.00	-0.01	0.00	0.08	0.06	-0.01

Table S16. AIM charges of each unit of substituted 1,6-$C_2B_4H_6$

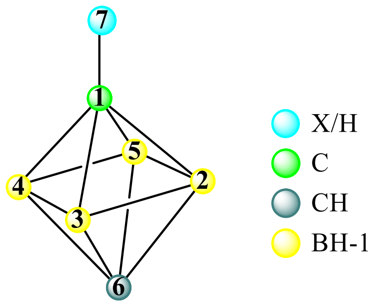

	NH_2	SH	OH	O^-	S^-	BH_2	1,6-$C_2B_4H_6$
X/H	-0.29	0.11	-0.51	-1.23	-0.48	0.68	0.07
C	-1.35	-1.85	-1.22	-0.36	-1.70	-2.35	-1.88
CH	-1.83	-1.83	-1.85	-1.82	-1.78	-1.80	-1.81
BH-1	0.86	0.90	0.90	0.60	0.74	0.86	0.90

Table S17. AIM Charge differences of each unit of substituted 1,6-$C_2B_4H_6$

	NH_2	SH	OH	O^-	S^-	BH_2
C	-0.53	-0.03	-0.66	-1.52	-0.18	0.47
CH	0.03	0.02	0.04	0.01	-0.02	-0.01
BH-1	0.05	0.00	0.01	0.30	0.16	0.05

Table S18. AIM Charges of each unit of substituted $CB_5H_6^{-1}$

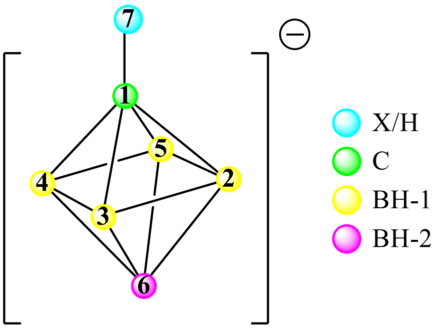

	NH_2	SH	OH	O^-	S^-	BH_2	$CB_5H_6^-$
X/H	-0.37	-0.08	-0.55	-1.27	-0.74	0.57	-0.01
C	-1.40	-1.77	-1.29	-0.63	-1.70	-2.30	-1.79
BH-1	0.18	0.22	0.22	0.04	0.16	0.19	0.22

BH-2	-0.05	-0.03	-0.04	-0.27	-0.21	-0.05	-0.07

Table S19. AIM Charge differences of each unit of substituted $CB_5H_6^-$

	NH_2	SH	OH	O^-	S^-	BH_2
C	-0.39	-0.02	-0.50	-1.16	-0.09	0.51
BH-1	0.04	0.00	0.00	0.18	0.06	0.02
BH-2	-0.02	-0.04	-0.03	0.20	0.14	-0.02

Table S20. AIM Charges of each unit of substituted benzene

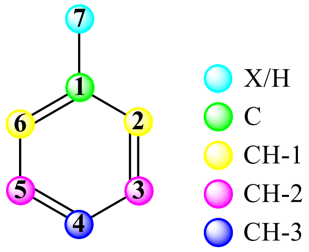

	NH_2	SH	OH	O^-	S^-	BH_2	Benzene
X/H	-0.33	0.06	-0.52	-1.23	-0.52	0.62	0.02
C	0.37	-0.13	0.48	0.83	-0.13	-0.64	-0.02
CH-1	-0.01	0.04	0.02	-0.11	-0.05	0.03	0.00
CH-2	0.00	0.00	0.03	-0.11	-0.07	-0.02	0.00
CH-3	-0.01	0.00	0.00	-0.14	-0.11	0.01	0.00

Table S21. AIM Charge differences of each unit of substituted benzene

	NH_2	SH	OH	O^-	S^-	BH_2
C	-0.39	0.11	-0.50	-0.85	0.11	0.62
CH-1	0.01	-0.04	-0.02	0.12	0.05	-0.03
CH-2	0.00	0.00	-0.03	0.11	0.07	0.02
CH-3	0.01	-0.01	0.00	0.14	0.10	-0.01

Table S22. AIM Charges of each unit of substituted cyclohexane

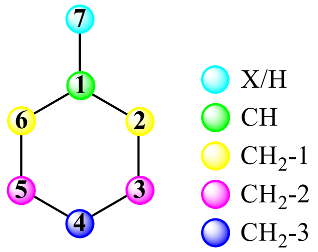

	NH_2	SH	OH	O^-	S^-	BH_2	Cyclohexane
X/H	-0.34	-0.07	-0.53	-1.26	-0.75	0.61	-0.02
CH	0.31	-0.03	0.46	0.62	-0.06	-0.63	0.02
CH_2-1	0.01	0.03	0.04	-0.08	-0.01	0.01	0.00
CH_2-2	0.00	0.01	0.01	-0.08	-0.06	0.00	0.00
CH_2-3	0.01	0.01	0.01	-0.06	-0.05	0.00	0.00

Table S23. AIM Charge differences of each unit of substituted cyclohexane

	NH_2	SH	OH	O^-	S^-	BH_2
X	0.32	0.05	0.50	1.24	0.73	-0.64
CH	-0.29	0.05	-0.44	-0.60	0.08	0.65
CH_2-1	-0.01	-0.03	-0.04	0.08	0.01	-0.01
CH_2-2	0.00	-0.01	-0.01	0.08	0.06	0.00
CH_2-3	-0.01	-0.01	-0.01	0.06	0.05	0.00

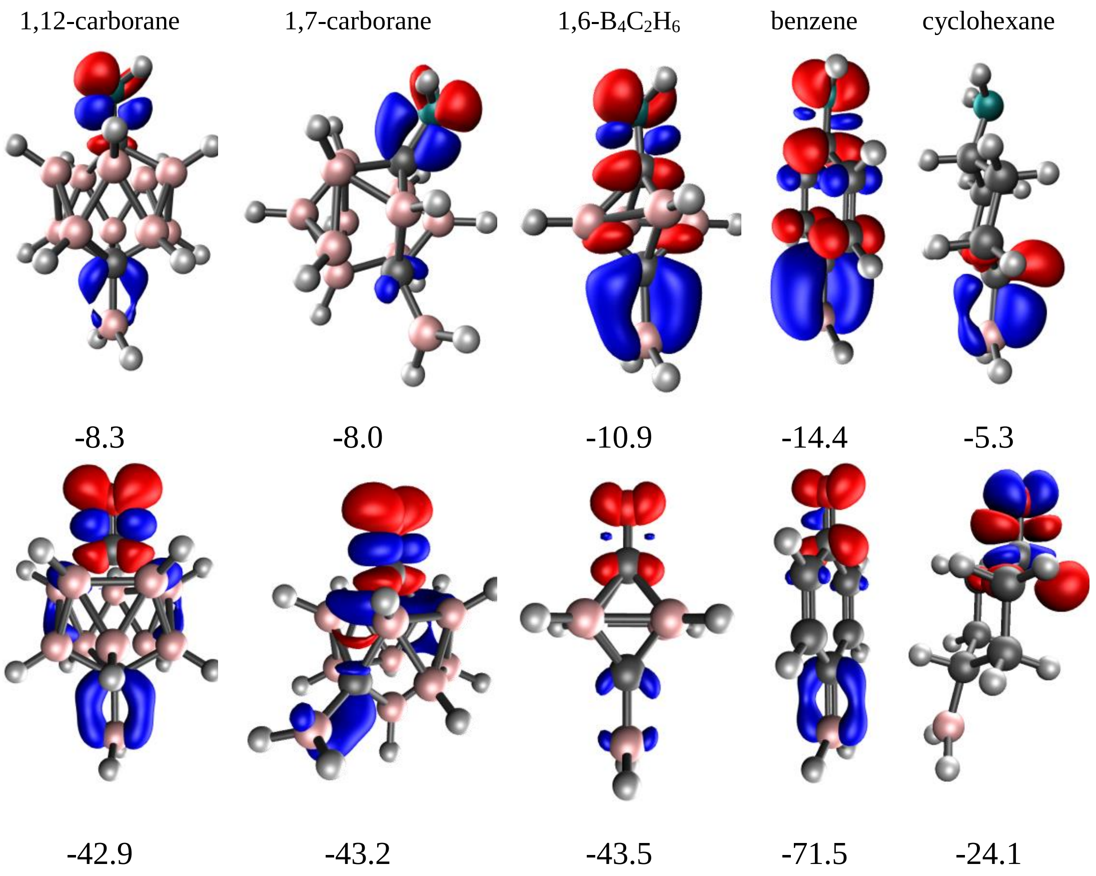

Figure S1. The third NOCV pairs of BH_2-NH_2 (top) and BH_2-O^- (bottom) substituted systems and their total pi-sigma-pi orbital interaction energies (in kcal/mol) It can be seen that the strength of this energy follows the same trend which could be observed in case of the isodesmic reactions (benzene > 1,6-B_4C_2 > 1,12-carborane ≈ 1,7-carborane > cyclohexane), although this method highlights a quite significant difference between carborane systems and cyclohexane. Moreover, the contribution of the acceptor orbital does not extend to the acceptor substituent in the case of cyclohexane, even though the direction of orbital relaxation changes in the BH_2-O^- substituted system, while the meta-carborane contains a much smaller orbital lobe at the acceptor side compared to its para counterpart. Both of these phenomena show diminished conjugation effects in these systems.

Table S24. Isodesmic reaction to investigate systems with phenyl substituents

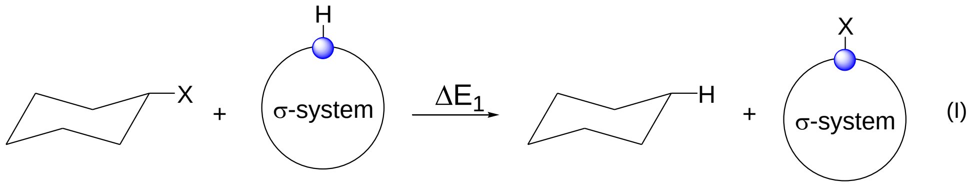

X	1,2-$C_2B_{10}H_{12}$	1,7-$C_2B_{10}H_{12}$	1,12-$C_2B_{10}H_{12}$
	ΔE [kcal/mol]		
–BH_2	-5.5	–3.1	–2.5
–CH=CH_2	1.8	2.9	2.8
–Ph	3.6	3.4	3.6
–NH_2	-0.4	3.7	3.8
–OH	5.4	7.5	7.6
–SH	9.4	7.7	7.5
–O-	-57.9	–37.8	–36.1
–S-	-29.0	–18.8	–16.5

Table S25. Isodesmic reaction to investigate systems with two phenyl substituents

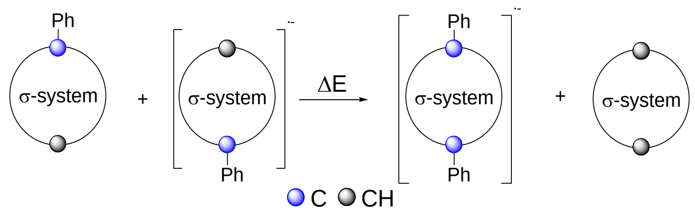

σ-system	ΔE [kcal/mol]
cyclohexane	-4.0
cyclobutane	-3.9
1,12-$C_2B_{10}H_{12}$	-7.7
1,7-$C_2B_{10}H_{12}$	-4.4
1,6-$C_2B_4H_6$	-11.2
benzene	-11.7

Table S26. Isodesmic reaction to investigate systems with two amino substituents

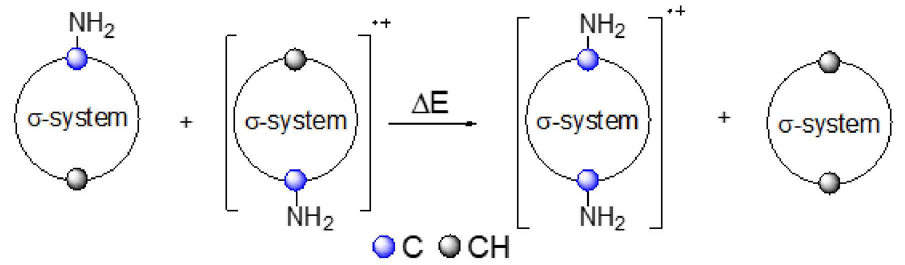

σ-system	ΔE [kcal/mol]
cyclohexane	-5.2
1,12-$C_2B_{10}H_{12}$	-14.1
1,7-$C_2B_{10}H_{12}$	-10.8
1,6-$C_2B_4H_6$	-19.9
benzene	-21.5

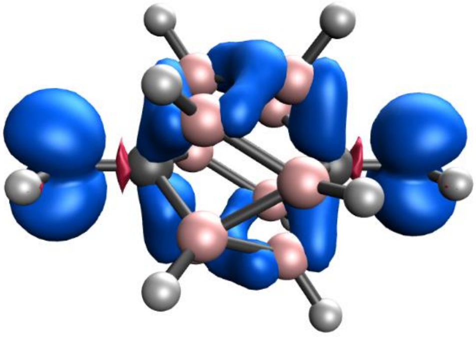

Figure S2. Spin density of the radical cation derived from the 1,12-diamino-1,12-$C_2B_{10}H_{12}$ (computed at B3LYP/6-311+G**, isovalue=0.002)
