# Supplementary material for: Reassessing the Possibility of π–σ–π Full Electron Delocalization Through 3D Aromatic Carboranes
Source: Chemistry. 2025 Jun 27;31(41):e202501806. doi: 10.1002/chem.202501806 (PMC12284621; doi:10.1002/chem.202501806)
Supplement: Supplementary file 2 — Supporting Information [file CHEM-31-e202501806-s001.pdf]

24			
***p*-carborane** scf done: -332.194254			
B	-0.002077	-0.013448	0.134328
H	0.014359	0.112365	1.306052
B	1.530793	-0.015820	-0.781642
B	1.256427	0.851256	-2.318338
C	0.427517	1.282295	-0.889762
B	-1.224120	0.854475	-0.836481
B	-1.203575	-0.910966	-0.798740
B	0.498504	-1.448697	-0.765367
C	-0.374922	-1.342085	-2.227040
H	-0.658990	-2.271269	-2.700250
B	1.276677	-0.914451	-2.280682
B	0.054827	-0.046420	-3.251423
B	-1.478127	-0.044471	-2.335292
B	-0.446397	1.389156	-2.352184
H	-2.010179	1.551389	-0.302421
H	2.554487	0.109446	-0.211423
H	0.038071	-0.172021	-4.423053
H	2.099513	1.546388	-2.759669
H	2.063168	-1.611154	-2.814354
H	-2.501961	-0.168996	-2.905433
H	-0.720792	2.437307	-2.815687
H	0.711583	2.211454	-0.416762
H	0.773331	-2.496760	-0.301811
H	-2.046719	-1.606086	-0.357439

26			
***p*-carborane X= BH_2** scf done: -357.629849			
C	1.783611	-0.000329	0.000758
B	1.008130	-1.337776	-0.719600
B	-0.482102	-1.509107	0.201322
B	-0.497072	-0.659326	-1.363303
B	-0.487011	1.106359	-1.043296
B	-0.486968	1.342864	0.714411
C	-1.302171	0.000513	-0.000831
B	-2.867740	0.000205	-0.000382
B	1.013898	1.504023	-0.202438
B	0.998451	0.274444	-1.494399
B	0.997806	0.658066	1.368382
B	-0.499642	-0.277881	1.489116
B	1.006965	-1.099752	1.045689
H	2.864489	-0.000561	0.001314
H	-1.103633	1.830725	-1.737707
H	-1.113665	-0.457314	2.480278
H	-1.106775	2.224745	1.193396
H	1.633141	-1.819545	1.741652
H	-1.103123	-2.501378	0.336434
H	1.619001	1.086710	2.275643
H	1.642287	2.491729	-0.333760
H	1.621467	0.449128	-2.480372
H	-1.112219	-1.093068	-2.273514
H	1.634418	-2.214195	-1.199727

H -3.454909 1.032580 -0.147004
H -3.453868 -1.032786 0.145131

26
***p*-carborane X= NH₂** scf done: -387.556767

C	0.000383	-0.000635	-0.030051
H	-0.001167	0.001693	1.050230
B	1.519319	-0.003529	-0.818001
B	0.469788	1.440386	-0.809490
B	-1.223440	0.893144	-0.814186
B	-0.466731	1.454113	-2.298708
C	0.003113	-0.001156	-3.126666
N	-0.006232	0.047621	-4.557562
B	0.468079	-1.443862	-0.797302
B	1.223861	-0.902688	-2.299511
B	1.219491	0.887664	-2.315956
B	-1.226850	-0.892915	-0.817941
B	-0.461088	-1.451857	-2.296329
B	-1.501161	0.003545	-2.320312
H	2.031709	-1.485793	-2.932197
H	-0.772496	2.398030	-2.931626
H	2.032765	1.468296	-2.941763
H	-2.039424	1.473485	-0.192614
H	-2.499854	-0.001776	-2.948110
H	0.788778	2.388555	-0.186393
H	2.521512	0.002139	-0.197838
H	0.777216	-2.394137	-0.172132
H	-0.772982	-2.396998	-2.930137
H	-2.044727	-1.473332	-0.198830
H	0.892629	-0.203673	-4.950275
H	-0.722525	-0.549754	-4.951751

25
***p*-carborane X= SH** scf done: -730.397656

C	-0.000883	-0.002608	-0.009858
H	-0.007631	-0.009731	1.070531
B	1.521301	-0.001668	-0.782424
B	0.473927	1.445170	-0.780735
B	-1.221375	0.896542	-0.786767
B	-0.459641	1.463394	-2.272584
C	0.017392	0.017096	-3.091229
S	0.084429	0.075346	-4.907581
B	0.470878	-1.440662	-0.792339
B	1.245361	-0.890017	-2.276636
B	1.238113	0.898257	-2.280297
B	-1.225516	-0.887109	-0.809570
B	-0.452331	-1.431064	-2.300137
B	-1.504978	0.021161	-2.296193
H	2.054375	-1.471033	-2.903443

H -0.760062 2.415501 -2.895703
H 2.051105 1.488103 -2.896431
H -2.035888 1.478428 -0.165043
H -2.499920 0.023395 -2.927436
H 0.788021 2.390491 -0.151408
H 2.518794 0.000187 -0.155257
H 0.769240 -2.398219 -0.173749
H -0.762376 -2.376056 -2.932453
H -2.041919 -1.480078 -0.200889
H -0.943612 -0.771669 -5.110720

25
***p*-carborane X= OH** scf done: -407.426134
C -0.000367 -0.001206 -0.036970
H 0.002432 0.000379 1.043334
B 1.516280 -0.004493 -0.823312
B 0.468791 1.441239 -0.823805
B -1.227371 0.893519 -0.809907
B -0.476599 1.448808 -2.307230
C -0.005711 -0.002810 -3.115150
O 0.034245 0.031013 -4.500403
B 0.467053 -1.444986 -0.811812
B 1.228373 -0.903430 -2.307394
B 1.220108 0.883946 -2.325982
B -1.231468 -0.891992 -0.817506
B -0.468405 -1.443380 -2.308913
B -1.519729 0.006154 -2.306833
H 2.026633 -1.487170 -2.946464
H -0.782524 2.389393 -2.945795
H 2.022620 1.465196 -2.961500
H -2.036379 1.479109 -0.184492
H -2.511398 -0.000159 -2.946538
H 0.787748 2.390081 -0.202262
H 2.517230 0.002496 -0.201732
H 0.770398 -2.397434 -0.187786
H -0.783402 -2.382636 -2.950901
H -2.042019 -1.480569 -0.196951
H -0.659733 -0.537907 -4.852048

24
***p*-carborane X= O^-** scf done: -406.886527
C 0.000250 0.000130 -0.024584
H -0.000831 0.000540 1.055547
B 1.513631 -0.001643 -0.820809
B 0.470224 1.437345 -0.821529
B -1.221462 0.889995 -0.823208
B -0.464219 1.441681 -2.317210
C 0.003348 -0.000734 -3.271282
O 0.004483 -0.000471 -4.528426

B 0.466701 -1.438195 -0.820580
B 1.226829 -0.892172 -2.315643
B 1.227917 0.888441 -2.316530
B -1.223656 -0.887515 -0.823203
B -0.466990 -1.440803 -2.316910
B -1.511275 0.001507 -2.319285
H 2.062005 -1.499433 -2.894294
H -0.781432 2.422990 -2.898027
H 2.064733 1.493945 -2.894712
H -2.030399 1.478115 -0.186847
H -2.543395 0.002190 -2.898881
H 0.780086 2.387753 -0.184384
H 2.512371 -0.002460 -0.182340
H 0.774058 -2.389257 -0.183162
H -0.786896 -2.421849 -2.896825
H -2.033536 -1.473780 -0.186387

24
***p*-carborane X= S^-** scf done: -729.866239
C 0.000412 -0.000396 -0.005517
H -0.000564 -0.000456 1.075272
B 1.512349 -0.001884 -0.799087
B 0.469688 1.436108 -0.799349
B -1.220461 0.888981 -0.800968
B -0.462739 1.437169 -2.301884
C 0.003156 0.000227 -3.179083
S 0.004709 0.000428 -4.953769
B 0.466358 -1.437503 -0.799765
B 1.223058 -0.889245 -2.301333
B 1.224659 0.886121 -2.301341
B -1.222472 -0.887133 -0.801571
B -0.465718 -1.435968 -2.302779
B -1.507140 0.001761 -2.303935
H 2.051127 -1.491401 -2.887309
H -0.777362 2.410695 -2.889159
H 2.054219 1.486848 -2.886726
H -2.031470 1.478658 -0.171486
H -2.530464 0.002834 -2.890880
H 0.780084 2.388907 -0.168834
H 2.514140 -0.003021 -0.168150
H 0.774500 -2.391246 -0.169551
H -0.782934 -2.408682 -2.890023
H -2.034586 -1.475478 -0.172282

34
***p*-carborane X= Ph** scf done: -563.294582
C -0.008784 -0.023181 -0.023487
B 0.007691 -0.003839 1.681423
B 1.517145 -0.021468 0.739423

B	2.076846	1.641653	0.570012
B	0.855556	2.548189	-0.361125
B	-0.458334	1.444997	-0.766670
B	1.230505	0.875142	-0.774036
B	-1.213643	0.902898	0.750407
B	-0.365473	1.668105	2.091152
C	0.887541	2.609791	1.364668
C	1.344267	3.898493	2.020648
B	-0.655488	2.570206	0.582462
B	1.323297	1.101000	2.091300
H	1.722783	0.506197	-1.779362
H	-1.080031	1.452494	-1.767953
H	2.199382	-0.982528	0.732808
H	-0.885626	1.996894	3.093514
H	-0.314686	-0.952515	2.302015
H	1.947325	1.092553	3.090464
H	-1.336946	3.531148	0.586062
H	-2.337863	0.549225	0.759417
H	-0.320651	-0.938531	-0.505727
H	3.205301	1.977777	0.563080
H	1.164517	3.492416	-0.993117
C	2.358397	4.667600	1.436865
C	2.786626	5.852656	2.029336
C	2.209819	6.293407	3.217875
C	1.200698	5.536317	3.806900
C	0.771798	4.350577	3.214634
H	2.819677	4.343144	0.513773
H	3.573514	6.430377	1.557766
H	2.542993	7.215937	3.679356
H	0.741500	5.865619	4.732124
H	-0.013194	3.778978	3.689803

34

***p*-carborane X= Ph**$^{\cdot-}$ scf done: -563.288821

C	0.000000	-0.000000	0.000000
C	0.000000	0.000000	1.432494
C	1.284295	0.000000	2.064251
C	2.454580	0.005973	1.329373
C	2.437361	0.012057	-0.083478
C	1.180529	0.006012	-0.721753
C	-1.234982	-0.002762	2.213457
B	-2.198002	1.505910	2.444871
B	-2.791924	0.142732	1.445285
B	-2.310095	-1.421093	2.204840
B	-3.931001	-0.809136	2.413993
B	-3.062967	-1.492860	3.788022
B	-2.382337	-0.161914	4.774600
B	-1.367387	-1.026632	3.634494
B	-1.288675	0.790562	3.775270
B	-2.941328	1.376001	4.022102
B	-3.859716	1.000259	2.572597

C	-3.905037	-0.025361	3.951235
H	-2.358964	-0.254041	5.954181
H	-3.455445	-2.472002	4.327634
H	-3.246223	2.276374	4.729472
H	-2.895380	0.245187	0.274418
H	-4.819181	1.621365	2.262790
H	-1.828261	2.501779	1.927813
H	-2.028400	-2.345863	1.525690
H	-4.934231	-1.294498	2.014890
H	-4.809711	-0.036660	4.541296
H	-0.322359	1.315110	4.205937
H	-0.444562	-1.687877	3.960507
H	1.343249	-0.006828	3.146273
H	3.405291	0.005018	1.856985
H	3.358020	0.019387	-0.656035
H	1.128929	0.005207	-1.808021
H	-0.941849	-0.007432	-0.533584

41

***p*-carborane X= S–[NMe$_4$]$^+$** scf done: -944.214636

C	0.383558	0.205873	0.355201
B	0.613620	0.024240	2.035193
B	2.314116	-0.418284	2.190916
B	1.061959	-1.675032	2.234380
B	1.014181	-2.452385	0.636348
C	2.513596	-1.896911	1.307659
S	3.715091	-3.121554	1.839249
B	-0.191745	-1.233608	1.071099
B	0.536551	-1.233097	-0.551700
B	2.240207	-1.674169	-0.391128
B	3.041919	-0.416760	0.572483
B	1.840312	0.803171	1.007782
B	1.791659	0.025708	-0.591415
C	6.875277	-3.085616	3.453371
N	6.439396	-1.785633	4.080801
C	7.504637	-1.280245	5.000646
C	6.184579	-0.783964	2.979555
C	5.158792	-2.012888	4.850449
H	1.949320	0.675015	-1.564832
H	2.033164	1.965401	1.096173
H	-0.141009	-1.416223	-1.500722
H	0.886480	-2.307209	3.213701
H	-1.350893	-1.417604	1.197515
H	0.804468	-3.607396	0.542803
H	2.974382	-0.200250	3.147097
H	-0.006890	0.672414	2.803078
H	-0.349708	0.928130	0.026857
H	2.854134	-2.305698	-1.173264
H	4.195057	-0.202314	0.431829
H	7.009904	-3.825557	4.241403
H	6.094759	-3.390948	2.754734

H	7.815019	-2.917522	2.928210
H	5.835121	0.144431	3.428426
H	7.118576	-0.619842	2.442571
H	5.421988	-1.215894	2.323301
H	4.846582	-1.063599	5.283796
H	4.410475	-2.386490	4.138446
H	5.357489	-2.741588	5.636197
H	7.173869	-0.341849	5.443660
H	7.678976	-2.017879	5.783368
H	8.419935	-1.118420	4.432245

41

***p*-carborane X= O–[NMe$_4$]$^+$** scf done: -621.244824

C	-0.002693	-0.003385	0.700425
B	0.218369	-0.509539	2.316009
B	1.968517	-0.639434	2.495481
B	0.991194	-2.089233	2.174667
B	1.140232	-2.481742	0.447251
C	2.512980	-1.840072	1.335062
B	2.209993	-1.274538	-0.299896
B	2.722018	-0.136250	0.965950
B	1.289330	0.698753	1.568065
B	1.438738	0.306033	-0.161025
B	0.460001	-1.145153	-0.481730
B	-0.294014	-1.649380	1.049108
H	1.481835	1.175263	-0.960138
O	3.543150	-2.592036	1.595141
H	1.233768	1.827655	1.912278
H	-0.144116	-1.235735	-1.493065
H	0.920649	-2.952559	2.979634
H	-1.396834	-2.073349	1.050275
H	1.170253	-3.608897	0.091360
H	2.555596	-0.528335	3.515706
H	-0.545620	-0.179835	3.154801
H	-0.857385	0.620630	0.484851
H	2.959435	-1.589880	-1.158318
H	3.815535	0.313681	0.958111
N	5.993403	-4.379290	2.215371
C	6.399482	-2.924202	2.214446
C	4.922540	-4.587028	3.260603
C	7.176673	-5.242385	2.514897
C	5.426757	-4.728575	0.859332
H	4.567056	-4.070470	0.696264
H	6.203272	-4.566530	0.112199
H	5.132263	-5.777879	0.871350
H	7.572634	-4.977858	3.494741
H	6.866636	-6.286783	2.511440
H	7.938277	-5.080646	1.752826
H	6.799762	-2.680439	3.198387
H	7.166536	-2.783794	1.453016
H	5.498307	-2.343413	1.991025

H 5.338842 -4.325740 4.233385
H 4.085913 -3.933508 2.991846
H 4.631514 -5.637228 3.247923

28
***p*-carborane X= CH= CH_2** scf done: -409.604096
C 0.046825 0.029435 0.008625
B -0.015911 -0.014470 1.714327
B 1.678554 -0.014697 2.204380
B 1.185106 -0.964457 0.800105
B 1.609430 -0.049357 -0.669846
B 0.669802 1.466584 -0.665251
B 2.363055 1.465430 -0.172072
C 2.328945 1.439145 1.549101
C 3.456403 2.135700 2.251613
B -0.334600 1.487706 0.807576
B 1.164166 2.418853 0.737763
B 0.739430 1.500835 2.208893
B 2.685769 -0.036373 0.730464
H -0.751482 -0.463702 -0.527493
H 3.246598 2.014681 -0.727271
H 2.106495 -0.452901 3.210462
H 3.776903 -0.479674 0.768853
H -0.897682 -0.562772 2.271960
H 0.539959 2.074658 3.218002
H 1.097794 -2.139064 0.757904
H 1.801769 -0.618613 -1.683714
H 0.242935 1.896705 -1.676078
H 1.252950 3.593077 0.780989
H -1.425417 1.932451 0.769952
C 3.608263 2.226750 3.568589
H 4.187174 2.589777 1.589513
H 4.457589 2.751880 3.989078
H 2.903960 1.789114 4.266340

26
***p*-carborane X= BH_2^-** scf done: -357.656734
C -0.002920 -0.001761 0.022226
B 0.006133 0.001715 1.722491
B 1.721428 0.024256 2.162504
B 0.795114 1.524424 2.164356
B 1.177055 2.400593 0.661092
B 2.318496 1.432022 -0.283501
C 2.402358 1.481631 1.475171
B 3.597488 2.218690 2.200258
H -0.821023 -0.506306 -0.471517
B 0.609376 1.415700 -0.705220
B -0.333517 1.486601 0.811988
B 1.537828 -0.087963 -0.707079

B	2.674199	-0.024055	0.658094
B	1.180552	-0.965450	0.808972
H	1.224314	3.581173	0.641527
H	3.750870	-0.510568	0.636459
H	3.160882	1.952959	-0.927426
H	1.089081	-2.145953	0.788324
H	2.155156	-0.416725	3.169125
H	1.687037	-0.700754	-1.709773
H	0.127233	1.825360	-1.706664
H	-1.429955	1.933655	0.793404
H	0.596289	2.107947	3.172245
H	-0.861807	-0.534919	2.324492
H	4.585880	1.592983	2.467487
H	3.481005	3.382111	2.469935

26

***p*-carborane X= $NH_2^{\cdot+}$** scf done: -387.556720

C	0.000195	0.000111	-0.014614
B	-0.027638	-0.017758	1.687757
B	1.675823	0.023826	2.150448
B	0.714310	1.516192	2.150407
C	2.292871	1.477249	1.450064
B	2.282501	1.470501	-0.291561
B	1.103902	2.412917	0.652381
B	-0.383449	1.469194	0.773314
B	0.581834	1.433811	-0.729638
B	1.546108	-0.062922	-0.729589
B	2.653429	0.007803	0.652436
B	1.179305	-0.956433	0.773403
N	3.333283	2.147586	2.170928
H	-0.800719	-0.515905	-0.524003
H	1.181267	3.590578	0.679379
H	3.757747	-0.408546	0.679535
H	3.147704	2.027893	-0.867955
H	1.116114	-2.132976	0.747724
H	2.142551	-0.376777	3.154638
H	1.727294	-0.657367	-1.731150
H	0.115464	1.844467	-1.731224
H	-1.480892	1.897988	0.747554
H	0.542459	2.106812	3.154572
H	-0.888464	-0.572345	2.271305
H	4.244424	1.751044	1.976495
H	3.349167	3.141051	1.975997

26

***p*-carborane X= $NH_2^{\cdot+}$** scf done: -387.224836

C	0.000000	0.000000	0.000000
B	0.000000	0.000000	1.709246
B	1.519578	0.000000	0.780709
B	1.298341	1.117197	2.128112

B	-0.455439	1.643396	2.140393
B	-0.705493	2.574933	0.580912
B	-1.240683	0.903662	0.752179
B	-0.476812	1.453422	-0.761458
B	0.819790	2.582063	-0.374130
C	0.825631	2.595999	1.377912
N	1.200870	3.771921	1.998715
B	1.237381	0.912275	-0.744291
B	2.090520	1.657280	0.602271
H	-0.292889	-0.920043	-0.487880
H	1.904873	1.174902	3.136473
H	-1.361877	3.552465	0.614763
H	-0.919552	2.060383	3.135332
H	-1.083705	1.451879	-1.767113
H	1.149699	3.563126	-0.931596
H	-2.362421	0.551008	0.758547
H	-0.312352	-0.942973	2.338217
H	2.214542	-0.947048	0.779483
H	3.186120	2.081786	0.636463
H	1.763022	0.576759	-1.741852
H	0.508889	4.478115	2.248336
H	2.140126	3.889841	2.377892

26

***p*-carborane X= BH_2 Y= S^-** scf done: -755.307152

C	0.000000	0.000000	0.000000
B	0.000000	0.000000	1.706803
B	1.521195	0.000000	0.772603
B	1.241056	0.908390	-0.738564
B	0.854452	2.578852	-0.315448
B	2.074933	1.670448	0.619501
B	1.307929	1.108320	2.131003
B	-0.386908	1.670539	2.131064
C	0.854818	2.579097	1.391835
B	-0.667194	2.579455	0.619011
B	-0.453382	1.470195	-0.739035
B	-1.220562	0.908583	0.772373
B	-0.408318	-1.233519	-0.665814
H	1.925386	1.103641	3.134750
H	-1.350075	3.539812	0.631039
H	-0.886015	2.034923	3.134495
S	1.263259	3.812601	2.057601
H	-1.070646	1.475641	-1.742903
H	1.174214	3.539769	-0.918083
H	-2.341646	0.545472	0.761916
H	-0.319436	-0.960603	2.310125
H	2.203892	-0.960536	0.760929
H	3.195546	2.033071	0.629750
H	1.739862	0.544201	-1.742259
H	0.415474	-1.684005	-1.089284
H	-0.832314	-1.871299	0.022927

27			
***p*-carborane X= BH$_2$ Y= SH** scf done: -755.834002			
C	0.000000	0.000000	0.000000
B	0.000000	0.000000	1.706803
B	1.521195	0.000000	0.772603
B	1.241056	0.908390	-0.738564
B	0.854452	2.578852	-0.315448
B	2.074933	1.670448	0.619501
B	1.307929	1.108320	2.131003
B	-0.386908	1.670539	2.131064
C	0.854818	2.579097	1.391835
B	-0.667194	2.579455	0.619011
B	-0.453382	1.470195	-0.739035
B	-1.220562	0.908583	0.772373
B	-0.408318	-1.233519	-0.665814
H	1.925386	1.103641	3.134750
H	-1.350075	3.539812	0.631039
H	-0.886015	2.034923	3.134495
S	1.263259	3.812601	2.057601
H	-1.070646	1.475641	-1.742903
H	1.174214	3.539769	-0.918083
H	-2.341646	0.545472	0.761916
H	-0.319436	-0.960603	2.310125
H	2.203892	-0.960536	0.760929
H	3.195546	2.033071	0.629750
H	1.739862	0.544201	-1.742259
H	2.192970	3.682402	2.481377
H	0.415474	-1.684005	-1.089284
H	-0.832314	-1.871299	0.022927
26			
***p*-carborane X= BH$_2$ Y= O$^-$** scf done: -432.327758			
C	0.000000	0.000000	0.000000
B	0.000000	0.000000	1.706803
B	1.521195	0.000000	0.772603
B	1.241056	0.908390	-0.738564
B	0.854452	2.578852	-0.315448
B	2.074933	1.670448	0.619501
B	1.307929	1.108320	2.131003
B	-0.386908	1.670539	2.131064
C	0.854818	2.579097	1.391835
B	-0.667194	2.579455	0.619011
B	-0.453382	1.470195	-0.739035
B	-1.220562	0.908583	0.772373
B	-0.408318	-1.233519	-0.665814
H	1.925386	1.103641	3.134750
H	-1.350075	3.539812	0.631039
H	-0.886015	2.034923	3.134495
O	1.263259	3.812601	2.057601
H	-1.070646	1.475641	-1.742903
H	1.174214	3.539769	-0.918083
H	-2.341646	0.545472	0.761916
H	-0.319436	-0.960603	2.310125

H	2.203892	-0.960536	0.760929
H	3.195546	2.033071	0.629750
H	1.739862	0.544201	-1.742259
H	0.415474	-1.684005	-1.089284
H	-0.832314	-1.871299	0.022927

27

***p*-carborane X= BH_2 Y= OH** scf done: -432.862519

C	0.000000	0.000000	0.000000
B	0.000000	0.000000	1.706803
B	1.521195	0.000000	0.772603
B	1.241056	0.908390	-0.738564
B	0.854452	2.578852	-0.315448
B	2.074933	1.670448	0.619501
B	1.307929	1.108320	2.131003
B	-0.386908	1.670539	2.131064
C	0.854818	2.579097	1.391835
B	-0.667194	2.579455	0.619011
B	-0.453382	1.470195	-0.739035
B	-1.220562	0.908583	0.772373
B	-0.408318	-1.233519	-0.665814
H	1.925386	1.103641	3.134750
H	-1.350075	3.539812	0.631039
H	-0.886015	2.034923	3.134495
O	1.263259	3.812601	2.057601
H	-1.070646	1.475641	-1.742903
H	1.174214	3.539769	-0.918083
H	-2.341646	0.545472	0.761916
H	-0.319436	-0.960603	2.310125
H	2.203892	-0.960536	0.760929
H	3.195546	2.033071	0.629750
H	1.739862	0.544201	-1.742259
H	2.192970	3.682402	2.481377
H	0.415474	-1.684005	-1.089284
H	-0.832314	-1.871299	0.022927

28

***p*-carborane X= BH_2 Y= NH_2** scf done: -412.993483

C	0.000000	0.000000	0.000000
B	0.000000	0.000000	1.706803
B	1.521195	0.000000	0.772603
B	1.241056	0.908390	-0.738564
B	0.854452	2.578852	-0.315448
B	2.074933	1.670448	0.619501
B	1.307929	1.108320	2.131003
B	-0.386908	1.670539	2.131064
C	0.854818	2.579097	1.391835
B	-0.667194	2.579455	0.619011
B	-0.453382	1.470195	-0.739035
B	-1.220562	0.908583	0.772373
B	-0.408318	-1.233519	-0.665814
H	1.925386	1.103641	3.134750
H	-1.350075	3.539812	0.631039

H	-0.886015	2.034923	3.134495
N	1.263259	3.812601	2.057601
H	-1.070646	1.475641	-1.742903
H	1.174214	3.539769	-0.918083
H	-2.341646	0.545472	0.761916
H	-0.319436	-0.960603	2.310125
H	2.203892	-0.960536	0.760929
H	3.195546	2.033071	0.629750
H	1.739862	0.544201	-1.742259
H	2.192970	3.682402	2.481377
H	1.303960	4.577289	1.368765
H	0.415474	-1.684005	-1.089284
H	-0.832314	-1.871299	0.022927

30

***p*-carborane X= BH_2 and Y= CH= CH_2** scf done: -435.040006

C	0.018879	0.030081	-0.066333
B	0.021796	-0.002343	1.647933
B	1.733118	-0.001817	2.103661
B	0.790270	1.517055	2.125839
C	2.371179	1.446859	1.429951
B	2.360238	1.470911	-0.298552
B	1.197004	2.426589	0.650945
B	-0.307538	1.512990	0.749203
B	0.655002	1.484725	-0.737284
B	1.583389	-0.048216	-0.763500
B	2.699555	-0.030183	0.611317
B	1.207575	-0.951778	0.722056
C	3.479281	2.170823	2.133705
C	4.480491	1.597075	2.793232
B	-1.156627	-0.681938	-0.813972
H	1.297676	3.600749	0.693771
H	3.789745	-0.476402	0.609154
H	3.230096	2.008889	-0.883974
H	1.116139	-2.126074	0.685138
H	2.169542	-0.428857	3.111036
H	1.763756	-0.616696	-1.781338
H	0.212217	1.919794	-1.739503
H	-1.395468	1.967037	0.743288
H	0.621532	2.085530	3.144247
H	-0.842800	-0.540073	2.243765
H	3.420169	3.253184	2.074574
H	5.240320	2.201863	3.273736
H	4.579840	0.521315	2.878318
H	-1.985202	-0.029949	-1.361025
H	-1.210247	-1.868455	-0.828011

28

***p*-carborane X= BH_2 Y= $BH_2^{\cdot-}$** scf done: -383.1143541

C	1.605587	0.000393	-0.000256
B	0.750214	1.492191	-0.207618

B	0.751862	0.264982	-1.487966
B	0.751137	-1.328863	-0.711148
B	-0.750223	-1.492114	0.207416
B	-0.751728	-0.659131	-1.359076
B	-0.751882	1.085885	-1.046959
B	-0.751061	1.329024	0.711293
B	0.751820	0.658930	1.358972
B	-0.751936	-0.264747	1.487902
C	-1.605575	-0.000402	0.000203
B	-3.166176	-0.000114	0.000122
B	0.751765	-1.086069	1.047074
B	3.166163	0.000064	-0.000013
H	-1.348562	-1.105909	-2.278108
H	1.350826	-2.228306	-1.191116
H	1.350702	-1.821458	1.754257
H	1.348447	1.105674	2.278166
H	1.350592	2.501327	-0.347644
H	1.347409	0.444685	-2.494849
H	-1.350534	-2.501331	0.347177
H	-1.347389	-0.444265	2.494878
H	-1.350950	2.228278	1.191336
H	-1.350554	1.821408	-1.754248
H	3.757410	-1.039327	0.000120
H	-3.758277	-1.038996	0.000617
H	-3.757515	1.039206	-0.000616
H	3.758549	1.038877	0.000347

28

***p*-carborane X= BH_2^- Y= BH_2^-** triplet state scf done: -383.015592

C	0.000000	0.000000	0.000000
B	0.000000	0.000000	1.599501
B	1.508390	0.000000	-0.882810
B	0.458877	-1.418875	-0.868045
B	-1.222264	-0.881942	-0.895309
B	-1.491155	0.011709	-2.400058
B	-1.207619	0.882955	-0.869891
B	-0.458993	1.443317	-2.373113
B	1.214076	0.881519	-2.396879
B	1.220394	-0.883353	-2.381470
B	-0.460796	-1.421148	-2.393785
C	0.006306	0.008445	-3.266793
B	0.009519	0.007461	-4.866278
B	0.470888	1.428067	-0.872582
H	-2.520872	0.023504	-2.987883
H	-2.045941	1.482905	-0.284005
H	0.799662	2.401999	-0.282057
H	2.535693	0.003913	-0.291779
H	0.771577	-2.403153	-0.284984
H	-2.058063	-1.482750	-0.308348
H	-0.775912	2.421875	-2.961358
H	2.049473	1.489905	-2.978695

H	2.047331	-1.494945	-2.970405
H	-0.770802	-2.401150	-2.984380
H	0.477945	-0.934108	2.188498
H	-0.458663	0.944621	2.187120
H	0.956046	0.462477	-5.453598
H	-0.925995	-0.467441	-5.455556

28

***p*-carborane X= BH_2^{2-} Y= BH_2^{2-}** scf done: -383.007780

C	-0.000000	0.000000	-0.000000
B	-0.000000	0.000000	1.748091
B	1.699325	-0.000000	2.264482
C	2.414566	1.483856	1.688628
B	3.551635	2.185793	2.483135
B	-1.137445	-0.701678	-0.794196
B	1.212891	-0.939832	0.839086
B	2.720109	-0.009085	0.816735
B	2.414572	1.483933	-0.059247
B	1.201443	2.423698	0.849509
B	0.763765	1.507291	2.282209
B	-0.305552	1.492587	0.871784
B	0.715487	1.483611	-0.576007
B	1.651145	-0.023442	-0.593623
H	1.251492	3.609116	0.876514
H	-1.388345	1.978159	0.850316
H	-0.871687	-0.548931	2.337382
H	1.163131	-2.125257	0.811824
H	1.903063	-0.571100	-1.616009
H	0.330950	1.967657	-1.589088
H	0.511470	2.054783	3.304590
H	2.083279	-0.483638	3.277979
H	3.803050	-0.494328	0.838312
H	3.286459	2.032480	-0.648594
H	-1.502824	-0.247944	-1.846139
H	-1.647494	-1.697716	-0.353752
H	3.917297	1.731784	3.534862
H	4.060718	3.182657	2.043437

28

***p*-carborane X= BH_2^{-} Y= BH_2^{-} rearranged** scf done: -383.033716

C	-0.000000	0.000000	0.000000
B	-0.000000	0.000000	1.496848
B	1.394941	-0.000000	-0.999932
B	0.995735	1.020503	-2.450048
B	0.019743	0.042246	-3.572880
B	-0.803386	1.101048	-2.439819
B	0.141037	1.327232	-0.947439
B	-1.342691	0.238784	-0.969850
B	-0.702566	-1.389714	-0.699466
B	0.669286	-1.864634	-1.548800
C	0.039312	-1.524149	-3.098476

B -0.010360 -2.707397 -4.013907
B 1.426506 -0.702746 -2.649556
B -1.318506 -0.639604 -2.533114
H 1.376042 -2.822679 -1.349218
H 2.457177 -0.987904 -3.185092
H -0.015188 0.279234 -4.744347
H -2.382701 -0.912878 -3.011117
H -1.478619 -2.082978 -0.087654
H 2.453517 0.139684 -0.456258
H 1.688465 1.884972 -2.907167
H -1.420662 2.035390 -2.866565
H -2.371932 0.558099 -0.451104
H 0.230726 2.395155 -0.417143
H 0.953622 -3.419430 -4.172369
H -1.023767 -3.029142 -4.593608
H -1.004056 -0.243166 2.124626
H 1.008028 0.194024 2.139342

28
***p*-carborane X= $NH_2^{\cdot -}$ Y= $NH_2^{\cdot +}$** scf done: -442.609775
C -0.000062 -0.000266 0.000073
B 0.000673 0.000113 1.713418
B 1.703330 0.000914 2.167235
B 1.205500 -0.986997 0.804184
B 1.561094 -0.074731 -0.713819
B 0.618841 1.452976 -0.720488
B 2.321541 1.453926 -0.266761
C 2.322288 1.454255 1.446627
N 3.363371 2.100850 2.097038
B -0.358946 1.507141 0.802656
B 1.116725 2.441021 0.642447
B 0.761226 1.528728 2.160490
N -1.041197 -0.646757 -0.650367
B 2.681156 -0.053051 0.644112
H 3.195061 1.993526 -0.843084
H 2.159852 -0.386903 3.179533
H 3.779266 -0.473786 0.655516
H -0.872811 -0.539506 2.289783
H 0.615202 2.112342 3.172233
H 1.111282 -2.158809 0.783636
H 1.707074 -0.658353 -1.725561
H 0.162406 1.840885 -1.732792
H 1.210959 3.612828 0.663064
H -1.457080 1.927800 0.791175
H 3.318179 2.289962 3.089889
H 4.181427 2.411158 1.588971
H -0.995819 -0.836214 -1.643143
H -1.858712 -0.958137 -0.142085

24
***m*-carborane X= O^-** scf done: -406.884617

C	0.004101	-0.003100	-0.032854
H	-0.016349	-0.000461	1.047937
B	1.531080	0.001929	-0.795839
B	1.261823	0.864671	-2.315340
B	0.474452	1.434973	-0.827118
B	-1.213953	0.869867	-0.850559
B	-1.194107	-0.903645	-0.825845
B	0.494723	-1.437678	-0.792777
C	-0.421608	-1.493588	-2.311337
O	-0.756517	-2.585347	-2.840271
B	1.279129	-0.909918	-2.283004
B	0.052178	-0.053375	-3.244302
B	-1.477815	-0.038685	-2.337575
B	-0.443273	1.403556	-2.349395
H	-2.038981	1.441916	-0.220382
H	2.511585	0.003203	-0.129778
H	0.044832	-0.150167	-4.424712
H	2.130878	1.486343	-2.834659
H	2.096020	-1.600859	-2.789140
H	-2.524467	-0.140383	-2.880798
H	-0.778027	2.405547	-2.892863
H	0.762586	2.386743	-0.183268
H	0.753495	-2.412956	-0.178263
H	-1.988117	-1.546414	-0.232454

26

***m*-carborane X= BH_2** scf done: -357.626228

C	0.042728	-0.007363	-0.028862
H	0.034049	-0.033591	1.051384
B	1.563868	0.040801	-0.810538
B	1.257181	0.953744	-2.311675
B	0.470247	1.453668	-0.800824
B	-1.211974	0.851670	-0.813175
B	-1.144393	-0.910290	-0.828956
B	0.558488	-1.407676	-0.827284
C	-0.342043	-1.331677	-2.275643
B	-0.733378	-2.673333	-2.979115
B	1.304750	-0.831531	-2.332555
B	0.061763	0.047628	-3.250816
B	-1.460512	-0.023660	-2.335151
B	-0.459460	1.455274	-2.313243
H	-2.049781	1.378703	-0.172724
H	2.552563	0.034304	-0.168430
H	0.028301	-0.070598	-4.423629
H	2.109653	1.591381	-2.823293
H	2.104168	-1.512550	-2.870974
H	-2.499899	-0.167567	-2.875260
H	-0.833717	2.451311	-2.825962
H	0.743582	2.391456	-0.139880
H	0.822665	-2.415217	-0.279816
H	-1.910302	-1.616931	-0.282427
H	-0.748684	-2.727454	-4.164431
H	-1.011008	-3.623431	-2.321589

24
***m*-carborane X= S$^-$** scf done: -729.865368

C	0.010437	0.016502	-0.026324
H	-0.012708	0.010177	1.054548
B	1.534190	0.018813	-0.785940
B	1.267067	0.885303	-2.305898
B	0.480823	1.454145	-0.819198
B	-1.206567	0.886275	-0.840297
B	-1.189251	-0.889072	-0.826335
B	0.498584	-1.423547	-0.793605
C	-0.399231	-1.417446	-2.263832
S	-0.874083	-2.964626	-2.998912
B	1.277288	-0.894808	-2.279614
B	0.057341	-0.036312	-3.236046
B	-1.467867	-0.026691	-2.333532
B	-0.435404	1.423846	-2.339701
H	-2.037356	1.451555	-0.214182
H	2.515776	0.010814	-0.123964
H	0.045946	-0.144396	-4.411406
H	2.138021	1.498956	-2.828179
H	2.085069	-1.588493	-2.787386
H	-2.508786	-0.134602	-2.877975
H	-0.775970	2.420511	-2.886103
H	0.769117	2.405349	-0.175656
H	0.747451	-2.400719	-0.185536
H	-1.976216	-1.539359	-0.239524

25
***m*-carborane X= SH** scf done: -730.392682

C	0.007589	0.024044	-0.015139
H	-0.010018	0.022648	1.065380
B	1.534814	0.011210	-0.784631
B	1.266829	0.888202	-2.309528
B	0.484201	1.452992	-0.819107
B	-1.212830	0.903979	-0.829995
B	-1.205494	-0.858909	-0.797961
B	0.489886	-1.411474	-0.771933
C	-0.398242	-1.337986	-2.226960
S	-0.965924	-2.884241	-2.996737
B	1.261863	-0.892564	-2.281690
B	0.051145	-0.004823	-3.240712
B	-1.479516	-0.005283	-2.326264
B	-0.435281	1.440155	-2.338323
H	-2.038216	1.472529	-0.209648
H	2.518230	-0.010755	-0.134959
H	0.019472	-0.159476	-4.407958
H	2.141100	1.484383	-2.833987
H	2.032355	-1.620161	-2.799433

H	-2.513230	-0.147503	-2.874360
H	-0.776001	2.431166	-2.882745
H	0.781639	2.398569	-0.180773
H	0.713011	-2.416921	-0.204724
H	-1.991207	-1.536931	-0.246740
H	0.247750	-3.468126	-3.044690

25

***m*-carborane X= OH** scf done: -407.421650

C	0.005756	0.014669	-0.008112
H	-0.031260	0.046405	1.071226
B	1.553064	0.038716	-0.744700
B	1.285765	0.847033	-2.304104
B	0.460798	1.439966	-0.846098
B	-1.219412	0.853815	-0.863690
B	-1.171187	-0.915548	-0.785441
B	0.550471	-1.421702	-0.711520
C	-0.320981	-1.412856	-2.174306
O	-0.665324	-2.657032	-2.675970
B	1.342938	-0.922506	-2.206794
B	0.102629	-0.123191	-3.209447
B	-1.447423	-0.102105	-2.326561
B	-0.435696	1.353197	-2.377937
H	-2.064781	1.419215	-0.267856
H	2.519765	0.071379	-0.071080
H	0.090488	-0.334235	-4.373338
H	2.143331	1.456770	-2.840334
H	2.123808	-1.655975	-2.698688
H	-2.459093	-0.308463	-2.895437
H	-0.782361	2.317031	-2.965804
H	0.720321	2.412285	-0.232217
H	0.783107	-2.407045	-0.114955
H	-1.945483	-1.604908	-0.232124
H	-0.615311	-2.630189	-3.638333

26

***m*-carborane X= NH_2** scf done: -387.552288

C	-0.020918	-0.022616	-0.092506
B	-0.140377	0.046687	1.594148
B	1.535341	0.057561	2.164506
B	2.323931	-1.434138	1.607125
B	2.338826	-1.444681	-0.161283
B	1.108270	-2.384390	0.725324
C	0.641980	-1.404918	2.056147
N	0.172358	-2.031266	3.257290
B	1.077006	0.992849	0.735908
B	1.569264	0.060366	-0.704776
B	0.646860	-1.468100	-0.714742
B	-0.404989	-1.466458	0.701958

B	2.605300	0.079664	0.736992
H	-0.812664	0.448625	-0.657028
H	0.233766	-1.919227	-1.722576
H	0.947199	2.163466	0.683958
H	3.068478	-1.992559	2.333687
H	3.647595	0.635011	0.763050
H	1.759880	0.494550	3.238491
H	1.051485	-3.557410	0.850203
H	3.190085	-1.982188	-0.779271
H	1.764080	0.625377	-1.721137
H	-1.058833	0.487884	2.180250
H	-1.482288	-1.933964	0.752451
H	0.358108	-3.026868	3.253916
H	0.595580	-1.620064	4.080419

34

***m*-carborane X= Ph**$^{\cdot -}$ scf done: -563.293581

C	0.079193	0.202966	0.239833
B	0.443379	0.108858	1.957728
B	2.227842	0.136951	2.038102
B	2.320191	-1.592796	1.467883
B	2.071170	-1.591524	-0.345562
B	0.860611	-2.381243	0.660311
C	0.673237	-1.587952	2.114978
C	0.316870	-2.293950	3.344539
B	1.508726	1.012062	0.761523
B	1.502626	0.060672	-0.732905
B	0.343035	-1.276393	-0.606773
B	-0.441121	-1.215559	0.967314
B	2.803086	-0.154650	0.444873
H	-0.707196	0.851368	-0.117270
H	-0.318139	-1.551847	-1.551332
H	1.488833	2.197422	0.693179
H	3.107809	-2.342034	1.945722
H	3.926634	0.124167	0.168820
H	2.808049	0.542932	2.989230
H	0.662302	-3.549633	0.649480
H	2.695406	-2.228159	-1.130866
H	1.564044	0.619270	-1.777299
H	-0.295778	0.705968	2.667557
H	-1.593557	-1.423827	1.132315
C	-0.723427	-1.838997	4.192014
C	-1.048334	-2.506578	5.365555
C	-0.349676	-3.654521	5.757697
C	0.686634	-4.115152	4.936874
C	1.016815	-3.452533	3.762266
H	-1.278478	-0.950960	3.915311
H	-1.856048	-2.126766	5.985081
H	-0.601654	-4.171250	6.677571
H	1.245552	-5.003080	5.218962
H	1.825990	-3.825851	3.147188

34			
***m*-carborane X= Ph** scf done: -563.290228			
C	0.042699	0.133479	0.105060
B	0.139753	0.099474	1.789226
B	1.851375	-0.122866	2.154029
B	2.349584	-1.668306	1.426593
B	2.170596	-1.554210	-0.335165
B	0.937813	-2.399352	0.625464
C	0.757692	-1.470877	2.079133
C	0.395750	-2.194947	3.359890
B	1.356674	0.951113	0.837964
B	1.552201	0.069775	-0.697583
B	0.443177	-1.324315	-0.685678
B	-0.429571	-1.310743	0.853178
B	2.735887	-0.141938	0.608631
H	-0.742969	0.738903	-0.323547
H	-0.141223	-1.645658	-1.657938
H	1.366408	2.129701	0.868205
H	3.113124	-2.354029	2.006114
H	3.841560	0.267560	0.536778
H	2.259865	0.213423	3.206951
H	0.743871	-3.560573	0.657690
H	2.874596	-2.145452	-1.076959
H	1.699025	0.679057	-1.696551
H	-0.646483	0.644597	2.470498
H	-1.550216	-1.635615	1.000034
C	-0.399079	-1.589626	4.339726
C	-0.719491	-2.265343	5.514989
C	-0.249806	-3.557639	5.734177
C	0.545343	-4.167527	4.766973
C	0.865578	-3.493103	3.591640
H	-0.771022	-0.584539	4.194263
H	-1.336507	-1.775622	6.259791
H	-0.498803	-4.082777	6.649156
H	0.920893	-5.172272	4.924014
H	1.486334	-3.982374	2.853155
28			
***m*-carborane X= CH= CH_2** scf done: -409.599323			
C	0.028108	-0.000856	-0.005981
B	-0.003046	0.060200	1.685044
B	1.691380	0.034798	2.174804
B	2.401426	-1.488128	1.579239
B	2.345225	-1.479395	-0.196515
B	1.134478	-2.397617	0.724360
C	0.751962	-1.414801	2.094145
C	0.357954	-2.128313	3.352977
B	1.191242	0.984627	0.771145

B	1.593568	0.050511	-0.693559
B	0.634736	-1.450588	-0.681129
B	-0.349177	-1.446791	0.786398
B	2.690339	0.029866	0.703220
H	-0.778945	0.494567	-0.526541
H	0.161558	-1.877681	-1.672945
H	1.084915	2.158112	0.729524
H	3.171944	-2.068687	2.257549
H	3.750019	0.551481	0.686274
H	1.988361	0.464673	3.230827
H	1.055723	-3.569329	0.835873
H	3.159702	-2.027778	-0.853133
H	1.756031	0.622417	-1.712001
H	-0.891811	0.520683	2.302230
H	-1.438323	-1.881744	0.876523
C	0.024166	-1.545913	4.499962
H	0.364233	-3.211039	3.277545
H	-0.245912	-2.144270	5.361878
H	0.012056	-0.469414	4.625039

41

***m*-carborane X= O–[NMe$_4$]$^+$** scf done: -621.241058

B	-0.490970	0.134575	-0.076070
B	-0.242186	0.062061	1.685575
B	1.073782	-0.384555	0.586600
B	1.402599	0.595350	2.030605
B	0.059475	1.713095	2.256371
C	-0.971188	1.418472	0.945704
B	-0.340877	1.833624	-0.587115
B	-0.001707	2.803687	0.857341
C	1.525208	2.276427	1.505717
O	2.305704	3.114456	2.125034
B	1.012484	0.715323	-0.824153
B	1.303612	2.367924	-0.242916
B	2.176233	1.004971	0.486453
C	4.773004	4.675271	1.628172
N	4.889061	4.528822	3.127282
C	4.949153	3.059300	3.471936
C	3.663649	5.131156	3.772636
C	6.119650	5.221882	3.617115
H	-0.896977	-0.658642	2.354877
H	1.452829	-1.503236	0.501030
H	-2.028761	1.568797	1.108954
H	1.878239	3.226055	-0.820087
H	-1.060684	2.277777	-1.412183
H	-0.418630	3.892403	1.034358
H	3.355181	0.943282	0.386845
H	1.348128	0.374523	-1.907496
H	-1.316049	-0.548860	-0.573020
H	-0.320011	2.133476	3.290673
H	2.043342	0.270854	2.970762

H	3.625800	6.188908	3.512606
H	2.794283	4.592865	3.385156
H	3.754883	5.015666	4.852555
H	5.826189	2.629089	2.989232
H	5.032020	2.964836	4.554525
H	4.023115	2.611916	3.096635
H	5.653283	4.224862	1.170376
H	3.855271	4.156202	1.332724
H	4.728680	5.738064	1.390812
H	6.992761	4.775876	3.142390
H	6.059946	6.279146	3.361023
H	6.188098	5.106583	4.698299

41

***m*-carborane X= S–[NMe$_4$]$^+$** scf done: -944.213228

B	0.009732	-0.115826	-0.101398
B	0.009500	-0.023995	1.676954
B	1.542429	-0.009120	0.787087
B	1.294812	1.083413	2.169363
B	-0.370564	1.647765	2.136744
C	-1.032245	0.946594	0.739424
B	-0.378456	1.503475	-0.733852
B	-0.609206	2.590046	0.649117
C	0.877685	2.640376	1.503113
S	1.290352	4.151997	2.383057
B	1.301260	0.938898	-0.710129
B	0.908385	2.612278	-0.242358
B	2.083839	1.681093	0.700218
C	4.714476	4.669921	1.661326
N	5.002077	4.391922	3.118667
C	4.517456	3.004287	3.467018
C	4.251404	5.387807	3.965078
C	6.470623	4.503219	3.379022
H	-0.433619	-0.903045	2.329007
H	2.305042	-0.912812	0.851543
H	-2.087772	0.716389	0.763691
H	1.223598	3.590429	-0.820289
H	-1.079467	1.630967	-1.675216
H	-1.403026	3.456473	0.682434
H	3.212647	2.033027	0.738337
H	1.892471	0.706948	-1.709089
H	-0.438257	-1.070229	-0.632946
H	-1.017401	1.941969	3.073427
H	1.865924	1.038748	3.202439
H	4.570336	6.391966	3.688264
H	3.187515	5.246027	3.770828
H	4.479833	5.194007	5.012616
H	5.027941	2.286666	2.827071
H	4.749512	2.814175	4.514741
H	3.437221	2.986018	3.291820
H	5.242318	3.931423	1.059789

H	3.629122	4.588124	1.520208
H	5.069949	5.673813	1.429413
H	6.995364	3.776130	2.760820
H	6.801915	5.510316	3.127857
H	6.662896	4.300605	4.432118

26

***m*-carborane X= $BH_2^{\cdot -}$** scf done: -357.656787

B	-0.054601	-0.129265	-0.075948
B	-0.039332	0.000168	1.696593
C	1.408103	0.121914	0.795375
B	2.052838	1.689904	0.664457
B	0.884429	2.578903	-0.291935
B	-0.424866	1.477996	-0.742118
B	1.242284	0.904197	-0.715459
B	-1.225402	0.913159	0.764520
B	-0.407898	1.667313	2.139823
C	0.904027	2.666015	1.480860
B	1.397951	3.965865	2.230556
B	-0.618126	2.596105	0.646202
B	1.260159	1.131081	2.156124
H	1.902735	0.543703	-1.630319
H	-1.025543	1.609444	-1.757470
H	2.109627	-0.697341	0.861006
H	-0.917794	2.000728	3.151861
H	-0.223305	-0.955817	2.371409
H	1.934270	0.973634	3.110979
H	-1.315822	3.550967	0.633388
H	-2.382779	0.651460	0.796761
H	-0.261294	-1.181446	-0.580170
H	3.216666	1.877948	0.697650
H	1.239385	3.522190	-0.908049
H	2.461263	3.936188	2.786954
H	0.702999	4.942723	2.223548

26

***m*-carborane X= $NH_2^{\cdot +}$** scf done: -387.219057

C	0.005225	0.013221	0.012226
B	0.015980	0.005388	1.691825
B	1.780148	-0.009354	2.144677
B	2.455634	-1.530523	1.445370
B	2.359085	-1.429667	-0.323654
B	1.186244	-2.441303	0.540723
C	0.825861	-1.486953	1.982838
N	0.507563	-2.171794	3.139095
B	1.184776	0.981564	0.804987
B	1.563792	0.115206	-0.719660
B	0.603252	-1.399346	-0.765309

B -0.344225 -1.469905 0.719040
B 2.715164 0.028098 0.637778
H -0.815838 0.529640 -0.466775
H 0.101056 -1.791537 -1.752751
H 1.063930 2.149779 0.846666
H 3.214946 -2.149276 2.102912
H 3.767433 0.556598 0.632207
H 2.073564 0.333231 3.230555
H 1.109548 -3.611319 0.628769
H 3.155821 -1.947321 -1.019258
H 1.687948 0.736993 -1.708782
H -0.843631 0.386485 2.396261
H -1.411520 -1.939754 0.862308
H 1.226639 -2.682163 3.649525
H -0.441380 -2.185646 3.508782

28
***m*-carborane X= $NH_2^{\cdot-}$ Y= $NH_2^{\cdot+}$** scf done: -442.598885
C -0.000387 0.008306 0.037641
B -0.013355 0.053853 1.759277
B 1.694233 -0.014990 2.228282
B 2.403231 -1.553969 1.606475
B 2.301024 -1.550123 -0.166121
B 1.074507 -2.438728 0.764376
C 0.737129 -1.449131 2.138420
N 0.388072 -2.076475 3.324868
N -1.005501 0.677281 -0.644362
B 1.205700 0.950308 0.836766
B 1.610743 0.012214 -0.650983
B 0.585977 -1.473396 -0.627123
B -0.400252 -1.459231 0.845232
B 2.691800 -0.021737 0.757044
H 0.084717 -1.869949 -1.616666
H 1.106128 2.123367 0.795165
H 3.155215 -2.154172 2.282079
H 3.753848 0.485270 0.735990
H 1.984303 0.388081 3.296508
H 0.962943 -3.605260 0.884662
H 3.089246 -2.114006 -0.834093
H 1.769282 0.584660 -1.665544
H -0.888299 0.519122 2.389179
H -1.497613 -1.863760 0.949896
H -1.541502 0.212182 -1.365521
H -1.169609 1.663565 -0.489847
H 0.480346 -1.597873 4.211459
H 0.109250 -3.048975 3.335003

30
***m*-carborane X= BH_2 and Y= CH= CH_2** scf done: -435.035938
C 0.046755 -0.043034 0.046117
B 0.040376 0.026051 1.750492

B	1.567725	0.032276	0.843939
B	1.298605	0.923879	-0.668147
B	0.874178	2.596542	-0.278848
B	-0.405846	1.431676	-0.713951
B	-0.663948	2.566912	0.620556
C	-0.332608	1.667374	2.056078
C	-1.046303	1.991389	3.334532
C	-2.313986	2.377129	3.436327
B	-1.173995	0.891024	0.790740
B	0.886350	2.747637	1.489571
B	1.313066	1.161812	2.182539
B	2.098941	1.727979	0.688936
B	-0.368885	-1.385256	-0.641993
H	2.255905	-0.925634	0.884470
H	-1.030267	1.407407	-1.715227
H	1.091826	3.728458	2.111312
H	1.142985	3.501212	-0.989220
H	-1.477005	3.419009	0.652646
H	1.795892	1.082676	3.255933
H	3.246099	2.008932	0.671368
H	1.804984	0.537258	-1.660272
H	-2.288026	0.526675	0.889938
H	-0.340452	-0.853097	2.433499
H	-0.436942	1.893311	4.227335
H	-2.746518	2.592168	4.406131
H	-2.958028	2.496730	2.572896
H	0.197829	-1.736573	-1.623627
H	-1.246311	-2.038195	-0.177763

28

***m*-carborane X= BH_2 and Y= NH_2** scf done: -412.988853

C	-0.049219	0.014204	0.054302
B	-0.022070	0.020705	1.776669
B	1.686927	-0.075984	2.215314
B	2.502360	1.353493	1.559976
B	1.318258	2.352550	0.716190
B	2.385899	1.276312	-0.224737
C	2.531470	-0.154115	0.709393
B	1.077905	-1.011301	0.846366
B	1.474768	-0.171224	-0.659789
B	0.671718	1.408567	-0.650906
B	-0.253585	1.536833	0.852019
B	0.860563	1.503588	2.221974
H	0.252760	1.832062	-1.669103
H	-0.909550	-0.476569	2.375339
H	3.512716	1.716680	2.052051
H	0.636814	2.068408	3.235130
H	2.136547	-0.663977	3.135243
H	3.296716	1.597105	-0.902962
N	3.736853	-0.917244	0.582604
H	1.418780	3.527484	0.647046

H	-1.319357	2.040799	0.832990
B	-1.284265	-0.649321	-0.640134
H	1.024156	-2.186089	0.805903
H	1.690333	-0.834442	-1.606322
H	-1.296722	-1.823827	-0.819264
H	-2.198429	0.027989	-0.977840
H	4.553231	-0.318919	0.548658
H	3.844973	-1.579910	1.340884

27

***m*-carborane X= BH₂ and Y= OH** scf done: -432.858192

C	-0.026860	0.007362	0.066752
B	0.026730	0.052884	1.787898
B	1.746071	-0.023991	2.181369
B	2.530393	1.416011	1.468113
B	1.286723	2.393253	0.658937
B	2.352350	1.353716	-0.310105
C	2.553654	-0.075286	0.649087
B	1.124710	-0.981107	0.840431
B	1.495387	-0.138458	-0.683449
B	0.626752	1.416487	-0.677838
B	-0.251311	1.539796	0.859600
B	0.913380	1.544996	2.192853
H	0.162888	1.812176	-1.688032
H	-0.835129	-0.455694	2.413035
H	3.551742	1.797191	1.927439
H	0.717364	2.122590	3.204159
H	2.245561	-0.587647	3.088245
H	3.243047	1.678906	-1.010496
O	3.718947	-0.813080	0.524704
H	1.354415	3.570098	0.586601
H	-1.328922	2.017189	0.861404
B	-1.254991	-0.684782	-0.614004
H	1.108684	-2.156017	0.806914
H	1.700580	-0.810944	-1.625499
H	-1.112616	-1.732037	-1.156466
H	-2.315691	-0.153972	-0.579840
H	4.436680	-0.339259	0.960337

27

***m*-carborane X= BH₂ and Y= SH** scf done: -755.829477

C	-0.062259	0.017578	0.065276
B	-0.006834	0.060834	1.785694
B	1.714294	-0.027658	2.185495
B	2.511648	1.404991	1.475590
B	1.277616	2.387740	0.659327
B	2.335916	1.334711	-0.306417
C	2.522061	-0.094543	0.651843
B	1.084923	-0.980372	0.843899
B	1.456476	-0.143094	-0.683536
B	0.606774	1.418879	-0.677287
B	-0.272034	1.552249	0.855988
B	0.889132	1.547089	2.190322

H	0.152476	1.818531	-1.690184
H	-0.871976	-0.441672	2.411380
H	3.522632	1.796323	1.942160
H	0.696040	2.124884	3.202113
H	2.191824	-0.592542	3.101585
H	3.219736	1.662775	-1.012700
S	4.041837	-1.080411	0.500287
H	1.360517	3.563390	0.582568
H	-1.345275	2.039909	0.856653
B	-1.297518	-0.668708	-0.608505
H	1.045369	-2.155476	0.812176
H	1.647606	-0.815929	-1.628683
H	-1.183369	-1.753931	-1.078064
H	-2.335789	-0.095310	-0.640678
H	4.868718	-0.109855	0.935845

26

***m*-carborane X= BH_2 and Y= O^-** scf done: -432.324904

C	-0.036062	0.004297	0.068206
B	0.057690	-0.005346	1.796483
B	1.772144	-0.053171	2.170532
B	2.521359	1.410302	1.493938
B	1.248634	2.378337	0.704911
B	2.341449	1.390879	-0.274696
C	2.686398	-0.129469	0.623124
B	1.140102	-0.990854	0.808664
B	1.490191	-0.103252	-0.694277
B	0.625939	1.435906	-0.644310
B	-0.289140	1.491541	0.887660
B	0.895426	1.482296	2.222320
H	0.132699	1.850029	-1.638494
H	-0.808591	-0.537504	2.404383
H	3.498121	1.861988	1.988088
H	0.675583	2.045319	3.244844
H	2.238739	-0.618417	3.099525
H	3.191069	1.797405	-0.991023
O	3.774674	-0.746861	0.511919
H	1.274786	3.565363	0.670675
H	-1.374143	1.968348	0.916543
B	-1.266089	-0.651811	-0.605569
H	1.081981	-2.169622	0.751801
H	1.648773	-0.732518	-1.681687
H	-1.948249	-1.419289	0.002069
H	-1.542126	-0.394413	-1.737439

26

***m*-carborane X= BH_2 and Y= S^-** scf done: -755.304394

C	-0.051991	0.013993	0.069409
B	0.039879	0.004470	1.793588
B	1.761328	-0.039153	2.169439
B	2.503293	1.421419	1.497459
B	1.229070	2.388589	0.707257
B	2.332197	1.394191	-0.264745

C	2.604520	-0.094333	0.628616
B	1.134331	-0.980161	0.816377
B	1.486968	-0.094514	-0.687690
B	0.611537	1.439783	-0.643681
B	-0.305864	1.501702	0.885824
B	0.874056	1.497269	2.220879
H	0.129546	1.850560	-1.643284
H	-0.819677	-0.532759	2.404170
H	3.482258	1.863153	1.987169
H	0.656831	2.054673	3.245445
H	2.231759	-0.602009	3.093155
H	3.185763	1.793046	-0.974467
S	4.137115	-0.967587	0.473943
H	1.261921	3.573839	0.665737
H	-1.391338	1.976566	0.910905
B	-1.281989	-0.646687	-0.608107
H	1.085034	-2.155082	0.754807
H	1.652463	-0.730177	-1.665039
H	-1.962127	-1.413893	0.000088
H	-1.555525	-0.387417	-1.739065

28

***m*-carborane X= BH_2 and Y= $BH_2^{\cdot-}$** scf done: -383.107658

C	-0.000015	-0.001681	-0.005129
B	-0.005587	-0.013005	1.713833
B	1.702426	-0.029131	2.214771
B	2.405013	-1.544483	1.621391
B	2.374268	-1.505238	-0.162602
B	1.172281	-2.443912	0.718843
C	0.752670	-1.489447	2.140018
B	0.304078	-2.151485	3.482158
B	-1.166731	0.770057	-0.702272
B	1.215598	0.935340	0.826626
B	1.609687	0.034066	-0.644898
B	0.687079	-1.482460	-0.665229
B	-0.315492	-1.483231	0.793782
B	2.706108	-0.006141	0.764964
H	0.236131	-1.909070	-1.672371
H	1.131306	2.115144	0.813379
H	3.195043	-2.132880	2.277409
H	3.773395	0.513133	0.756938
H	1.987554	0.404425	3.277700
H	1.108140	-3.623065	0.792056
H	3.212242	-2.025522	-0.823560
H	1.814264	0.610685	-1.657932
H	-0.879871	0.458157	2.346682
H	-1.399232	-1.943635	0.855548
H	-2.294741	0.407587	-0.542279
H	-0.921668	1.721159	-1.383444
H	1.094431	-2.283808	4.368813
H	-0.827202	-2.515130	3.612866

12			
**1,6-$C_2B_4H_6$** scf done: -179.284792			
B	0.063402	0.073450	-0.100676
B	-0.033398	-0.022773	1.607102
C	1.366279	0.283636	0.844281
B	0.750836	1.493187	1.736100
C	-0.553250	1.280876	0.791550
B	0.847124	1.589461	0.030687
H	1.274566	2.371669	-0.733946
H	1.083378	2.181674	2.627606
H	2.323502	-0.212907	0.870400
H	-1.509938	1.778376	0.764867
H	-0.460956	-0.807436	2.369495
H	-0.269609	-0.615836	-0.991208

16			
**1,6-$C_2B_4H_6$ X= CH= CH_2** scf done: -256.700886			
C	0.022988	-0.004192	0.008422
B	-0.007368	0.005167	1.628016
B	1.461782	0.029135	0.751768
C	0.874264	1.374362	1.462742
C	1.479279	2.289249	2.447912
B	-0.571199	1.334526	0.711169
B	0.892424	1.350425	-0.173845
H	-0.439706	-0.644699	2.505460
H	2.454090	-0.599566	0.777955
H	-0.401903	-0.684633	-0.711775
H	1.332598	2.005154	-1.043745
H	-1.562438	1.964544	0.686552
C	0.878837	3.366152	2.951650
H	2.488130	2.039183	2.765382
H	1.379840	3.999126	3.674436
H	-0.127071	3.649201	2.660269

22			
**1,6-$C_2B_4H_6$ X= Ph** scf done: -410.395280			
C	0.032586	0.400425	0.059740
C	-0.001333	0.000240	1.401117
C	1.190318	-0.400074	2.017809
C	2.388637	-0.400008	1.308438
C	2.413295	-0.000051	-0.026682
C	1.231590	0.400065	-0.648457
C	-1.275101	0.000347	2.154310
B	-2.775525	-0.522565	1.774625
B	-1.954163	-1.090888	3.161927
B	-1.665703	0.522648	3.652006
C	-3.149260	0.000048	3.262300
B	-2.485582	1.091070	2.263597
H	-2.739461	2.151404	1.826571
H	-1.123913	1.032171	4.561203
H	-4.077196	-0.000093	3.810978
H	-1.693293	-2.151135	3.595030
H	-3.310563	-1.031883	0.861322

H 1.175465 -0.712110 3.055994<br>H 3.303439 -0.712641 1.799283<br>H 3.346428 -0.000173 -0.578461<br>H 1.242354 0.712586 -1.686604<br>H -0.884219 0.712583 -0.427544
12<br>**1,6-$C_2B_4H_6$ X= S^-** scf done: -576.955744<br>B 0.026956 0.098035 -0.099790<br>B -0.049197 -0.007125 1.597915<br>C 1.385356 0.271754 0.825732<br>B 0.733953 1.496606 1.725778<br>C -0.591020 1.302131 0.800808<br>B 0.809448 1.602520 0.030755<br>H 1.210363 2.403384 -0.737697<br>H 1.058338 2.194536 2.620677<br>S 2.933703 -0.535467 0.844071<br>H -1.546655 1.801824 0.789602<br>H -0.493958 -0.788331 2.363396<br>H -0.338428 -0.579106 -0.994633
13<br>**1,6-$C_2B_4H_6$ X= OH** scf done: -254.520999<br>B 0.036893 0.067180 -0.118497<br>B 0.010643 -0.067127 1.595925<br>C 1.387006 0.241081 0.812094<br>B 0.775366 1.463653 1.735061<br>C -0.517825 1.255457 0.805753<br>B 0.886241 1.552129 0.024387<br>H 1.312942 2.337124 -0.736912<br>H 1.128572 2.138904 2.628397<br>O 2.615161 -0.356415 0.775696<br>H -1.467025 1.762871 0.802016<br>H -0.397353 -0.866301 2.355855<br>H -0.320730 -0.607538 -1.010436<br>H 2.612502 -1.140237 1.335005
13<br>**1,6-$C_2B_4H_6$ X= SH** scf done: -577.495181<br>B -0.019398 0.117716 -0.134529<br>B 0.005396 -0.049304 1.571118<br>C 1.356459 0.293961 0.758260<br>B 0.748381 1.489766 1.718957<br>C -0.570430 1.284230 0.822556<br>B 0.808771 1.614456 0.010736<br>H 1.205655 2.417371 -0.748089<br>H 1.120676 2.149893 2.615048<br>S 2.960072 -0.476224 0.656037<br>H -1.529155 1.776547 0.854682<br>H -0.368113 -0.867077 2.326590<br>H -0.383545 -0.544930 -1.032121

H	2.727626	-1.425625	1.585099

14
1,6-$C_2B_4H_6$ X= NH_2 scf done: -234.653032

B	-0.044755	0.048777	-0.102567
B	0.008649	-0.063346	1.607191
C	1.364508	0.186736	0.786067
B	0.790378	1.459278	1.694761
C	-0.523972	1.268945	0.807307
B	0.880162	1.489396	-0.016306
H	1.316023	2.240919	-0.808400
H	1.187447	2.143794	2.562418
N	2.604791	-0.476217	0.817654
H	-1.457238	1.806012	0.820066
H	-0.406005	-0.826660	2.399419
H	-0.451459	-0.630991	-0.970943
H	2.537021	-1.450058	1.081519
H	3.137616	-0.379608	-0.036361

12
1,6-$C_2B_4H_6$ X= O^- scf done: -253.968421

B	0.066993	0.074782	-0.101801
B	-0.019234	-0.027470	1.599453
C	1.468784	0.228913	0.833656
B	0.765120	1.481952	1.729697
C	-0.544739	1.278528	0.794838
B	0.851417	1.584403	0.029253
H	1.228037	2.400391	-0.742556
H	1.057064	2.198634	2.626650
O	2.584117	-0.352513	0.854777
H	-1.498035	1.775796	0.776954
H	-0.496814	-0.793178	2.367103
H	-0.323852	-0.589479	-1.001411

14
1,6-$C_2B_4H_6$ X= BH_2 scf done: -204.726055

B	-0.085257	0.045173	-0.094963
B	-0.098899	-0.005226	1.614814
C	1.299502	0.170015	0.732619
B	0.848228	1.418547	1.657584
C	-0.568698	1.313319	0.831776
B	0.755057	1.534196	-0.046481
H	1.166006	2.283311	-0.852456
H	1.308915	2.082681	2.509641
B	2.612430	-0.633456	0.663079
H	-1.488644	1.876289	0.880535
H	-0.516629	-0.750176	2.421173
H	-0.532923	-0.627031	-0.947660
H	2.759222	-1.599357	1.342859
H	3.484856	-0.291306	-0.070698

29
1,6-$C_2B_4H_6$ X= O–$[NMe_4]^+$ scf done: -468.334918

C	-0.321855	0.972676	0.626501
B	0.251281	0.185524	1.920378

C	1.363368	-0.455682	0.864810
O	2.348087	-1.290185	1.004511
H	-1.137577	1.663994	0.510977
B	1.244166	1.198763	0.970965
B	0.742815	0.372048	-0.435722
B	-0.250218	-0.641148	0.513494
C	3.379682	-3.963826	1.565146
N	4.692836	-3.241325	1.374225
C	4.723553	-2.646247	-0.014252
C	4.783771	-2.122375	2.385379
C	5.833042	-4.190295	1.552623
H	1.955015	2.114454	1.191755
H	-0.008261	0.110212	3.069063
H	-1.000435	-1.523495	0.287355
H	0.963602	0.480051	-1.589902
H	5.670337	-2.120946	-0.139623
H	4.648573	-3.459721	-0.735839
H	3.867810	-1.963493	-0.081082
H	6.770343	-3.653082	1.411691
H	5.795379	-4.609254	2.557648
H	5.751344	-4.989273	0.816431
H	5.729474	-1.601853	2.234627
H	3.924550	-1.465632	2.202478
H	4.752454	-2.560402	3.382970
H	3.363882	-4.382896	2.571219
H	2.588400	-3.217813	1.423216
H	3.318852	-4.762637	0.826071

29

1,6-$C_2B_4H_6$ X= S–$[NMe_4]^+$ scf done: -791.310386

C	-0.045036	0.099368	0.025042
B	0.000050	-0.091284	1.637652
C	1.629432	0.054608	1.465398
S	2.957362	0.046848	2.624712
H	-0.863842	0.124740	-0.675575
B	0.697896	1.268223	0.874465
B	1.559196	0.235165	-0.172405
B	0.863921	-1.116156	0.590490
C	3.484924	-3.414401	2.372615
N	4.771960	-3.107026	1.642535
C	4.464203	-2.274263	0.419778
C	5.668291	-2.306765	2.552803
C	5.448503	-4.378163	1.241187
H	0.604394	2.433403	1.002288
H	-0.771762	-0.250143	2.511236
H	0.934426	-2.285578	0.445737
H	2.315763	0.397811	-1.061381
H	5.398123	-2.087134	-0.109870
H	3.770419	-2.826636	-0.211457
H	4.016448	-1.339358	0.775055
H	6.375028	-4.140249	0.719520

H 5.664960 -4.964353 2.133766
H 4.786413 -4.938334 0.582174
H 6.598137 -2.095752 2.025455
H 5.139016 -1.382291 2.793408
H 5.866486 -2.892486 3.449783
H 3.725452 -4.018560 3.247367
H 3.039194 -2.452412 2.661961
H 2.829509 -3.963659 1.698391

14
1,6-$C_2B_4H_6$ X= $BH_2^{\cdot-}$ scf done: -204.742619
B -0.135338 0.083031 -0.089241
B -0.048068 -0.028151 1.602983
C 1.325408 0.153556 0.731173
B 0.793104 1.445978 1.657460
C -0.586866 1.326002 0.832822
B 0.800897 1.498898 -0.039829
H 1.194409 2.265992 -0.847426
H 1.227137 2.129508 2.517380
B 2.655429 -0.661896 0.660647
H -1.506976 1.890069 0.881659
H -0.490578 -0.765290 2.413167
H -0.614284 -0.573100 -0.946674
H 2.794678 -1.638884 1.350697
H 3.534213 -0.308733 -0.082994

22
1,6-$C_2B_4H_6$ X= $Ph^{\cdot-}$ scf done: -410.379523
C -0.034475 0.000574 -0.029933
B 0.057208 0.000142 1.673987
C 1.635106 0.000241 1.461527
B 0.779620 1.206498 0.698690
B 1.667675 0.000621 -0.130012
B 0.779632 -1.205688 0.698132
H -0.696932 -0.000063 2.581999
H 0.800147 2.386120 0.716661
C -1.105282 0.000782 -0.986805
H 2.439175 0.000078 2.179383
H 2.484464 0.000850 -0.982068
H 0.800293 -2.385318 0.715459
C -1.669576 1.223307 -1.490225
C -2.699238 1.214669 -2.410146
C -3.245460 0.001200 -2.898764
C -2.698999 -1.212483 -2.410943
C -1.669338 -1.221523 -1.491025
H -1.273752 2.170837 -1.136063
H -3.097117 2.163127 -2.765123
H -4.054177 0.001357 -3.621224
H -3.096692 -2.160786 -2.766542
H -1.273330 -2.169208 -1.137484

14

1,6-$C_2B_4H_6$ X= NH_2 $^{\cdot+}$ scf done: -234.326680

C	-0.114789	-0.006374	0.053471
B	0.031440	-0.092117	1.693414
B	1.418085	0.056554	0.657470
C	0.815700	1.326087	1.489926
N	1.337770	2.261102	2.322808
B	0.687106	1.348900	-0.201605
B	-0.762351	1.193629	0.881121
H	0.952698	2.134746	-1.026837
H	-1.728028	1.847547	0.975767
H	-0.562991	-0.696176	-0.645632
H	-0.265458	-0.775376	2.596508
H	2.452175	-0.484651	0.566209
H	0.848573	2.551168	3.166156
H	2.236294	2.697866	2.131543

18

1,6-$C_2B_4H_6$ X= BH_2 and Y= CH= CH_2 scf done: -282.143207

C	0.007584	0.014239	0.014246
B	0.007638	0.011557	1.629480
B	1.486103	0.050416	0.774046
C	0.925813	1.385283	1.480561
C	1.509725	2.319218	2.458775
C	2.731757	2.199855	2.977116
B	0.903479	1.344451	-0.177810
B	-0.519282	1.392368	0.769223
B	-0.628688	-0.943067	-1.007157
H	1.335098	1.991064	-1.058205
H	2.461520	-0.604998	0.774087
H	-0.431523	-0.636580	2.505010
H	-1.490247	2.054676	0.776474
H	0.878279	3.151171	2.758729
H	3.108792	2.919339	3.694476
H	3.391374	1.383535	2.702602
H	-1.730209	-0.742526	-1.410738
H	-0.023975	-1.889746	-1.400124

16

1,6-$C_2B_4H_6$ X= BH_2 and Y= NH_2 scf done: -260.099350

B	-0.060717	0.084810	-0.129300
B	0.028675	-0.098542	1.575302
C	1.376913	0.168828	0.779333
B	0.764653	1.446067	1.713186
C	-0.534768	1.283919	0.805495
B	0.928616	1.481120	0.004799
H	1.384215	2.246261	-0.763735
H	1.150065	2.110380	2.601764
N	2.597062	-0.499456	0.806219
B	-1.856537	2.053117	0.821936

H -0.397178 -0.880526 2.343955
H -0.470322 -0.565904 -1.018311
H -2.759266 1.675494 1.500241
H -1.987475 3.030653 0.154984
H 2.560407 -1.465896 1.095966
H 3.189195 -0.361851 0.000151

15
1,6-$C_2B_4H_6$ X= BH_2 and Y= OH scf done: -279.965881
B 0.029062 0.114504 -0.137432
B 0.033749 -0.090158 1.569501
C 1.401940 0.230537 0.813052
B 0.763037 1.454498 1.759602
C -0.516814 1.284529 0.810825
B 0.946785 1.554436 0.053927
H 1.396013 2.351291 -0.682241
H 1.107704 2.105198 2.674173
O 2.616146 -0.382007 0.776497
B -1.860533 2.024554 0.809833
H -0.388099 -0.903970 2.306316
H -0.333826 -0.527664 -1.050967
H -2.770860 1.610642 1.455343
H -1.995585 3.014682 0.163556
H 2.604047 -1.168797 1.332521

15
1,6-$C_2B_4H_6$ X= BH_2 and Y= SH scf done: -602.939537
B -0.004829 0.163592 -0.153995
B 0.035930 -0.060852 1.545554
C 1.386387 0.289613 0.771661
B 0.734435 1.493285 1.738417
C -0.558474 1.318858 0.808383
B 0.894341 1.613966 0.033337
H 1.322629 2.422839 -0.702356
H 1.089429 2.138091 2.652789
S 2.975087 -0.491941 0.700677
B -1.916109 2.036308 0.833425
H -0.360281 -0.887379 2.280880
H -0.364920 -0.476938 -1.069396
H -2.808304 1.599724 1.488617
H -2.076268 3.029102 0.197472
H 2.683715 -1.515991 1.529041

14
1,6-$C_2B_4H_6$ X= BH_2 and Y= O^- scf done: -279.425921
B 0.049341 0.054896 -0.065684
B -0.002176 -0.013449 1.617337
C 1.478716 0.213583 0.827883
B 0.819730 1.504080 1.704201
C -0.541935 1.308763 0.810862
B 0.871176 1.572777 0.021289
H 1.244345 2.352956 -0.786508
H 1.140891 2.215267 2.593887
O 2.577919 -0.382201 0.837229

B -1.867599 2.027510 0.799372
H -0.462693 -0.746762 2.423966
H -0.359332 -0.608750 -0.956219
H -2.907247 1.426568 0.743057
H -1.931906 3.227019 0.846106

14
1,6-$C_2B_4H_6$ X= BH_2 and Y= S^- scf done: -602.409100
B 0.022228 0.074926 -0.065815
B -0.024197 0.004563 1.615447
C 1.407709 0.252170 0.822842
B 0.792620 1.513641 1.701048
C -0.580671 1.329556 0.812623
B 0.839651 1.583619 0.019910
H 1.235349 2.349551 -0.785354
H 1.141278 2.208856 2.588070
S 2.926848 -0.571135 0.830937
B -1.913598 2.052465 0.805158
H -0.456003 -0.742091 2.420299
H -0.363144 -0.600911 -0.952648
H -2.947994 1.447287 0.752149
H -1.970846 3.249760 0.852112

16
1,6-$C_2B_4H_6$ X= BH_2 and Y= $BH_2^{\cdot -}$ scf done: -230.212765
B -0.018675 0.148415 -0.203863
B -0.110526 -0.108920 1.474432
C 1.344517 0.144078 0.731442
B 0.757714 1.315099 1.740303
C -0.605346 1.319448 0.804845
B 0.849837 1.572467 0.062069
H 1.327050 2.407032 -0.624144
H 1.145328 1.898730 2.691226
B 2.654127 -0.645488 0.682058
B -1.914899 2.109084 0.854551
H -0.587899 -0.943539 2.160487
H -0.406128 -0.435122 -1.154918
H -2.494845 2.267392 1.894811
H -2.382437 2.582420 -0.145889
H 3.121703 -1.118347 1.682724
H 3.234018 -0.804273 -0.358148

16
1,6-$C_2B_4H_6$ X= $NH_2^{\cdot -}$ and Y= $NH_2^{\cdot +}$ scf done: -289.726680
B 0.000000 0.000000 0.000000
B 0.000000 0.000000 1.658223
C 1.556875 0.000000 0.867692
B 0.580141 1.429808 1.660561
C -0.827834 1.355974 0.894380
B 0.835167 1.588085 0.011961
H 1.458228 2.651082 -0.007103
H 1.183817 2.499845 2.086167

N 2.835519 -0.660021 0.620624
N -2.073179 2.016489 0.514333
H -0.378635 -0.798164 2.167356
H -0.281470 -0.689619 -0.783943
H -2.826737 1.721172 1.151388
H -1.952661 3.037605 0.575139
H 2.923537 -1.479980 1.237730
H 2.880771 -0.963801 -0.362519

12
benzene scf done: -232.311238
C -0.000000 0.000000 0.029481
H -0.000000 0.000000 1.113269
C 1.207664 0.000000 -0.668500
C 1.207847 0.000000 -2.063086
C 0.000000 -0.000000 -2.760386
C -1.207847 -0.000000 -2.063086
C -1.207664 -0.000000 -0.668500
H 2.146892 0.000000 -0.126318
H 2.146939 0.000000 -2.605352
H 0.000000 -0.000000 -3.844853
H -2.146939 -0.000000 -2.605352
H -2.146892 -0.000000 -0.126318

14
benzene X= BH_2 scf done: -257.756626
C -0.065057 -0.051658 0.009288
C -0.028678 0.039705 1.401965
C 1.196488 0.119946 2.054924
C 2.413223 0.111068 1.338958
C 2.342123 0.017736 -0.067944
C 1.120656 -0.062773 -0.727700
H -0.953114 0.047917 1.968646
H 1.225263 0.190958 3.137223
B 3.764469 0.199357 2.064262
H 3.263881 0.009212 -0.640256
H 1.085689 -0.134014 -1.809127
H -1.019383 -0.114376 -0.502651
H 4.784128 0.190232 1.444126
H 3.806257 0.277683 3.254455

16
benzene X= CH= CH_2 scf done: -309.730769
C -0.033624 0.000000 0.025865
C 0.036330 0.000000 1.420705
C 1.281735 -0.000000 2.043725
C 2.444645 -0.000000 1.277114
C 2.392640 -0.000000 -0.125156
C 1.127819 0.000000 -0.736921
C 3.654400 -0.000000 -0.883223
H 1.349486 -0.000000 3.125901
H 3.411917 -0.000000 1.769281
H 1.049825 0.000000 -1.817834

H -0.999391 0.000000 -0.467399
H -0.871930 0.000000 2.012566
C 3.809362 -0.000000 -2.210906
H 4.550418 -0.000000 -0.266114
H 4.798165 -0.000000 -2.653265
H 2.972645 0.000000 -2.900524

22
benzene X= Ph scf done: -463.422730
C -0.009245 -0.219163 0.022155
C -0.003848 -0.000008 1.407778
C 1.230019 0.219149 2.038295
C 2.416514 0.219287 1.309441
C 2.395457 -0.000023 -0.067004
C 1.176848 -0.219319 -0.707351
C -1.269537 0.000009 2.185754
C -2.426779 0.610654 1.679872
C -3.613240 0.610934 2.408779
C -3.668855 0.000028 3.660515
C -2.526914 -0.610892 4.176160
C -1.340776 -0.610635 3.446726
H -2.389325 1.109972 0.718241
H -0.465877 -1.109969 3.847566
H -2.561281 -1.096929 5.144977
H -4.592431 0.000035 4.228199
H -4.493161 1.096974 2.001936
H 1.256754 0.414880 3.104330
H 3.357994 0.399875 1.816414
H 3.319028 -0.000028 -0.634696
H 1.149650 -0.399909 -1.776306
H -0.948331 -0.414878 -0.483087

14
benzene X= $\mathbf{NH_2}$ scf done: -287.687623
C -0.027928 -0.016354 0.023937
C 0.024146 0.037169 1.416527
C 1.245220 0.067416 2.082599
C 2.449272 0.045814 1.362815
C 2.393302 -0.009759 -0.037864
C 1.167076 -0.039618 -0.694518
H -0.894241 0.056153 1.993272
H 1.271224 0.103407 3.167296
N 3.678110 0.019197 2.029158
H 3.315895 -0.034100 -0.609485
H 1.147614 -0.080997 -1.778190
H -0.980763 -0.039105 -0.490997
H 4.472150 0.339411 1.494679
H 3.674870 0.392358 2.966942

13

benzene X= OH scf done: -307.558625

C	-0.008073	-0.000000	0.028851
C	0.026299	-0.000000	1.424415
C	1.238725	-0.000000	2.106083
C	2.434313	0.000000	1.385249
C	2.411416	0.000000	-0.010404
C	1.189507	0.000000	-0.682147
H	-0.899681	-0.000000	1.988643
H	1.278242	-0.000000	3.188627
O	3.601034	0.000000	2.103519
H	3.342869	0.000000	-0.569359
H	1.179968	0.000000	-1.766370
H	-0.955881	-0.000000	-0.495767
H	4.353377	0.000000	1.502669

13

benzene X= SH scf done: -630.525258

C	-0.056463	-0.000000	0.006867
C	-0.046225	0.000000	1.400894
C	1.157895	0.000000	2.099496
C	2.371987	0.000000	1.403854
C	2.363813	-0.000000	0.005346
C	1.153488	-0.000000	-0.684353
H	-0.979878	0.000000	1.952203
H	1.153906	0.000000	3.183814
S	3.879313	0.000000	2.364137
H	3.296389	-0.000000	-0.547621
H	1.160792	-0.000000	-1.768698
H	-0.995419	-0.000000	-0.533903
H	4.732519	-0.000000	1.321973

12

benzene X= O^- scf done: -306.993974

C	-0.035243	0.000000	0.017567
C	0.009779	0.000000	1.420446
C	1.212764	0.000000	2.111745
C	2.496410	-0.000000	1.443755
C	2.402563	-0.000000	0.000176
C	1.187636	-0.000000	-0.670627
H	-0.923795	0.000000	1.982960
H	1.224585	0.000000	3.199099
O	3.602116	-0.000000	2.067006
H	3.338474	-0.000000	-0.553511
H	1.185966	-0.000000	-1.760562
H	-0.981289	0.000000	-0.514721

12

benzene X= S^- scf done: -629.979070

C	-0.062634	0.000000	0.002245
C	-0.022910	0.000000	1.401102
C	1.188152	0.000000	2.085332
C	2.439010	-0.000000	1.411817
C	2.367304	-0.000000	-0.007226
C	1.154837	-0.000000	-0.688467

H	-0.952110	0.000000	1.967362
H	1.197566	0.000000	3.170218
S	3.961417	-0.000000	2.269658
H	3.300282	-0.000000	-0.560988
H	1.157027	-0.000000	-1.776613
H	-1.007975	0.000000	-0.531106

29

benzene X= O–[NMe$_4$]$^+$ scf done: -521.362570

C	0.132143	-0.282773	1.259063
C	1.115951	-0.636045	2.186876
C	2.428444	-0.792821	1.733375
C	2.753461	-0.603000	0.394507
C	1.772411	-0.242504	-0.571002
C	0.444980	-0.090270	-0.082065
O	2.072889	-0.062523	-1.830893
N	2.878670	0.311927	-4.768250
H	3.210788	-1.067715	2.435949
H	3.777907	-0.729122	0.055087
H	-0.330001	0.183105	-0.792665
H	-0.895911	-0.155646	1.587940
H	0.867124	-0.785041	3.231446
C	3.858646	1.023680	-3.864977
C	2.871426	-1.156385	-4.412451
C	3.268127	0.494651	-6.198695
H	4.850802	0.609284	-4.044534
H	3.845288	2.084554	-4.114930
H	3.520364	0.840611	-2.836597
H	3.871611	-1.554166	-4.584516
H	2.585018	-1.218024	-3.354964
H	2.151969	-1.660728	-5.057518
H	4.264655	0.082285	-6.353602
H	2.551077	-0.025043	-6.833425
H	3.267964	1.558084	-6.435849
C	1.497968	0.876360	-4.527099
H	1.274562	0.706595	-3.465070
H	1.515756	1.937924	-4.774126
H	0.797377	0.352747	-5.177776

29

benzene X= S–[NMe$_4$]$^+$ scf done: -844.336382

C	0.806413	1.227454	0.575377
C	2.034716	1.431080	1.207707
C	3.048769	0.490390	1.015753
C	2.845321	-0.620308	0.200144
C	1.617491	-0.838135	-0.465796
C	0.600422	0.117587	-0.240732
S	1.373554	-2.221070	-1.538319
N	2.619318	-0.168371	-4.447403
H	4.004941	0.616343	1.515361
H	3.635272	-1.354246	0.079999

H -0.367153 -0.038670 -0.706032
H -0.004168 1.934133 0.728021
H 2.191928 2.291897 1.847941
C 3.679735 -0.318327 -3.380998
C 3.122839 0.704015 -5.551429
C 1.378953 0.438041 -3.832670
H 3.880479 0.661960 -2.951954
H 3.280246 -1.004815 -2.625197
H 4.577490 -0.723316 -3.847912
H 3.365321 1.684818 -5.144081
H 4.014657 0.252535 -5.984945
H 2.348627 0.800252 -6.311988
H 1.644740 1.396976 -3.390920
H 0.638395 0.571564 -4.621172
H 1.023889 -0.262998 -3.068099
C 2.273858 -1.536101 -4.978114
H 3.171011 -1.978153 -5.410439
H 1.913720 -2.125502 -4.130603
H 1.500657 -1.429034 -5.738314

14
benzene X= $BH_2^{\cdot-}$ scf done: -257.774034
C -0.098886 -0.055105 -0.008260
C -0.030821 0.039020 1.396412
C 1.187633 0.119622 2.048952
C 2.448409 0.113641 1.358012
C 2.331706 0.016060 -0.071846
C 1.114295 -0.064569 -0.725946
H -0.951749 0.048645 1.977492
H 1.201797 0.191278 3.134234
B 3.785040 0.202893 2.074213
H 3.247956 0.005902 -0.657966
H 1.092716 -0.136600 -1.812312
H -1.054168 -0.118114 -0.519765
H 4.823639 0.195517 1.452269
H 3.838380 0.282801 3.280681

22
benzene X= $Ph^{\cdot-}$ scf done: -463.418278
C -0.062031 -0.000000 -0.036414
C -0.027879 -0.000000 1.371910
C 1.166620 -0.000000 2.069625
C 2.444969 -0.000000 1.411349
C 2.376177 0.000000 -0.024909
C 1.174854 -0.000000 -0.710760
H 1.121607 -0.000000 3.152333
C 3.691197 0.000000 2.130936
H 3.291481 0.000000 -0.605005
H 1.193090 0.000000 -1.798770
H -1.001179 -0.000000 -0.578792
H -0.961099 -0.000000 1.931553

C	4.969476	0.000000	1.472530
C	6.164029	0.000000	2.170128
C	6.198277	0.000000	3.578465
C	4.961473	0.000000	4.252932
C	3.760085	-0.000000	3.567178
H	5.014350	0.000000	0.389808
H	7.097218	0.000000	1.610418
H	7.137490	0.000000	4.120740
H	4.943311	-0.000000	5.340952
H	2.844845	-0.000000	4.147368

14

benzene X= $NH_2^{\cdot+}$ scf done: -287.410722

C	-0.002303	-0.002935	-0.030900
C	0.022046	-0.026638	1.381558
C	1.246973	-0.002979	2.085252
C	2.433828	0.043239	1.400979
C	2.418689	0.067125	-0.033304
C	1.170204	0.043282	-0.739479
N	3.567174	0.110152	-0.711315
H	1.244505	-0.021250	3.167949
H	3.383358	0.062181	1.923980
H	1.171016	0.062260	-1.823515
H	-0.951460	-0.021171	-0.551803
H	-0.910495	-0.063286	1.932086
H	3.585406	0.127294	-1.724185
H	4.462811	0.127267	-0.237950

16

benzene X= $NH_2^{\cdot-}$ Y= **$NH_2^{\cdot+}$** scf done: -342.821403

C	0.012483	0.046563	0.055237
C	-0.004292	0.035561	1.420149
C	1.215673	-0.000323	2.159902
C	2.453435	-0.024491	1.449832
C	2.470169	-0.013419	0.084920
C	1.250225	0.022434	-0.654842
N	1.199183	-0.011196	3.503325
N	1.266715	0.033301	-1.998221
H	3.384771	-0.051770	2.003742
H	3.414754	-0.031966	-0.446431
H	-0.918779	0.073885	-0.498770
H	-0.948948	0.054060	1.951401
H	0.412711	0.058331	-2.536828
H	2.133686	0.016785	-2.516039
H	0.332189	0.005835	4.021004
H	2.053208	-0.036949	4.041784

18

benzene X= BH_2 and Y= CH= CH_2 scf done: -335.177156

C -0.047145 -0.000000 -0.011829
C -0.014028 0.000000 1.372525
C 1.204490 0.000000 2.089987
C 2.392470 0.000000 1.330329
C 2.365453 0.000000 -0.057422
C 1.146615 -0.000000 -0.757493
C 1.175617 -0.000000 -2.227277
C 0.127793 -0.000000 -3.057960
H 3.347097 0.000000 1.845970
H 3.294858 -0.000000 -0.617871
H -1.003073 -0.000000 -0.522191
H -0.946985 0.000000 1.926567
B 1.233919 0.000000 3.624016
H 2.172742 -0.000000 -2.661343
H 0.272266 -0.000000 -4.131568
H -0.898882 -0.000000 -2.710009
H 2.276419 0.000000 4.206369
H 0.215246 0.000000 4.247159

16
benzene X= BH_2 and Y= NH_2 scf done: -313.138777
C -0.070764 -0.072577 0.013257
C -0.010886 -0.009095 1.419348
C 1.211818 0.080637 2.058628
C 2.435855 0.113899 1.349025
C 2.344118 0.050476 -0.061815
C 1.131663 -0.039755 -0.720218
H -0.932363 -0.028652 1.993068
H 1.236258 0.126590 3.142604
B 3.777235 0.212134 2.064012
H 3.258902 0.072721 -0.645250
H 1.095477 -0.083242 -1.804404
N -1.287166 -0.119945 -0.635786
H -1.298779 -0.413472 -1.599619
H -2.098309 -0.391910 -0.103474
H 4.795959 0.238668 1.439816
H 3.825536 0.263523 3.256979

15
benzene X= BH_2 and Y= OH scf done: -333.007598
C -0.080975 -0.019380 0.020393
C -0.038219 0.058319 1.416904
C 1.189933 0.122094 2.053094
C 2.410654 0.111599 1.337676
C 2.323185 0.031977 -0.069536
C 1.103230 -0.032855 -0.726232
H -0.969924 0.066943 1.969559
H 1.219627 0.182334 3.135870
B 3.760426 0.182829 2.050709
H 3.237792 0.021469 -0.652879
H 1.059551 -0.093794 -1.810045

O	-1.309297	-0.080726	-0.561512
H	-1.218777	-0.131908	-1.519491
H	4.776576	0.172203	1.423159
H	3.812820	0.249887	3.241835
15			
**benzene X= BH_2 and Y= SH** scf done: -655.972856			
C	-0.072330	-0.015222	0.080936
C	0.002434	0.058600	1.479922
C	1.240262	0.120503	2.100782
C	2.447057	0.112373	1.366821
C	2.336355	0.037451	-0.038595
C	1.106862	-0.025678	-0.677553
H	-0.906912	0.067225	2.070611
H	1.284176	0.177176	3.183365
B	3.810565	0.181414	2.061045
H	3.241265	0.028926	-0.637285
H	1.059570	-0.082849	-1.759293
S	-1.688164	-0.093110	-0.653229
H	-1.280023	-0.142803	-1.936429
H	4.815862	0.173536	1.417295
H	3.879623	0.243450	3.251111
14			
**benzene X= BH_2 and Y= O^-** scf done: -332.468667			
C	-0.141279	-0.054059	-0.021272
C	-0.025081	0.041257	1.425933
C	1.189720	0.118854	2.055316
C	2.441043	0.113395	1.354849
C	2.329633	0.019283	-0.071821
C	1.129830	-0.059818	-0.729159
H	-0.954608	0.048184	1.989335
H	1.220357	0.188537	3.141663
B	3.757339	0.199311	2.055862
H	3.252409	0.011407	-0.650040
H	1.083026	-0.130260	-1.812835
O	-1.248062	-0.125938	-0.611028
H	4.795446	0.193056	1.441856
H	3.823329	0.277781	3.257512
14			
**benzene X= BH_2 and Y= S^-** scf done: -655.446023			
C	-0.096484	-0.047213	0.004190
C	0.005001	0.044178	1.428062
C	1.221643	0.119934	2.067025
C	2.465217	0.113234	1.366555
C	2.355628	0.021804	-0.053569
C	1.145713	-0.054566	-0.705077
H	-0.918629	0.052153	1.997257
H	1.244985	0.187937	3.152580
B	3.789913	0.195979	2.071462
H	3.272524	0.012692	-0.639091
H	1.104992	-0.123170	-1.787096
S	-1.613729	-0.142695	-0.802830

H 4.823944 0.188491 1.455727
H 3.852386 0.272236 3.270973

16
benzene X= BH_2 and Y= $BH_2^{\cdot -}$ scf done: -283.253443
C -0.072584 -0.121809 0.013233
C 0.040643 -0.054644 1.442073
C 1.250232 0.059849 2.086134
C 2.499329 0.121737 1.382685
C 2.386116 0.054577 -0.046212
C 1.176559 -0.060014 -0.690256
H -0.875338 -0.097069 2.027869
H 1.275216 0.106422 3.172957
B 3.826580 0.247521 2.089277
H 3.302140 0.097087 -0.631929
H 1.151486 -0.106728 -1.777068
B -1.399738 -0.247417 -0.693568
H -1.460034 -0.301429 -1.896812
H -2.432268 -0.296757 -0.072509
H 4.858934 0.297126 1.467844
H 3.887280 0.301546 3.292452

18
cyclohexane scf done: -235.944826
C 0.025947 -0.218241 0.071678
C 0.026615 -0.087729 1.602287
H 0.644548 0.580923 -0.357765
C -0.677829 1.198592 2.059473
H -0.488805 -0.954240 2.037467
H 1.052777 -0.113365 1.983839
C -2.099012 1.298492 1.484961
H -0.707557 1.245673 3.153176
H -0.093379 2.066617 1.726574
C -2.099783 1.167863 -0.045658
H -2.717635 0.499302 1.914521
H -2.562679 2.244371 1.784369
C -1.395246 -0.118393 -0.502801
H -3.126012 1.193356 -0.427089
H -1.584572 2.034413 -0.481000
H -1.979642 -0.986423 -0.169777
H -1.365612 -0.165534 -1.596492
H 0.489684 -1.164050 -0.227762

19
cyclohexane X= SH scf done: -634.160101
C -0.026189 0.132391 0.036470
C 0.020361 0.220147 1.567960
C 1.460017 0.110026 2.088353
C 2.155474 -1.160707 1.573673
C 2.107325 -1.235118 0.041413
C 0.667712 -1.140715 -0.476951
H -0.433527 1.156608 1.907957
S 2.952133 -2.802149 -0.480638
H -0.581519 -0.592326 1.994919

H 2.030680 0.990662 1.766722
H 1.471888 0.116053 3.182862
H 3.193496 -1.191301 1.917798
H 1.655564 -2.044619 1.989167
H 2.696860 -0.408781 -0.367546
H 0.659776 -1.158953 -1.571537
H 0.114462 -2.026265 -0.143295
H 0.466196 1.014558 -0.392789
H -1.062274 0.153759 -0.316197
H 2.797703 -2.629321 -1.809819

18
cyclohexane X= S^- scf done: -633.590553
C -0.038195 0.136081 0.038686
C 0.023635 0.234117 1.570134
C 1.468906 0.110262 2.074860
C 2.148442 -1.166449 1.543062
C 2.109311 -1.275913 0.007212
C 0.653322 -1.140944 -0.476695
H -0.427351 1.174346 1.915894
S 2.922364 -2.809394 -0.613997
H -0.575606 -0.579047 2.003399
H 2.038152 0.990529 1.742392
H 1.487413 0.126666 3.173449
H 3.189506 -1.215066 1.876350
H 1.650983 -2.053066 1.958347
H 2.665393 -0.413812 -0.393563
H 0.638048 -1.171636 -1.570349
H 0.100042 -2.026496 -0.136541
H 0.456890 1.017556 -0.394161
H -1.083111 0.170838 -0.299625

19
cyclohexane X= OH scf done: -311.188742
C -0.015625 0.117728 0.016600
C 0.046481 0.196511 1.548786
C 1.490708 0.077595 2.056830
C 2.176477 -1.189296 1.523555
C 2.116893 -1.257209 -0.000003
C 0.674061 -1.152841 -0.505338
H -0.401800 1.132824 1.896935
O 2.734520 -2.489040 -0.393008
H -0.554954 -0.616145 1.976152
H 2.058981 0.961629 1.739327
H 1.509938 0.076152 3.150997
H 3.220927 -1.234244 1.845312
H 1.681067 -2.082306 1.923365
H 2.696754 -0.415369 -0.411375
H 0.662187 -1.168265 -1.602349
H 0.128497 -2.042230 -0.167302
H 0.475347 1.001402 -0.411396
H -1.054718 0.145525 -0.325875

H 2.700396 -2.558472 -1.352690

18
cyclohexane X= O^- scf done: -310.579526
C -0.021271 0.116335 0.022218
C 0.040466 0.219605 1.554283
C 1.484522 0.091538 2.064113
C 2.153576 -1.193117 1.546461
C 2.142681 -1.342334 -0.014915
C 0.656159 -1.168364 -0.484273
H -0.406696 1.164380 1.895531
O 2.714375 -2.467949 -0.450213
H -0.564315 -0.588558 1.990538
H 2.057917 0.963304 1.716496
H 1.498032 0.133320 3.163591
H 3.197298 -1.251876 1.877272
H 1.642768 -2.077186 1.956039
H 2.657775 -0.393662 -0.383151
H 0.648087 -1.209681 -1.579935
H 0.103663 -2.051815 -0.131395
H 0.490216 0.989214 -0.409111
H -1.067111 0.175420 -0.314695

20
cyclohexane X= NH_2 scf done: -291.313390
C -0.009854 0.107309 0.015139
C 0.046107 0.190119 1.547370
C 1.489408 0.086487 2.060945
C 2.185330 -1.174475 1.528831
C 2.141495 -1.246959 -0.002884
C 0.694837 -1.153939 -0.504675
H -0.413349 1.121949 1.893473
N 2.740896 -2.510415 -0.455097
H -0.547632 -0.628613 1.974125
H 2.050420 0.975286 1.743741
H 1.503825 0.086568 3.155541
H 3.226906 -1.205016 1.870960
H 1.696191 -2.071603 1.927176
H 2.686911 -0.369057 -0.393777
H 0.681853 -1.170318 -1.601233
H 0.158968 -2.050252 -0.169643
H 0.470309 0.997148 -0.412500
H -1.049210 0.121934 -0.328137
H 2.741321 -2.568208 -1.469142
H 3.707494 -2.581466 -0.151359

20
cyclohexane X= BH_2 scf done: -261.376381
C -0.039159 0.126893 0.036035
C 0.017305 0.201595 1.568270
C 1.460662 0.106925 2.082386
C 2.168880 -1.151430 1.559584

C 2.109605 -1.266116 0.020435
C 0.660865 -1.131398 -0.497844
H -0.449905 1.127543 1.919729
B 2.976124 -2.382321 -0.625528
H -0.571014 -0.623521 1.991397
H 2.018427 0.994508 1.754519
H 1.473950 0.118884 3.177588
H 3.210334 -1.169077 1.895812
H 1.689773 -2.037425 1.997541
H 2.678272 -0.393978 -0.387873
H 0.653646 -1.135131 -1.592340
H 0.090772 -2.016170 -0.183988
H 0.449761 1.015351 -0.385879
H -1.079348 0.152957 -0.306179
H 2.734271 -2.782918 -1.725794
H 3.949005 -2.798690 -0.069016

28
cyclohexane X= Ph scf done: -467.050863
C -0.046377 -0.331694 0.223037
C 0.111078 0.193343 1.656555
H 0.346714 -1.350842 0.146131
C -0.499421 1.593259 1.808129
H -0.388996 -0.495509 2.350164
H 1.168303 0.207706 1.941284
C -1.964976 1.625732 1.351471
H -0.425424 1.929750 2.847549
H 0.081288 2.307145 1.209202
C -2.127831 1.109215 -0.095605
H -2.568221 1.005684 2.026257
H -2.364791 2.642209 1.424720
C -1.511573 -0.300819 -0.234880
C -3.567628 1.165038 -0.574930
H -1.548071 1.779618 -0.744082
H -2.093752 -1.010337 0.366248
H -1.593480 -0.635319 -1.274035
H 0.557109 0.285282 -0.455727
C -3.924782 1.973728 -1.659459
C -5.242237 2.037023 -2.112910
C -6.233748 1.287044 -1.485194
C -5.894858 0.475687 -0.402680
C -4.577316 0.416724 0.045536
H -3.159723 2.562586 -2.156162
H -5.491917 2.672205 -2.955904
H -7.259197 1.333114 -1.834260
H -6.658733 -0.112861 0.093970
H -4.335581 -0.220907 0.889241

28
cyclohexane X= Ph$^{\cdot-}$ scf done: -467.017101
C 0.092782 -0.120232 -0.038337
C 0.019069 -0.078089 1.495220
C 1.403046 0.057057 2.138806

C	2.173182	1.249305	1.561994
C	2.262005	1.178492	0.032470
C	0.869031	1.089726	-0.616423
C	0.870670	1.102217	-2.135424
H	1.307082	0.160780	3.229497
H	1.977399	-0.863308	1.968059
H	1.672202	2.180705	1.854437
H	3.183443	1.296200	1.992268
H	2.801956	2.055740	-0.347657
H	2.858662	0.302595	-0.249893
H	0.315961	1.989940	-0.298672
H	-0.921780	-0.147283	-0.454897
H	0.588372	-1.043504	-0.364688
H	-0.604145	0.772604	1.799299
H	-0.490506	-0.976483	1.867241
C	-0.344859	1.316907	-2.816253
C	-0.403475	1.331388	-4.204655
C	0.752971	1.138996	-4.963846
C	1.975095	0.994143	-4.305093
C	2.032329	0.980800	-2.910515
H	-1.257261	1.474071	-2.244841
H	-1.359962	1.489938	-4.698004
H	0.704828	1.127740	-6.049596
H	2.893188	0.882868	-4.876153
H	2.999809	0.867419	-2.432740

20

cyclohexane X= $NH_2^{\cdot+}$ scf done: -291.010058

C	0.000000	-0.000000	-0.000000
C	0.000000	-0.000000	1.541126
C	1.418508	0.000000	2.112456
C	2.239711	-1.201202	1.625448
C	2.249248	-1.256450	0.097474
C	0.859745	-1.332125	-0.517818
N	-1.266531	-0.090897	-0.568722
H	3.262601	-1.130768	2.000468
H	1.819089	-2.129663	2.027347
H	2.803131	-0.410552	-0.321375
H	2.760235	-2.169037	-0.245049
H	0.870165	-1.289938	-1.606673
H	0.302092	-2.210600	-0.187287
H	0.533000	0.848720	-0.435376
H	-0.541900	0.894301	1.864277
H	-0.559162	-0.871289	1.902583
H	1.922541	0.935123	1.844104
H	1.351340	-0.011715	3.203809
H	-1.992539	-0.662107	-0.143794
H	-1.477569	0.307375	-1.479151

22

cyclohexane X= $NH_2^{\cdot -}$ Y= $NH_2^{\cdot +}$scf done: -346.386692

C	-0.043929	-0.064902	0.038260
C	-0.021219	-0.076562	1.587131
C	1.440054	-0.004542	2.096408
C	2.253143	-1.202347	1.544973
C	2.224530	-1.205235	0.003472
C	0.774276	-1.246904	-0.518702
N	3.603581	-1.153037	2.029580
N	-1.396702	-0.110891	-0.440449
H	1.813621	-2.130948	1.920280
H	2.725983	-0.302423	-0.364612
H	2.784899	-2.060885	-0.381827
H	0.770121	-1.225950	-1.611479
H	0.297088	-2.186082	-0.214960
H	0.387027	0.876357	-0.314774
H	-0.592568	0.763003	1.993882
H	-0.499637	-0.994982	1.943508
H	1.909401	0.927557	1.764330
H	1.446679	0.003319	3.190315
H	3.917380	-1.741609	2.788921
H	4.224107	-0.401929	1.755197
H	-1.869535	0.716500	-0.777102
H	-1.974116	-0.926711	-0.280650

35

cyclohexane X= O–$[NMe_4]^+$ scf done: -524.948605

C	0.091924	-0.193976	-0.679933
C	-0.210157	0.395047	0.706815
C	1.079792	0.669518	1.494025
C	2.045686	1.552603	0.690151
C	2.331816	0.955396	-0.696479
C	1.052465	0.679242	-1.529864
O	1.328272	0.124215	-2.748132
H	0.847365	1.136362	2.458960
H	1.572002	-0.286453	1.719711
H	1.599642	2.549844	0.571388
H	2.978204	1.697905	1.249894
H	2.985175	1.618120	-1.276925
H	2.866024	0.000330	-0.591137
H	0.540550	1.675499	-1.624332
H	-0.834174	-0.341730	-1.248711
H	0.557505	-1.184257	-0.574083
H	-0.761562	1.338224	0.588830
H	-0.865375	-0.274375	1.278286
N	1.612491	-0.475977	-5.961787
C	0.971773	0.780813	-5.418966
C	2.790624	-0.102252	-6.806311
C	0.611834	-1.220069	-6.789979
C	2.076598	-1.364002	-4.825296
H	2.529793	-2.253550	-5.265203
H	2.752822	-0.795212	-4.190128
H	1.227714	-1.577797	-4.178819

H	0.654824	1.386001	-6.270468
H	0.156683	0.495203	-4.758050
H	1.687020	1.280386	-4.769661
H	2.459505	0.535609	-7.625495
H	3.503472	0.434005	-6.183079
H	3.251017	-1.007966	-7.199971
H	0.277133	-0.584062	-7.609149
H	1.074325	-2.124715	-7.183636
H	-0.232079	-1.482477	-6.155081
35			
**cyclohexane X= S–[NMe$_4$]$^+$** scf done: -847.954867			
C	0.251708	-0.279093	-0.306461
C	0.595626	0.231225	1.103936
C	1.993614	0.863596	1.152527
C	2.142914	1.962201	0.090758
C	1.790412	1.442806	-1.314576
C	0.386445	0.819813	-1.372568
S	-0.061495	0.203303	-3.055819
H	2.197363	1.267442	2.150979
H	2.746545	0.084355	0.971159
H	1.478048	2.798975	0.343721
H	3.163290	2.364134	0.100625
H	1.860006	2.252157	-2.048292
H	2.521383	0.678863	-1.613937
H	-0.338013	1.608602	-1.134190
H	-0.763500	-0.684517	-0.331718
H	0.922516	-1.105826	-0.574737
H	-0.147469	0.979949	1.410041
H	0.525126	-0.588267	1.828858
N	1.493575	-0.645654	-6.246473
C	0.477408	0.462833	-6.396252
C	2.604404	-0.165928	-5.343983
C	2.038469	-1.017351	-7.587564
C	0.836638	-1.848143	-5.608977
H	1.576511	-2.646166	-5.547545
H	0.498345	-1.553680	-4.605751
H	0.001041	-2.155756	-6.237075
H	0.961892	1.302050	-6.895253
H	-0.349474	0.090871	-7.000610
H	0.141680	0.750285	-5.390481
H	3.068528	0.706617	-5.803455
H	2.137303	0.086319	-4.380359
H	3.331780	-0.970463	-5.236547
H	2.512930	-0.144444	-8.034541
H	2.771373	-1.814254	-7.466717
H	1.220329	-1.359487	-8.221063
20			
**cyclohexane X= $BH_2^{\cdot-}$** scf done: -261.369466			
C	-0.016766	-0.004812	0.016086
C	-0.012419	-0.053745	1.551796
C	1.417533	-0.004984	2.111869

C	2.302721	-1.100556	1.492059
C	2.328304	-1.068439	-0.050207
C	0.881380	-1.100423	-0.584738
H	-0.617665	0.764449	1.966084
B	3.303583	-2.164049	-0.717749
H	-0.487500	-0.989998	1.876849
H	1.852599	0.981520	1.893751
H	1.392912	-0.098833	3.206909
H	3.326237	-1.019769	1.876500
H	1.936665	-2.087337	1.812240
H	2.729083	-0.071244	-0.324483
H	0.893582	-1.019578	-1.677999
H	0.450321	-2.087186	-0.359501
H	0.344173	0.981690	-0.310388
H	-1.046432	-0.098527	-0.357444
H	3.150433	-2.466772	-1.877818
H	4.328499	-2.468493	-0.154102

24

cyclohexane X= BH_2 and Y= C= CH_2 scf done: -338.790536

C	-0.014194	0.058000	0.023163
C	0.060983	-0.130434	1.552593
C	1.503755	-0.138167	2.076160
C	2.382571	-1.184460	1.356700
C	2.325895	-0.963730	-0.170592
C	0.887270	-0.960167	-0.705546
C	3.798763	-1.147994	1.861542
B	-1.426926	0.293155	-0.580221
H	1.968869	-2.179498	1.570961
H	2.802941	-0.002785	-0.407780
H	2.915763	-1.736724	-0.673629
H	0.888454	-0.758000	-1.781159
H	0.458527	-1.963619	-0.583049
H	0.402859	1.071883	-0.196802
H	-0.515630	0.651883	2.055901
H	-0.419281	-1.082155	1.815875
H	1.946998	0.856729	1.931400
H	1.515240	-0.330363	3.153855
H	-2.303788	0.760742	0.084279
H	-1.636151	0.091034	-1.739728
C	4.433724	-2.152266	2.462614
H	4.327363	-0.206371	1.709245
H	5.458982	-2.056448	2.802604
H	3.948897	-3.108712	2.636365

22

cyclohexane X= BH_2 and Y= NH_2 scf done: -316.744609

C	0.007397	0.011523	0.004308
C	0.014015	0.024019	1.539332
C	1.434337	0.026779	2.116703
C	2.249651	1.195101	1.530204
C	2.265873	1.184322	-0.005660

C	0.844515	1.155907	-0.605640
B	0.733881	1.354890	-2.143193
N	1.366249	0.000584	3.583693
H	1.920950	-0.910541	1.813820
H	1.810332	2.138564	1.884313
H	3.273700	1.160472	1.920688
H	2.817723	2.049867	-0.384824
H	2.813705	0.297549	-0.351264
H	0.349542	2.112196	-0.304571
H	-1.020854	0.060381	-0.366506
H	0.412362	-0.947276	-0.345511
H	-0.510942	0.922144	1.896439
H	-0.531233	-0.836758	1.936931
H	-0.243111	1.001923	-2.734560
H	1.584353	1.940350	-2.745679
H	2.296986	-0.029045	3.989309
H	0.917136	0.842766	3.934220
21			
**cyclohexane X= BH_2 and Y= OH** scf done: -336.620160			
C	0.006332	0.003419	0.001455
C	0.017334	0.004377	1.536915
C	1.439374	-0.002772	2.088888
C	2.253396	1.170491	1.535567
C	2.266836	1.176424	-0.000714
C	0.843423	1.154230	-0.597349
B	0.731153	1.370053	-2.132679
O	1.338937	0.040281	3.517310
H	1.927734	-0.944061	1.789326
H	1.809904	2.101197	1.910211
H	3.279301	1.126561	1.922592
H	2.816117	2.048417	-0.367408
H	2.815966	0.296260	-0.360194
H	0.349532	2.106782	-0.284575
H	-1.023351	0.059172	-0.363156
H	0.406846	-0.952058	-0.362132
H	-0.489814	0.900295	1.915392
H	-0.525917	-0.859396	1.931319
H	-0.246792	1.024445	-2.726274
H	1.581914	1.960725	-2.729360
H	2.227318	0.045290	3.888731
21			
**cyclohexane X= BH_2 and Y= SH** scf done: -659.591512			
C	-0.007433	0.009090	-0.044949
C	-0.017519	0.008272	1.492098
C	1.408279	-0.000547	2.057736
C	2.228456	1.174214	1.515334
C	2.251515	1.178680	-0.022456
C	0.834532	1.160666	-0.632388
B	0.737071	1.377381	-2.169095
S	1.287529	0.007117	3.908861
H	1.890308	-0.942425	1.777323

H 1.789599 2.109640 1.882030
H 3.250668 1.131514 1.905471
H 2.805354 2.051675 -0.380399
H 2.803317 0.298430 -0.377407
H 0.338751 2.112841 -0.321920
H -1.033926 0.063563 -0.419329
H 0.398764 -0.946629 -0.400773
H -0.538886 0.903756 1.853342
H -0.573954 -0.856671 1.865694
H -0.231998 1.026023 -2.773712
H 1.590950 1.973620 -2.755180
H 2.614167 -0.010084 4.153583

20
cyclohexane X= BH_2 and Y= O^- scf done: -336.013526
C 0.010654 -0.019514 -0.017476
C 0.004264 0.011168 1.518728
C 1.427637 -0.003653 2.175451
C 2.240990 1.170816 1.530024
C 2.274610 1.154251 -0.006043
C 0.859537 1.119386 -0.625882
B 0.721293 1.400717 -2.134160
O 1.404644 0.027702 3.509350
H 1.915936 -0.941292 1.745170
H 1.787859 2.108144 1.886557
H 3.256525 1.135108 1.941792
H 2.831783 2.012791 -0.405767
H 2.813511 0.256909 -0.343074
H 0.359401 2.081044 -0.317886
H -1.008862 0.021579 -0.425162
H 0.436120 -0.975668 -0.355079
H -0.503460 0.920193 1.874986
H -0.555048 -0.841031 1.922544
H -0.270376 1.090701 -2.737543
H 1.550921 2.034938 -2.728357

20
cyclohexane X= BH_2 and Y= S^- scf done: -659.024173
C 0.009992 -0.012098 -0.043542
C 0.013379 0.003988 1.496291
C 1.428061 -0.003764 2.102191
C 2.241779 1.159319 1.507550
C 2.269140 1.159175 -0.032128
C 0.852632 1.132905 -0.646276
B 0.720404 1.402693 -2.159794
S 1.407723 0.017503 3.945178
H 1.914220 -0.938332 1.780407
H 1.803322 2.099324 1.869456
H 3.259571 1.121484 1.907672
H 2.823029 2.028291 -0.410113
H 2.813917 0.271079 -0.384007
H 0.355252 2.089137 -0.326756
H -1.016447 0.037685 -0.429511

H	0.424634	-0.967663	-0.396078
H	-0.505033	0.902540	1.857794
H	-0.545403	-0.851235	1.888448
H	-0.266874	1.083948	-2.763903
H	1.554636	2.028312	-2.754706

22

cyclohexane X= BH_2 and Y= $BH2^{\cdot-}$ scf done: -286.818830

C	0.121032	-0.128813	0.095812
C	-0.029273	-0.128235	1.638649
H	0.671751	0.786586	-0.173984
C	-0.831707	1.082664	2.165623
H	-0.534682	-1.054969	1.944254
H	0.963250	-0.151354	2.109259
C	-2.240466	1.197813	1.529031
H	-0.921071	1.023664	3.259075
H	-0.272330	2.004493	1.953468
C	-2.090171	1.197225	-0.013855
H	-2.791062	0.282382	1.798935
B	-3.021887	2.473636	2.047327
C	-1.287665	-0.013578	-0.540865
H	-3.082715	1.220257	-0.484414
H	-1.584881	2.124056	-0.319349
H	-1.847047	-0.935436	-0.328908
H	-1.198137	0.045563	-1.634299
B	0.902042	-1.405067	-0.422257
H	1.944064	-1.324131	-1.019893
H	0.445129	-2.501809	-0.216210
H	-4.063934	2.391880	2.644833
H	-2.565907	3.570725	1.841102

12

$[B_5CH_6]^-$ scf done: -166.100989

B	0.028339	0.087557	-0.111453
B	-0.083451	-0.002747	1.603622
C	1.323714	0.309478	0.847907
B	0.699619	1.522826	1.734917
B	-0.726216	1.367548	0.770500
B	0.811003	1.613640	0.020691
H	1.386814	2.325222	-0.741352
H	1.166877	2.146665	2.635376
H	2.285147	-0.186593	0.884103
H	-1.785627	1.915692	0.731566
H	-0.376745	-0.860542	2.376347
H	-0.156128	-0.682521	-1.001055

16

$[B_5CH_6]^-$ X= CH= CH_2 scf done: -243.521559

C	0.162779	-0.180480	-0.172655
C	-0.010258	-0.006472	1.274111
B	1.523549	-0.168396	-1.086989
B	0.446931	-1.507839	-1.087373
B	-0.868653	-0.444298	-1.408695
B	0.509866	-0.447115	-2.448632

B	0.212306	0.895999	-1.404796
H	0.536099	-2.649015	-0.765146
H	-2.052782	-0.561105	-1.417482
H	0.680869	-0.585020	-3.619973
H	0.073629	2.077001	-1.388799
H	2.655870	-0.013351	-0.752440
C	-1.166760	-0.030460	1.948173
H	0.907177	0.157789	1.841369
H	-1.195306	0.108661	3.024907
H	-2.111940	-0.189915	1.438911

22

$[B_5CH_6]^-$ X= Ph scf done: -397.218409

C	0.009348	-0.012602	0.013654
C	0.004596	-0.005578	1.488979
B	1.226777	-0.010196	-1.079146
B	0.019910	-1.236245	-1.073592
B	-1.201637	-0.025000	-1.086233
B	0.016108	-0.023109	-2.302610
B	0.005231	1.201119	-1.084665
H	0.026781	-2.411473	-0.896302
H	-2.381222	-0.031090	-0.930297
H	0.019635	-0.028237	-3.493644
H	-0.002919	2.377979	-0.918495
H	2.405378	-0.002720	-0.916049
C	-1.198759	-0.003919	2.215310
C	-1.204389	0.002795	3.608497
C	-0.004377	0.008120	4.321132
C	1.200142	0.006477	3.616123
C	1.203318	-0.000269	2.222928
H	-2.133616	-0.008039	1.666323
H	-2.151117	0.003932	4.141148
H	-0.007838	0.013402	5.406596
H	2.143473	0.010512	4.154752
H	2.141637	-0.001448	1.679852

12

$[B_5CH_6]^-$ X= O^- scf done: -240.613777

B	0.077876	0.064626	-0.108288
B	-0.026837	-0.030794	1.602933
B	1.512646	0.203906	0.848922
B	0.762876	1.487231	1.735675
C	-0.611689	1.315192	0.780399
B	0.867993	1.582601	0.025395
H	1.182493	2.442568	-0.754568
H	0.973682	2.253341	2.638653
H	2.577002	-0.351986	0.883544
O	-1.763976	1.918359	0.743580
H	-0.594095	-0.758982	2.374274

H	-0.384625	-0.569838	-1.019349

12

$[B_5CH_6]^-$ X= S^- scf done: -563.630374

B	0.115321	0.045535	-0.105329
B	0.001981	-0.044309	1.603991
B	1.543640	0.188690	0.857792
B	0.789125	1.472815	1.735926
C	-0.536724	1.275382	0.776403
B	0.903294	1.562990	0.027571
H	1.211922	2.418810	-0.748448
H	0.985846	2.240877	2.630998
H	2.609275	-0.367188	0.900331
S	-2.132077	2.108591	0.713744
H	-0.572279	-0.761725	2.369050
H	-0.345978	-0.584244	-1.010859

13

$[B_5CH_6]^-$ X= SH scf done: -564.321605

B	0.178743	0.001744	-0.165737
B	-0.047538	-0.089776	1.536167
B	1.522924	0.209981	0.890703
B	0.684531	1.469345	1.717584
C	-0.536428	1.201477	0.674821
B	0.910152	1.561072	0.015569
H	1.213677	2.426934	-0.738958
H	0.767824	2.247204	2.612584
H	2.591412	-0.303921	1.006447
S	-2.141838	1.979993	0.445510
H	-0.663889	-0.804763	2.258453
H	-0.218132	-0.625040	-1.093457
H	-2.514829	2.003144	1.742875

13

$[B_5CH_6]^-$ X= OH scf done: -241.339283

B	0.086149	0.033820	-0.139325
B	-0.083831	-0.044099	1.570890
B	1.467814	0.227796	0.864564
B	0.690038	1.494108	1.725608
C	-0.575825	1.246965	0.725546
B	0.830767	1.590018	0.011728
H	1.124306	2.449755	-0.755033
H	0.818782	2.272902	2.616750
H	2.531938	-0.300992	0.934190
O	-1.828774	1.878328	0.616431
H	-0.693709	-0.744942	2.317376
H	-0.354126	-0.590197	-1.050451
H	-2.266920	1.763933	1.464285

14

$[B_5CH_6]^-$ X= BH_2 scf done: -191.552463

B	0.050063	0.046560	-0.068311
B	-0.002467	-0.023876	1.626269
B	1.517886	0.189079	0.832581

B 0.828228 1.504844 1.715653
C -0.538373 1.307409 0.815374
B 0.880803 1.575447 0.021034
H 1.160731 2.406797 -0.782488
H 1.055423 2.266061 2.601119
H 2.565817 -0.380896 0.841376
B -1.867436 2.029913 0.804396
H -0.558819 -0.705139 2.427242
H -0.453873 -0.563916 -0.956330
H -2.910257 1.432343 0.748082
H -1.933778 3.230375 0.851700

14
$[B_5CH_6]^-$ X= NH_2 scf done: -221.466301
B 0.097229 0.048891 -0.140333
B -0.096656 -0.023971 1.568880
B 1.471383 0.231704 0.862819
B 0.709979 1.487546 1.737467
C -0.586639 1.265039 0.738296
B 0.814939 1.605300 0.023162
H 1.099929 2.477921 -0.736883
H 0.844193 2.262358 2.633175
H 2.533463 -0.304626 0.923919
N -1.883026 1.892399 0.635022
H -0.697970 -0.735546 2.312167
H -0.347465 -0.569149 -1.053952
H -2.389786 1.789835 1.508376
H -1.775625 2.887300 0.465583

29
$[B_5CH_6]^-$ X= O–$[NMe_4]^+$ scf done: -455.100838
B 0.361851 0.310542 -0.437574
B 0.120612 -0.352093 1.133542
B 1.673211 0.211557 0.674227
B 1.005045 1.742288 0.282148
C 0.738157 1.061945 1.756339
O 0.963186 1.468186 3.018599
B -0.553500 1.176275 0.742959
C 2.215298 -1.276880 3.884271
N 2.221974 -0.387251 5.102350
C 2.809141 -1.113789 6.261333
C 0.796649 0.031530 5.395196
C 3.026829 0.854224 4.781170
H 0.166458 -0.060848 -1.554601
H 1.462574 2.808186 0.000293
H -1.617897 1.689698 0.911633
H -0.290753 -1.322209 1.706127
H 2.792120 -0.202894 0.793891
H 3.243958 -1.559070 3.658791
H 1.616178 -2.160186 4.106318
H 1.780658 -0.720026 3.054012
H 3.832492 -1.405197 6.021561
H 2.807490 -0.458346 7.132199

H	2.211594	-2.002463	6.468094
H	4.038677	0.542369	4.517409
H	2.477260	1.336568	3.932902
H	3.042659	1.482525	5.674031
H	0.809239	0.654128	6.292131
H	0.477329	0.603096	4.485823
H	0.207390	-0.870719	5.566128

29

$[B_5CH_6]^-$ X= S–$[NMe_4]^+$ scf done: -778.098673

B	-0.058387	-0.125726	-0.190296
B	0.047113	-0.175108	1.526994
B	1.476729	-0.041541	0.591109
B	0.836129	1.346392	-0.186522
C	0.895260	1.237170	1.447902
S	1.634568	2.332742	2.683000
B	-0.594587	1.213366	0.758907
C	2.707510	-0.776522	3.725609
N	2.278880	-0.344444	5.112976
C	2.477962	-1.458714	6.080667
C	0.822538	0.065421	5.084380
C	3.103070	0.853257	5.503028
H	-0.546282	-0.825780	-1.025528
H	1.282932	2.175635	-0.912546
H	-1.538184	1.912968	0.949284
H	-0.284185	-0.838352	2.465432
H	2.552328	-0.561255	0.620614
H	3.739947	-1.124543	3.787527
H	2.041700	-1.563638	3.379323
H	2.615656	0.104297	3.079069
H	3.533045	-1.735123	6.096770
H	2.163576	-1.133233	7.073044
H	1.877400	-2.310667	5.762223
H	4.152985	0.558227	5.526542
H	2.917917	1.610545	4.727056
H	2.779674	1.194729	6.487261
H	0.538166	0.353962	6.098406
H	0.749404	0.914859	4.378562
H	0.233013	-0.777775	4.730509

14

$[B_5CH_6]^-$ X= $BH_2^{\cdot-}$ scf done: -191.425857

B	0.058187	0.046537	-0.067282
B	0.002180	-0.019056	1.632055
B	1.525031	0.184041	0.838361
B	0.828083	1.499824	1.717952
C	-0.539278	1.308173	0.813496
B	0.884355	1.565269	0.018585
H	1.169799	2.388877	-0.793861

H 1.057329 2.257734 2.608365
H 2.577691 -0.389188 0.851039
B -1.890699 2.044047 0.797281
H -0.549831 -0.697462 2.441329
H -0.437467 -0.566307 -0.960897
H -2.937089 1.442748 0.741728
H -1.954342 3.249764 0.839546

22
$[B_5CH_6]^-$ X= Ph$^{\cdot-}$ scf done: -410.395280
C 0.032586 0.400425 0.059740
C -0.001333 0.000240 1.401117
C 1.190318 -0.400074 2.017809
C 2.388637 -0.400008 1.308438
C 2.413295 -0.000051 -0.026682
C 1.231590 0.400065 -0.648457
C -1.275101 0.000347 2.154310
B -2.775525 -0.522565 1.774625
B -1.954163 -1.090888 3.161927
B -1.665703 0.522648 3.652006
C -3.149260 0.000048 3.262300
B -2.485582 1.091070 2.263597
H -2.739461 2.151404 1.826571
H -1.123913 1.032171 4.561203
H -4.077196 -0.000093 3.810978
H -1.693293 -2.151135 3.595030
H -3.310563 -1.031883 0.861322
H 1.175465 -0.712110 3.055994
H 3.303439 -0.712641 1.799283
H 3.346428 -0.000173 -0.578461
H 1.242354 0.712586 -1.686604
H -0.884219 0.712583 -0.427544

24
$[CB_{11}H_{11}]^-$ scf done: -319.057276
C 0.000251 0.000179 -0.057957
H -0.000791 0.000432 1.023102
B 1.515875 -0.001497 -0.843433
B 0.470833 1.439955 -0.844500
B -1.223346 0.891735 -0.846045
B -0.466226 1.447758 -2.350403
B 0.003328 -0.000184 -3.284197
H 0.004461 -0.000379 -4.474807
B 0.467480 -1.440671 -0.844008
B 1.232702 -0.895948 -2.348630
B 1.234480 0.893166 -2.348879
B -1.225549 -0.888839 -0.845912
B -0.469316 -1.446864 -2.350098
B -1.519491 0.001632 -2.351451

H	2.103110	-1.529688	-2.854995
H	-0.797135	2.471639	-2.858181
H	2.106234	1.524771	-2.855616
H	-2.024045	1.474674	-0.191691
H	-2.595187	0.002749	-2.859891
H	0.777127	2.381191	-0.189235
H	2.504979	-0.002639	-0.187107
H	0.771713	-2.382389	-0.188468
H	-0.802391	-2.470272	-2.857417
H	-2.027369	-1.469802	-0.191195
28			
**$[CB_{11}H_{11}]^-$ X= CH= CH_2** scf done: -396.468157			
C	0.017152	-0.025346	0.050023
B	0.037507	0.005597	1.771856
B	1.750771	0.018724	2.218648
B	0.813205	1.540023	2.201215
B	1.198549	2.409327	0.687928
B	2.372600	1.425106	-0.230476
B	2.475122	1.507774	1.548814
B	2.713216	-0.053078	0.714669
B	1.205970	-0.974048	0.857506
B	1.587682	-0.108543	-0.654299
B	0.657711	1.405894	-0.665068
B	-0.305449	1.478444	0.826776
C	-1.108307	-0.687671	-0.678785
H	3.371794	2.064764	2.098064
H	1.170396	3.596221	0.614284
H	3.760967	-0.613442	0.658429
H	3.178776	1.912732	-0.956587
H	1.094748	-2.153986	0.835254
H	2.118561	-0.486963	3.230573
H	1.717882	-0.722499	-1.660785
H	0.171890	1.776712	-1.683162
H	-1.412728	1.901985	0.788400
H	0.511725	2.110796	3.200346
H	-0.847477	-0.525679	2.354659
C	-1.748087	-1.795848	-0.310556
H	-1.405617	-0.184993	-1.595854
H	-2.555935	-2.195032	-0.914990
H	-1.489614	-2.333374	0.594382
34			
**$[CB_{11}H_{11}]^-$ X= Ph** scf done: -550.159909			
C	0.007139	0.022802	-0.014443
B	0.002125	0.018438	1.713050
B	1.704752	-0.016086	2.197178
B	0.797340	1.522425	2.199406
B	1.230749	2.418326	0.719505
B	2.404956	1.433918	-0.199005
B	2.472255	1.472001	1.583382
B	2.697158	-0.070533	0.713110
B	1.170775	-0.961380	0.798668

B	1.599464	-0.071263	-0.682031
B	0.701314	1.463075	-0.670786
B	-0.295513	1.517399	0.800403
C	-1.132655	-0.667404	-0.731820
H	3.367709	1.999107	2.163142
H	1.226640	3.606521	0.669745
H	3.732777	-0.653224	0.665157
H	3.231518	1.925842	-0.898365
H	1.046092	-2.138803	0.749548
H	2.035994	-0.558871	3.202224
H	1.742140	-0.662391	-1.699649
H	0.286842	1.914084	-1.683745
H	-1.392046	1.965325	0.752225
H	0.483702	2.071028	3.206871
H	-0.900977	-0.506330	2.272784
C	-1.704088	-1.836365	-0.209126
C	-2.753582	-2.481616	-0.859202
C	-3.263022	-1.975223	-2.053547
C	-2.705597	-0.814515	-2.585283
C	-1.655528	-0.170681	-1.932920
H	-1.320586	-2.242676	0.716872
H	-3.173273	-3.384770	-0.427478
H	-4.080567	-2.476966	-2.560933
H	-3.087418	-0.403244	-3.514439
H	-1.234788	0.729216	-2.359790

26

$[CB_{11}H_{11}]^-$ X= NH_2 scf done: -374.419269

C	-0.012575	0.067082	0.014872
N	-0.026513	0.133449	1.464037
B	1.493777	0.041795	-0.787009
B	0.457577	1.489421	-0.838850
B	-1.227280	0.939832	-0.830143
B	-0.466620	1.450572	-2.350471
B	0.006924	-0.018053	-3.242077
H	0.013638	-0.047101	-4.431622
B	0.459218	-1.402371	-0.775717
B	1.230322	-0.890545	-2.278986
B	1.231662	0.899014	-2.316995
B	-1.222707	-0.842218	-0.784732
B	-0.468780	-1.441120	-2.278676
B	-1.519355	0.007275	-2.309128
H	2.109850	-1.531347	-2.759797
H	-0.797221	2.460144	-2.885620
H	2.110872	1.514707	-2.829821
H	-2.031909	1.531809	-0.191934
H	-2.595482	-0.006547	-2.815999
H	0.762922	2.440751	-0.202201
H	2.475297	0.054965	-0.116039
H	0.758898	-2.319195	-0.081831
H	-0.805849	-2.476421	-2.758190
H	-2.023095	-1.403120	-0.107052
H	-0.737004	-0.493567	1.825385

H	0.868805	-0.175146	1.826842

25

$[CB_{11}H_{11}]^-$ X= OH scf done: -394.291929

C	0.005525	0.016323	-0.006282
O	-0.019526	0.024807	1.399369
B	1.510379	0.012167	-0.808048
B	0.472864	1.458724	-0.825183
B	-1.215065	0.903686	-0.809532
B	-0.461632	1.448600	-2.325166
B	0.008933	-0.003436	-3.247771
H	0.008322	-0.010696	-4.437470
B	0.474440	-1.435535	-0.807556
B	1.239825	-0.892576	-2.307804
B	1.238890	0.898332	-2.318673
B	-1.214092	-0.881929	-0.798523
B	-0.460133	-1.444556	-2.307453
B	-1.511413	0.001549	-2.306071
H	2.115997	-1.524583	-2.805297
H	-0.797694	2.467705	-2.837859
H	2.114457	1.525133	-2.823771
H	-2.009363	1.483580	-0.149323
H	-2.588367	-0.002177	-2.810473
H	0.780730	2.391254	-0.159843
H	2.485173	0.016893	-0.123829
H	0.783142	-2.359592	-0.130919
H	-0.795213	-2.470170	-2.807660
H	-2.007855	-1.454505	-0.131320
H	0.896727	0.030249	1.694519

24

$[CB_{11}H_{11}]^-$ X= O^- scf done: -393.608252

C	0.000171	0.000115	0.130476
O	-0.001121	0.000781	1.414034
B	1.510050	-0.001879	-0.814024
B	0.468799	1.433777	-0.815489
B	-1.218484	0.887701	-0.816909
B	-0.464211	1.441193	-2.327646
B	0.003612	-0.001403	-3.273609
H	0.004786	-0.002076	-4.471469
B	0.465591	-1.435011	-0.814017
B	1.228150	-0.893331	-2.325071
B	1.230023	0.888792	-2.325801
B	-1.220540	-0.885731	-0.816302
B	-0.467287	-1.442155	-2.326383
B	-1.513347	0.000734	-2.328133
H	2.102772	-1.530797	-2.841314
H	-0.796656	2.470087	-2.846017
H	2.106043	1.523563	-2.843004
H	-2.045934	1.490328	-0.204713
H	-2.594373	0.001867	-2.846992
H	0.785438	2.406717	-0.202485
H	2.532224	-0.002878	-0.199419

H 0.779978 -2.408171 -0.200181
H -0.801927 -2.470955 -2.843521
H -2.049166 -1.485893 -0.203290

25
$[CB_{11}H_{11}]^-$ X= SH scf done: -717.266264
C -0.000525 -0.042081 -0.030320
S -0.054236 -0.106454 1.792891
B 1.515816 -0.046385 -0.832475
B 0.475126 1.408999 -0.811771
B -1.222526 0.877632 -0.832422
B -0.454150 1.443976 -2.323113
B 0.006859 0.009420 -3.276150
H 0.010034 0.028576 -4.465658
B 0.463565 -1.480380 -0.865990
B 1.227865 -0.908243 -2.356514
B 1.241397 0.878518 -2.319074
B -1.226945 -0.907417 -0.846334
B -0.474847 -1.447618 -2.363418
B -1.515235 0.007290 -2.342717
H 2.098193 -1.537625 -2.866807
H -0.774862 2.480532 -2.809590
H 2.121580 1.514880 -2.803472
H -2.017170 1.451150 -0.168406
H -2.592354 0.018243 -2.846192
H 0.789009 2.334781 -0.140382
H 2.502279 -0.061153 -0.174431
H 0.758043 -2.430212 -0.223805
H -0.819613 -2.460826 -2.881431
H -2.032645 -1.492776 -0.202831
H 1.030391 0.666421 1.998477

24
$[CB_{11}H_{11}]^-$ X= S^- scf done: -716.605870
C 0.000297 0.000133 0.036389
S -0.001449 0.001130 1.851923
B 1.508231 -0.001981 -0.831078
B 0.468290 1.432012 -0.832465
B -1.216961 0.886612 -0.833966
B -0.463879 1.440333 -2.347499
B 0.003592 -0.001381 -3.290767
H 0.004812 -0.002007 -4.487532
B 0.465115 -1.433349 -0.830874
B 1.227201 -0.892730 -2.344685
B 1.229167 0.888115 -2.345672
B -1.219069 -0.884669 -0.833211
B -0.467050 -1.441094 -2.345984
B -1.512179 0.000736 -2.347998
H 2.103018 -1.530857 -2.852155
H -0.796803 2.470480 -2.857123
H 2.106429 1.523649 -2.853908
H -2.037536 1.484027 -0.219282
H -2.594690 0.001729 -2.857877

H 0.782176 2.396766 -0.216969
H 2.521666 -0.002966 -0.213851
H 0.776880 -2.398371 -0.214689
H -0.802080 -2.471167 -2.854428
H -2.040582 -1.479777 -0.217579

26
$[CB_{11}H_{11}]^-$ X= BH_2 scf done: -344.496532
C 0.000142 0.000508 -0.009282
B -0.000551 0.000125 1.537401
B 1.520120 0.001891 -0.836162
B 0.478954 1.436580 -0.826080
B -1.229628 0.884067 -0.830692
B -0.466847 1.447141 -2.326838
B 0.003444 -0.001393 -3.268414
H 0.004504 -0.002049 -4.458746
B 0.469107 -1.438511 -0.820557
B 1.234827 -0.894223 -2.331590
B 1.235731 0.889942 -2.338511
B -1.232024 -0.885480 -0.836254
B -0.471804 -1.447245 -2.329551
B -1.518556 -0.000673 -2.342535
H 2.103138 -1.533347 -2.833929
H -0.797782 2.474465 -2.826809
H 2.104696 1.524346 -2.845631
H -2.033369 1.474618 -0.186908
H -2.593164 0.002450 -2.852347
H 0.784372 2.387226 -0.183443
H 2.511850 -0.000585 -0.185422
H 0.775719 -2.390157 -0.179260
H -0.801184 -2.474452 -2.830733
H -2.034944 -1.470818 -0.187984
H -1.006978 -0.213039 2.143925
H 1.005596 0.212678 2.144600

41
$[CB_{11}H_{11}]^-$ X= S–$[NMe_4]^+$ scf done: -931.050718
B 0.127356 -0.086517 -0.053511
B 0.102214 0.024406 1.727736
B 1.653688 0.092202 0.852185
B 1.346124 1.188353 2.215203
B -0.352922 1.687638 2.164889
B -1.112080 0.904310 0.763869
B -0.312374 1.515835 -0.707506
B -0.607633 2.605347 0.665965
C 0.888618 2.710134 1.526407
S 1.274154 4.267556 2.399058
B 1.396150 1.014047 -0.654035
B 0.936208 2.673186 -0.205926

B	2.137068	1.793254	0.750563
C	4.601546	4.619029	1.613987
N	4.906543	4.307853	3.062869
C	4.356073	2.942557	3.413550
C	4.214867	5.326009	3.931075
C	6.378764	4.343045	3.293440
H	-0.184397	-0.888994	2.437285
H	2.476305	-0.767686	0.939294
H	-2.267174	0.614268	0.784538
H	1.250911	3.660126	-0.783597
H	-0.896687	1.662116	-1.735277
H	-1.325427	3.547011	0.665914
H	3.259532	2.182468	0.789477
H	2.035566	0.808358	-1.639206
H	-0.147586	-1.096179	-0.623905
H	-0.898032	2.018113	3.163040
H	1.927188	1.179234	3.252976
H	4.567413	6.319476	3.653155
H	3.140526	5.213381	3.746693
H	4.456912	5.112532	4.972609
H	4.790200	2.205409	2.741484
H	4.618556	2.731018	4.451011
H	3.268889	2.994735	3.277120
H	5.061040	3.852124	0.993067
H	3.502085	4.602854	1.509714
H	5.015434	5.603521	1.389360
H	6.852527	3.595408	2.658082
H	6.756909	5.334785	3.043563
H	6.582567	4.119961	4.341116
41<br>**$[CB_{11}H_{11}]^-$ X= O–$[NMe_4]^+$** scf done: -608.067945			
B	-0.152837	0.111894	-0.093551
B	-0.075648	0.060623	1.690226
B	1.408427	-0.168020	0.726092
B	1.435177	0.839203	2.187640
B	-0.090487	1.736173	2.284174
B	-1.077087	1.293092	0.877081
B	-0.212939	1.826751	-0.590091
B	-0.175041	2.821822	0.882461
C	1.343796	2.506260	1.669930
O	1.965571	3.455455	2.368221
B	1.323410	0.924088	-0.684176
B	1.299288	2.591802	-0.074828
B	2.290244	1.366315	0.730515
C	4.513806	4.477260	1.524854
N	4.770795	4.366822	3.012245
C	4.595606	2.925189	3.436765
C	3.741760	5.202083	3.735688
C	6.145791	4.838199	3.336406
H	-0.553140	-0.821247	2.335684
H	1.989987	-1.209304	0.689217
H	-2.267673	1.284786	0.942425

H 1.823951 3.532415 -0.586128
H -0.788243 2.199979 -1.565783
H -0.637360 3.909978 1.002775
H 3.478295 1.488622 0.753496
H 1.844308 0.659780 -1.724625
H -0.692656 -0.744087 -0.723930
H -0.496065 2.099450 3.340616
H 2.050165 0.611805 3.183776
H 3.860205 6.238824 3.417772
H 2.759171 4.800930 3.457310
H 3.925317 5.114879 4.807470
H 5.290196 2.310129 2.866046
H 4.810007 2.860131 4.504613
H 3.549170 2.676647 3.202766
H 5.211606 3.821848 1.005113
H 3.469612 4.152416 1.385020
H 4.670555 5.516906 1.232954
H 6.868419 4.221038 2.803237
H 6.251162 5.878413 3.027050
H 6.310999 4.752935 4.410806

26
$[CB_{11}H_{11}]^-$ X= $BH_2^{\cdot-}$ scf done: -344.394897
B -0.043444 -0.139328 -0.082656
B -0.016549 -0.029426 1.698405
B 1.514466 -0.021995 0.785022
B 2.073065 1.660245 0.677477
B 0.879505 2.584029 -0.246836
B -0.431201 1.483939 -0.721715
B 1.255752 0.908454 -0.713014
B -1.218207 0.900625 0.767904
B -0.389031 1.648581 2.150682
C 0.885012 2.634803 1.491195
B 1.363463 3.980799 2.218904
B -0.635102 2.580962 0.666665
B 1.290470 1.090507 2.157869
H 1.853137 0.670750 -1.721950
H -1.040992 1.652839 -1.737025
H 2.293302 -0.928113 0.845131
H -0.910082 1.986314 3.161152
H -0.326121 -0.939766 2.409504
H 1.899463 1.041954 3.177124
H -1.332088 3.543397 0.674556
H -2.388009 0.656190 0.812833
H -0.377947 -1.139245 -0.650095
H 3.210267 2.004165 0.693839
H 1.212953 3.550149 -0.852828
H 2.304228 3.942883 2.967542
H 0.893663 5.034024 1.876913

34
$[CB_{11}H_{11}]^-$ X= $Ph^{\cdot-}$ scf done: -550.053769
C 0.003820 0.020203 -0.016010

B	0.002648	0.018070	1.713191
B	1.706567	-0.014087	2.197594
B	0.800404	1.522908	2.199316
B	1.232477	2.417846	0.720011
B	2.405327	1.434521	-0.197298
B	2.474737	1.473915	1.584563
B	2.697858	-0.068146	0.715098
B	1.170641	-0.961237	0.799472
B	1.599162	-0.070919	-0.679712
B	0.700190	1.461649	-0.670990
B	-0.293688	1.516055	0.800839
C	-1.137914	-0.670353	-0.735467
H	3.372891	2.002864	2.165863
H	1.230829	3.608555	0.671419
H	3.737565	-0.649092	0.668669
H	3.234922	1.928433	-0.895851
H	1.053707	-2.139017	0.755326
H	2.040897	-0.555261	3.205049
H	1.749627	-0.661205	-1.696805
H	0.291089	1.917799	-1.682829
H	-1.388179	1.969682	0.757531
H	0.489532	2.074416	3.209181
H	-0.896390	-0.503975	2.280631
C	-1.708591	-1.839712	-0.211959
C	-2.758368	-2.484683	-0.862587
C	-3.264458	-1.975497	-2.054951
C	-2.710094	-0.816247	-2.588457
C	-1.659830	-0.171486	-1.936932
H	-1.329338	-2.252346	0.714892
H	-3.182028	-3.390299	-0.433734
H	-4.084805	-2.478651	-2.564304
H	-3.095690	-0.406832	-3.519883
H	-1.243720	0.729299	-2.370169

12
cyclobutane scf done: -157.257147

C	-0.012652	0.113022	0.166124
H	0.005139	-0.125993	1.232640
C	1.419968	0.150845	-0.435745
H	-0.649517	0.987627	0.018116
C	-0.300252	-1.146324	-0.697804
H	-0.784071	-0.884460	-1.642438
H	-0.860494	-1.962088	-0.236164
C	1.233421	-1.329223	-0.871777
H	1.476625	0.820757	-1.297607
H	2.248161	0.381098	0.237542
H	1.601753	-1.619850	-1.857691
H	1.642703	-2.020806	-0.130752

14
cyclobutane X= BH_2 scf done: -182.690419

C	-0.061672	-0.075363	-0.017174
C	-0.026484	0.002247	1.534206
C	1.535118	-0.004099	1.459891
C	1.436566	-0.486881	-0.023702
H	-0.215020	0.907859	-0.469855
H	-0.770443	-0.775925	-0.462789
H	-0.430375	-0.898942	2.001952
H	-0.495936	0.872382	1.998262
H	1.567688	-1.566925	-0.125462
H	2.084357	0.009704	-0.749410
H	2.003549	-0.750459	2.134331
B	2.361197	1.246583	1.842699
H	2.021049	1.946407	2.750589
H	3.393822	1.487023	1.289862

16

cyclobutane X= CH= CH_2 scf done: -234.674214

C	-0.137732	-0.017417	0.211880
C	0.144238	-0.144461	1.743705
C	1.658879	0.013923	1.448179
C	1.365683	-0.371678	-0.025569
H	-0.317252	1.030928	-0.046693
C	-1.188275	-0.879366	-0.402907
H	-0.093153	-1.149591	2.103938
H	-0.335868	0.583424	2.400833
H	1.493606	-1.444753	-0.194537
H	1.886208	0.170099	-0.817828
H	2.353166	-0.615092	2.008289
H	1.982626	1.053818	1.538151
C	-2.199703	-0.449764	-1.157035
H	-1.097226	-1.947399	-0.202332
H	-2.930979	-1.133153	-1.574134
H	-2.330857	0.604419	-1.383313

14

cyclobutane X= NH_2 scf done: -212.625826

C	-0.015619	-0.017423	0.002261
C	0.017941	0.017707	1.554974
C	1.571055	-0.012411	1.499864
C	1.470488	-0.469370	0.017612
H	-0.135748	0.977713	-0.426609
H	-0.735371	-0.693005	-0.463689
H	-0.389017	-0.893563	2.000080
H	-0.427930	0.884373	2.047970
H	1.576472	-1.552578	-0.080547
H	2.151874	0.019467	-0.682203
H	2.050242	-0.730383	2.180122
N	2.126178	1.338838	1.596679
H	2.024644	1.705116	2.538707
H	3.118208	1.339131	1.378178

13

cyclobutane X= OH scf done: -232.499949

C	-0.009246	-0.014835	0.004982
C	-0.002983	0.026228	1.559254
C	1.551411	-0.019256	1.529298
C	1.485221	-0.443219	0.045320
H	-0.144784	0.972961	-0.437332
H	-0.709102	-0.707849	-0.464866
H	-0.428105	-0.877118	2.004828
H	-0.446525	0.900340	2.042020
H	1.619918	-1.519927	-0.080181
H	2.180178	0.083898	-0.609562
H	2.033308	-0.719311	2.221670
O	2.169165	1.263424	1.618065
H	2.061452	1.595186	2.515571

13

cyclobutane X= SH scf done: -555.472180

C	-0.038474	-0.049560	-0.020300
C	-0.010994	-0.001312	1.531481
C	1.541749	-0.062793	1.487257
C	1.447614	-0.501672	-0.002195
H	-0.160274	0.940944	-0.460933
H	-0.756625	-0.729307	-0.481243
H	-0.421975	-0.910013	1.981006
H	-0.460620	0.865437	2.017529
H	1.558438	-1.584249	-0.098382
H	2.129838	-0.021285	-0.706056
H	2.023417	-0.763826	2.168191
S	2.403064	1.559778	1.619062
H	2.104751	1.798380	2.913649

12

cyclobutane X= O^- scf done: -231.899545

C	-0.036911	-0.298405	0.058258
C	0.024135	0.282473	1.492171
C	1.567091	-0.158538	1.578479
C	1.505819	-0.198516	-0.026916
H	-0.627621	0.244254	-0.689732
H	-0.375307	-1.340743	0.058226
H	-0.674112	-0.103237	2.247404
H	-0.015823	1.377643	1.486013
H	2.057128	-0.989857	-0.552783
H	1.826778	0.779493	-0.403104
H	1.522825	-1.253211	1.881892
O	2.456112	0.561085	2.217749

12<br>**cyclobutane X= S**$^-$ scf done: -554.903426<br>C -0.029280 -0.051308 -0.012521<br>C -0.019375 -0.001675 1.538975<br>C 1.549745 -0.012423 1.515276<br>C 1.461596 -0.482465 0.020584<br>H -0.149246 0.944126 -0.444729<br>H -0.741610 -0.733685 -0.491225<br>H -0.437872 -0.917189 1.982193<br>H -0.471930 0.869921 2.014845<br>H 1.577002 -1.571306 -0.083592<br>H 2.143628 0.020796 -0.666802<br>H 1.988429 -0.764471 2.181281<br>S 2.359025 1.602118 1.793375
29<br>**cyclobutane X= O–[NMe$_4$]$^+$** scf done: -446.287572<br>O -0.812621 -0.971921 0.699772<br>C -0.309963 -0.342782 1.790400<br>C 1.242620 -0.143856 1.952184<br>C 0.962355 1.236567 2.601072<br>C -0.441867 1.217844 1.943051<br>C -0.510558 -2.573891 -1.664907<br>N -2.018385 -2.479185 -1.630923<br>C -2.613386 -3.222914 -2.779232<br>C -2.407794 -1.020155 -1.688143<br>C -2.504256 -3.052854 -0.319776<br>H 1.631569 2.062712 2.347547<br>H 0.901866 1.169048 3.691025<br>H 1.808818 -0.886809 2.523902<br>H 1.695102 -0.031562 0.960985<br>H -1.290713 1.618774 2.507083<br>H -0.420379 1.678576 0.949526<br>H -0.673897 -0.786598 2.745742<br>H -3.495710 -0.956660 -1.650033<br>H -1.932471 -0.547970 -0.812008<br>H -2.041358 -0.606807 -2.628306<br>H -3.591143 -2.968799 -0.293596<br>H -2.208219 -4.101462 -0.276699<br>H -2.017289 -2.446522 0.464968<br>H -3.699157 -3.141260 -2.734751<br>H -2.249391 -2.792397 -3.711902<br>H -2.320070 -4.270646 -2.717855<br>H -0.154205 -2.004472 -0.790111<br>H -0.234531 -3.627394 -1.610251<br>H -0.163751 -2.144311 -2.605240
29<br>**cyclobutane X=** S**–[NMe$_4$]$^+$** scf done: -769.270550

C	0.187573	-1.313644	0.427080
N	0.377639	-0.323866	1.552962
C	1.202156	-0.963928	2.645747
C	-0.949327	0.098618	2.093282
C	1.133586	0.869325	1.018383
S	3.635011	-1.456216	0.245725
C	4.770263	-0.189360	-0.417082
C	6.246786	-0.142672	0.075637
C	6.680938	0.226708	-1.369305
C	5.317418	-0.324660	-1.868808
H	7.588147	-0.241787	-1.759160
H	6.762375	1.308604	-1.509495
H	6.489041	0.552020	0.885095
H	6.580546	-1.144332	0.355623
H	4.794754	0.220232	-2.660437
H	5.384069	-1.378643	-2.147770
H	4.331124	0.810943	-0.300074
H	-0.438482	-0.847354	-0.333719
H	1.185658	-1.543810	0.027134
H	-0.301905	-2.200997	0.827715
H	0.541913	1.324345	0.224344
H	1.283800	1.575851	1.834356
H	2.088025	0.476402	0.639327
H	-1.528891	0.561016	1.294864
H	-1.474193	-0.778122	2.472117
H	-0.794406	0.814655	2.899772
H	2.184712	-1.202245	2.211827
H	1.299514	-0.247925	3.461887
H	0.683655	-1.859161	2.988076

22

cyclobutane X= Ph scf done: -388.365692

C	-0.081511	0.136873	0.076932
C	0.147789	0.326752	1.600037
C	1.625810	-0.119328	1.390109
C	1.193504	-0.746630	0.030985
H	0.052200	1.071439	-0.473242
H	-1.021491	-0.320164	-0.236869
H	-0.406944	-0.411727	2.184919
H	-0.030697	1.317434	2.023009
H	0.951947	-1.806619	0.145946
H	1.865191	-0.628840	-0.821770
C	2.321043	-0.961914	2.429839
H	2.236890	0.769562	1.189353
C	2.084854	-0.759109	3.794760
C	2.750537	-1.514743	4.758423
C	3.667142	-2.491778	4.373444
C	3.910796	-2.705350	3.017797
C	3.242905	-1.947422	2.057352
H	1.371568	-0.003682	4.107628
H	2.550989	-1.341952	5.810485

H 4.183447 -3.082464 5.121777
H 4.619835 -3.464877 2.706525
H 3.439579 -2.125733 1.005293

22
cyclobutane X= Ph$^{\cdot -}$ scf done: -388.332680
C -0.083389 0.101832 0.062698
C 0.100013 0.262580 1.595728
C 1.602818 -0.119969 1.417089
C 1.252217 -0.687309 0.010755
H 0.000571 1.063958 -0.451347
H -0.987120 -0.401696 -0.292980
H -0.438966 -0.499056 2.165788
H -0.115566 1.251830 2.019412
H 1.097240 -1.769224 0.039812
H 1.938896 -0.447053 -0.810469
C 2.266128 -0.989496 2.430211
H 2.176473 0.824588 1.279332
C 2.154361 -0.691532 3.815758
C 2.810539 -1.461893 4.766908
C 3.631165 -2.534744 4.387133
C 3.851262 -2.756426 3.011579
C 3.181552 -1.996255 2.060286
H 1.529900 0.138577 4.134843
H 2.681509 -1.227777 5.821395
H 4.128919 -3.143799 5.134818
H 4.533763 -3.537905 2.687964
H 3.363100 -2.189505 1.006016

14
cyclobutane X= $BH_2^{\cdot -}$ scf done: -182.683259
C -0.041159 -0.053984 -0.002286
C -0.011058 -0.020871 1.550599
C 1.557609 -0.060539 1.497421
C 1.444400 -0.508488 -0.003154
H -0.149785 0.949858 -0.419084
H -0.773177 -0.716934 -0.479956
H -0.452079 -0.928373 1.986966
H -0.455006 0.857469 2.029518
H 1.538765 -1.595368 -0.138288
H 2.110172 -0.001949 -0.708914
H 1.963068 -0.871236 2.131565
B 2.317576 1.317559 1.776982
H 1.980898 2.006708 2.712444
H 3.373193 1.539758 1.229586

18
cyclobutane X= BH_2 **and Y= CH=** CH_2 scf done: -260.107246

C	-0.170539	-0.029758	0.188091
C	0.096222	-0.136261	1.723644
C	1.623966	-0.017242	1.432805
C	1.332579	-0.378047	-0.056402
H	-0.359401	1.013699	-0.084819
C	-1.212305	-0.910047	-0.415948
H	-0.182802	-1.120633	2.109587
H	-0.362704	0.628180	2.354162
H	1.466370	-1.443159	-0.264796
H	1.848370	0.195759	-0.829213
H	2.235151	-0.777579	1.960550
B	2.410941	1.262889	1.805499
C	-2.226893	-0.499925	-1.176386
H	-1.110811	-1.974263	-0.200853
H	-2.951053	-1.195930	-1.584793
H	-2.367938	0.549751	-1.416937
H	2.163326	1.858096	2.812546
H	3.329854	1.629573	1.134046

16

Bh2-butane-nh2 scf done: -238.062943

C	0.051169	0.141004	-0.345113
C	0.018475	0.540106	1.205249
C	1.435840	-0.097257	1.348148
C	1.585520	-0.078257	-0.188617
H	-0.241938	0.964401	-0.998221
B	-0.897505	-1.075267	-0.394617
H	-0.776300	0.137492	1.842083
H	0.028387	1.627548	1.317557
H	2.026818	-0.961223	-0.655208
H	2.149235	0.802510	-0.511900
H	1.339427	-1.124688	1.708459
N	2.489319	0.539349	2.112179
H	-2.040095	-0.940064	-0.716136
H	-0.535628	-2.148752	-0.009890
H	2.330849	0.475865	3.112311
H	2.590055	1.519911	1.869578

14

cyclobutane X= BH_2 and Y= O^- scf done: -257.343815

C	0.145095	0.191356	-0.268766
C	-0.060793	0.163979	1.332941
C	1.514033	-0.219086	1.482989
C	1.647085	-0.128155	-0.101623
H	-0.071042	1.188526	-0.656992
B	-0.954223	-0.843224	-0.453714
H	-0.735883	-0.600120	1.751318
H	-0.335878	1.141717	1.736027
H	1.997630	-1.036622	-0.609536
H	2.297645	0.709743	-0.367771

H	1.528279	-1.310480	1.767817
O	2.237240	0.569792	2.240131
H	-2.093232	-0.496567	-0.631499
H	-0.735632	-2.019348	-0.341201

14
cyclobutane X= BH_2 and Y= S^- scf done: -580.348851

C	0.088851	0.169423	-0.301855
C	-0.042892	0.199963	1.319624
C	1.438008	-0.282837	1.418434
C	1.605112	-0.104230	-0.123013
H	-0.165851	1.138550	-0.734567
B	-0.952396	-0.929099	-0.456537
H	-0.782611	-0.458580	1.798234
H	-0.205232	1.218416	1.676479
H	2.003900	-0.969505	-0.664701
H	2.219358	0.770183	-0.346695
H	1.447282	-1.348195	1.674379
S	2.508623	0.639650	2.551629
H	-2.106280	-0.647526	-0.643082
H	-0.675548	-2.084702	-0.288208

15
cyclobutane X= BH_2 and Y= SH scf done: -580.906584

C	0.020359	0.002481	-0.027651
C	0.019329	0.031583	1.535871
C	1.568726	-0.068913	1.515286
C	1.506517	-0.475668	0.017090
H	-0.005626	1.045084	-0.394463
B	-1.082518	-0.697659	-0.856434
H	-0.425531	-0.870275	1.967627
H	-0.417239	0.904438	2.022690
H	1.603590	-1.557605	-0.103499
H	2.212844	0.009731	-0.659042
H	2.020027	-0.792150	2.193934
S	2.453310	1.536199	1.691048
H	-2.223243	-0.655501	-0.501467
H	-0.822907	-1.251755	-1.883312
H	2.082485	1.789602	2.963850

15
cyclobutane X= BH_2 and Y= OH scf done: -257.934212

C	0.035182	0.032440	0.002213
C	0.018990	0.061290	1.566884
C	1.570101	-0.022053	1.555097
C	1.530008	-0.425775	0.066463
H	0.000223	1.072029	-0.372080
B	-1.045456	-0.681160	-0.843191

H	-0.438211	-0.833611	1.998352
H	-0.408622	0.942621	2.050837
H	1.647995	-1.502762	-0.074984
H	2.247711	0.098501	-0.565643
H	2.025547	-0.739274	2.247769
O	2.209351	1.248086	1.666976
H	-2.187497	-0.691963	-0.488638
H	-0.767704	-1.192589	-1.887585
H	2.072503	1.583810	2.559059

16

cyclobutane X= BH_2 and Y= BH_2 $^{\cdot-}$ scf done: -208.141634

C	0.022406	0.111735	-0.140438
C	-0.001601	0.151888	1.443064
C	1.569131	-0.104643	1.498411
C	1.515940	-0.361182	-0.052864
H	-0.026467	1.139019	-0.518548
B	-1.047721	-0.817395	-0.795448
H	-0.576302	-0.666128	1.894109
H	-0.352971	1.096739	1.871647
H	1.640433	-1.418222	-0.315273
H	2.223988	0.224326	-0.649460
H	1.800036	-1.011844	2.068789
B	2.382562	1.123212	2.011428
H	-1.964104	-0.391846	-1.453959
H	-0.994296	-2.008521	-0.605029
H	2.991375	1.099674	3.051901
H	2.371218	2.155866	1.387531
